# Efficacy and safety of temporary selective portal vein embolization before major hepatectomy (EMBORES): a phase 2 trial

**DOI:** 10.1016/j.eclinm.2026.104262

**Published:** 2026-10-12

**Authors:** Hadrien Tranchart, Sophie Maitre, Edouard Girard, Julien Ghelfi, Maximiliano Gelli, Lambros Tselikas, Maxime Barat, David Fuks, Anthony Sarran, Stéphane Benoist, Olivier Meyrignac, Ailton Sepulveda, Maxime Ronot, Maryne Pou, Mircea Chirica, Ibrahim Dagher, EMBORES Study Group, Christian Sengel, Antoine Brouquet, Brice Gayet, Valérie Vilgrain, François Cauchy, Olivier Soubrane, Leonor Benhaim, Dimitri Tzanis:, Yves Menu, Sébastien Gaujoux, Laurence Rocher, Georges Rafek:, Pierre Chague, Thierry De Baere, Diane Goere, Mickaël Lesurtel, Panagiotis Lainas, Martin Gaillard, Joseph Derienne, Marco Dioguardi Burgio, Annie Sibert, Alain Abdo, Teodor Vasile, Severine Foucher, Marie Chalopin, Thibault Chouquet, Sandra Camps, Steve Suzzoni, Pierrick Bedouch, Marylise Adechian, Hélène Agostini, Lamiae Grimaldi, Kawther Nheri, Fatah Tidadini, Olivier Soubrane, Jean-Baptiste Debry

**Affiliations:** a Department of Minimally Invasive Digestive Surgery, Antoine Béclère Hospital, AP-HP, Clamart, France; b Department of Radiology, Antoine Béclère Hospital, AP-HP, Clamart, France; c Department of Digestive Surgery, CHU Grenoble Alpes, La Tronche, France; d Department of Radiology, CHU Grenoble Alpes, La Tronche, France; e Department of Anesthesiology, Surgery and Interventional Radiology, Gustave Roussy, Villejuif, France; f Department of Radiology, Cochin Hospital, AP-HP, Paris, France; g Department of Digestive, Oncologic and Metabolic Surgery, Institut Mutualiste Montsouris, Paris, France; h Department of Radiology, Institut Mutualiste Montsouris, Paris, France; i Department of Oncologic and Digestive Surgery, Bicêtre Hospital, AP-HP, Le Kremlin-Bicêtre, France; j Department of Radiology, Bicêtre Hospital, AP-HP, Le Kremlin-Bicêtre, France; k Department of HPB Surgery and Liver Transplantation, Beaujon Hospital, AP-HP, Clichy, France; l Department of Radiology, Beaujon Hospital, AP-HP, Clichy, France; m Paris-Saclay University, Orsay, France; n University Grenoble Alpes, Saint-Martin-d’Hères, France; o Paris-Cité University, Paris, France

**Keywords:** Portal vein embolization, Liver regeneration, Future liver remnant, Hepatectomy, Interventional radiology, Phase II clinical trial

## Abstract

**Background:**

Portal vein embolization (PVE) is routinely used before major hepatectomy to induce hypertrophy of the future liver remnant (FLR) and reduce post-hepatectomy liver failure. Conventional PVE relies on permanent embolic agents, resulting in irreversible portal occlusion, which may be associated with complications and may limit subsequent therapeutic options in patients who do not ultimately undergo resection. Whether transient portal deprivation alone can induce clinically relevant liver regeneration remains unknown. We evaluated the efficacy and safety of temporary selective PVE using an absorbable gelatin agent.

**Methods:**

EMBORES was a multicenter prospective single-arm phase II trial conducted in six French hepatobiliary centers. Between May 3, 2018, and May 3, 2021, adult patients requiring major hepatectomy and preoperative PVE were included. Selective portal branches were embolized using powdered absorbable gelatin sponge. The primary endpoint was FLR hypertrophy assessed by computed tomography (CT) volumetry 4–6 weeks after PVE. Secondary endpoints included feasibility, morbidity, portal vein recanalization, and surgical outcomes. This trial is registered with ClinicalTrials.gov, NCT02945059.

**Findings:**

34 patients were enrolled and 32 underwent temporary PVE. Mean age was 63.4 years (standard deviation [SD] 8.9), and colorectal liver metastases accounted for 18 (56%) of 32 indications. Technical success was achieved in all patients. One major complication occurred (1 [3.1%] of 32 patients), while liver function remained preserved. Complete and partial portal vein recanalization were observed in 17 (53%) and 9 (28%) of 32 patients, respectively. Mean FLR volume increased from 580 (SD 169) mL to 690 (SD 188) mL, corresponding to a mean hypertrophy ratio of 9.5% (SD 10.2). Five patients required repeat embolization using a permanent agent because of insufficient hypertrophy. Overall, 23 (71.8%) of 32 patients underwent major hepatectomy, with no postoperative mortality.

**Interpretation:**

Temporary PVE using an absorbable embolic agent is feasible, safe, and capable of inducing clinically meaningful liver hypertrophy before major hepatectomy, supporting the concept that transient portal vein occlusion may be sufficient to stimulate liver regeneration in selected patients.

**Funding:**

The EMBORES study was funded by the French Ministry of Health through a national hospital clinical research program grant awarded in 2015 (Programme Hospitalier de Recherche Clinique National, PHRC-15-15-0201).

**Research in context**

**Evidence before this study**

We searched PubMed from database inception to June 15, 2026, using combinations of the terms “portal vein embolization”, “temporary portal vein embolization”, “absorbable embolic agent”, “gelatin sponge”, “gelfoam”, “liver regeneration”, and “future liver remnant”, without language restrictions. Permanent portal vein embolization using non-absorbable embolic agents is the established standard for inducing hypertrophy of the future liver remnant before major hepatectomy. Experimental studies and a conceptual editorial suggested that transient portal deprivation might be sufficient to trigger liver regeneration. Before initiation of the present study, clinical evidence was limited to a single retrospective human series evaluating temporary portal vein embolization with absorbable gelatin sponge powder. No prospective multicentre trial had assessed the efficacy and safety of a fully absorbable portal vein embolization strategy in patients undergoing major hepatectomy.

**Added value of this study**

To our knowledge, EMBORES is the first prospective multicentre phase II study evaluating temporary portal vein embolization using a fully absorbable embolic agent in patients undergoing preparation for major hepatectomy. Temporary PVE was technically feasible and safe, achieved clinically meaningful hypertrophy of the future liver remnant, and allowed major hepatectomy in most patients. Liver regeneration occurred despite partial or complete portal vein recanalization in most patients, suggesting that sustained portal occlusion might not be necessary to trigger a clinically relevant regenerative response.

**Implications of all the available evidence**

Taken together, the available experimental and clinical evidence suggests that transient portal flow deprivation can induce durable liver regeneration. These findings challenge the traditional assumption that permanent portal occlusion is required to achieve adequate hypertrophy before major hepatectomy. Temporary PVE may represent a reversible strategy for preoperative liver conditioning and could broaden future approaches to portal flow modulation. Comparative studies against conventional permanent PVE are now required to define the clinical role of this approach and identify patient populations most likely to benefit.

## Introduction

Portal vein embolization (PVE) is commonly performed before major hepatectomy to induce hypertrophy of the future liver remnant (FLR), thereby reducing the risk of post-hepatectomy liver failure (PHLF).^1^, ^2^, ^3^ Permanent embolic agents are generally preferred because they provide durable portal vein occlusion and reliable FLR hypertrophy. However, although uncommon, permanent embolization carries a risk of unintended embolization of the contralateral portal vein or extension of thrombosis into the portal or mesenteric venous system.^4^^,^^5^ Such complications may compromise liver regeneration, complicate patient management, and occasionally preclude surgical resection. Moreover, permanent portal occlusion may limit subsequent therapeutic options in patients who ultimately do not undergo hepatectomy. Therefore, the use of absorbable embolic materials may represent a safer alternative while preserving the regenerative stimulus induced by portal flow deprivation.

In a nonhuman primate model, our group previously demonstrated that transient portal deprivation using a reversible embolization strategy could induce durable liver hypertrophy despite subsequent portal vein recanalization, suggesting that permanent portal vein occlusion may not be essential to induce sustained liver regeneration.^6^^,^^7^ These findings were subsequently supported by a retrospective clinical series suggesting that temporary PVE with absorbable gelatin could induce clinically relevant hypertrophy before major hepatectomy.^8^ However, whether transient portal vein occlusion alone is sufficient to induce clinically meaningful liver regeneration in humans had not yet been prospectively evaluated. The present phase II study was therefore designed as a proof-of-concept trial to determine whether transient portal vein occlusion using a fully absorbable embolic agent could induce adequate liver regeneration despite subsequent portal vein recanalization. We therefore initiated the EMBORES trial to prospectively evaluate the efficacy and safety of temporary selective PVE using an absorbable gelatin sponge in patients scheduled for major hepatectomy requiring preoperative PVE.

## Methods

### Study design and patients

EMBORES was a multicentre, single-arm, investigator-initiated phase II trial conducted at six French hepatobiliary centres. Patients were enrolled between May 3, 2018, and May 3, 2021. The protocol was registered at ClinicalTrials.gov (NCT02945059) and is available in the Appendix. Protocol amendments implemented during the study mainly concerned administrative updates, clarification of study procedures, extension of the inclusion period, revision of the sample size following slower-than-expected recruitment, and clarification of the volumetric analysis. A summary of all protocol amendments is provided in the Appendix.

Eligible patients were aged 18–80 years and required preoperative PVE before major hepatectomy (≥3 segments) for primary or secondary liver malignancies. All cases were reviewed in multidisciplinary meetings before treatment decisions were made. Key exclusion criteria included an American Society of Anesthesiologists (ASA) score greater than 3 and extensive portal or hepatic vein thrombosis, pregnancy, and contraindications to angiography or iodinated contrast media.

The primary endpoint was FLR hypertrophy after temporary PVE. Secondary endpoints included technical feasibility, morbidity, portal vein recanalization, and the proportion of patients proceeding to hepatectomy. All endpoints were prespecified in the study protocol before patient enrolment. Intraoperative and postoperative outcomes, including postoperative morbidity and mortality, were also assessed.

### Ethics statement

An independent data safety monitoring board oversaw the study. The study was approved by the Comité de Protection des Personnes (CPP) Île-de-France VII (Research Project No. 16-028; ID-RCB No. 2016-A00878-43) and was conducted in accordance with the Declaration of Helsinki. All participants provided written informed consent.

### Procedures

Embolization of selected portal vein segments was performed using the powdered form of an absorbable gelatin (Curaspon®; CuraMedical B.V, Assendelft, Netherlands) without the use of coils. Gelatin powder was dissolved in a solution of 80% iodixanol (Visipaque® 270 mg/mL; GE Healthcare, Chicago, USA) and 20% saline (final concentration of 0.08 g/mL) to suspend the particles of absorbable gelatin powder completely and to obtain a homogeneous mix visible during the embolization.

The embolization technique has been previously described in detail.^8^ Briefly, percutaneous PVE was performed using either an ipsilateral or contralateral approach according to operator preference. The embolic mixture was selectively injected into the targeted portal branches until complete angiographic occlusion was achieved while preserving flow to the FLR. The amount of absorbable gelatin powder was not standardized and depended on the portal venous anatomy and extent of embolization. As an indication, approximately 4 g of absorbable gelatin powder was generally required for right portal vein embolization. All participating interventional radiologists received dedicated training before study initiation, and patient enrolment was restricted to centres with certified expertise in liver-directed interventions.

### Statistical analysis

All patients who underwent PVE were included in the intention-to-treat population. FLR hypertrophy was assessed by CT volumetry performed before PVE and 4–6 weeks after the procedure. To minimize measurement bias, all imaging data were anonymized and centrally reviewed by an independent blinded observer. Total liver volume, embolized liver volume, non-embolized liver volume (FLR), and portal vein recanalization were assessed. The hypertrophy ratio was defined as the increase in standardized FLR volume relative to functional liver volume, excluding tumour volume. Standardized FLR volume was calculated as FLR volume divided by functional liver volume (total liver volume minus tumour volume) and expressed as a percentage before and after PVE [%FLRpre-PVE = (FLRpre-PVE volume/(total liver volumepre-PVE − tumour volumepre-PVE)) × 100; %FLRpost-PVE = (FLRpost-PVE volume/(total liver volumepost-PVE − tumour volumepost-PVE)) × 100]. The hypertrophy ratio was defined as the difference between post-PVE and pre-PVE standardized FLR volume (%FLRpost-PVE − %FLRpre-PVE). Negative hypertrophy ratios were conservatively analysed as absence of hypertrophy. The kinetic growth rate (KGR) was calculated as the hypertrophy ratio divided by the interval (in weeks) between PVE and follow-up CT. The observed hypertrophy ratio was compared with the reference value of 13% after conventional portal vein embolization. No predefined FLR hypertrophy threshold was used to determine eligibility for hepatectomy. The decision to proceed with surgery was made at multidisciplinary meetings in each participating centre, taking into account FLR volume, underlying liver function, tumour characteristics, and the overall surgical strategy.

Technical feasibility was assessed by the interventional radiologist performing the procedure and classified as complete, partial, or unsuccessful. For descriptive purposes, embolization was classified as distal-only (segmental and subsegmental portal branches), proximal-only (sectorial portal branches), or combined distal and proximal embolization. This classification reflected the anatomical distribution of the radiopaque embolic material rather than the level of portal vein occlusion. Complete angiographic occlusion of the targeted portal territory was achieved irrespective of whether the embolic material was distributed distally, proximally, or at both levels. Procedure-related morbidity was monitored during the intervention and throughout the 6-week follow-up period. Adverse events were prospectively recorded at each study visit throughout follow-up and evaluated together with procedure-related morbidity and liver function tests according to the study protocol. Blood tests, including liver function tests and prothrombin time, were performed at baseline, day 1, and day 7. Clinical and radiological reassessment was done 4–6 weeks after PVE. The proportion of patients proceeding to hepatectomy was recorded.

Surgery was generally performed 4–8 weeks after PVE. Intraoperative morbidity and portal pedicle adhesions were assessed using Zühlke’s classification.^9^ Postoperative complications occurring within 90 days were graded according to the Clavien–Dindo classification.^10^ PHLF was defined using the “50–50” criterion (prothrombin time <50% and total bilirubin >50 μmol/L). Patients were followed clinically, biologically, and radiologically for up to 12 months after PVE.

Statistical analyses were performed according to a prespecified statistical analysis plan by a biostatistician from the Clinical Research Unit (URC) Paris-Sud/Paris-Saclay using SAS software (Cary, NC, USA). Quantitative variables were tested for normality using the Shapiro–Wilk test and are presented as mean (standard deviation [SD]) or median (interquartile range [IQR]), as appropriate, and qualitative variables as frequencies and percentages. The hypertrophy ratio following temporary PVE was reported with its 95% CI and compared with the historical reference value of 13%, previously reported by Elias et al. after conventional PVE using cyanoacrylate and ethiodized oil.^11^ This comparison formed the basis for the sample size calculation. We hypothesized that temporary PVE would result in a comparable increase in FLR volume. Assuming an expected hypertrophy ratio of 9% and an SD of 6·5%, 44 evaluable patients were required to estimate the mean hypertrophy ratio with a 95% CI of ±2%. Allowing for 10% non-evaluable patients, the planned sample size was 50 patients. No imputation of missing data was performed, and analyses were conducted on available data.

### Role of the funding source

The funder and sponsor had no role in the study design, data collection, data analysis, data interpretation, writing of the report, or the decision to submit the manuscript for publication. The corresponding author had full access to all study data and had final responsibility for the decision to submit for publication.

## Results

### Patient population

Overall, 34 patients were enrolled across six French centers. Recruitment proved slower than anticipated, and despite protocol amendments extending the inclusion period, the planned target of 44 evaluable patients could not be reached because further extension of the study was not feasible owing to funding constraints. Two patients were excluded from the efficacy analysis: one withdrew consent before embolization, and another could not undergo embolization due to technical difficulties in catheterizing the portal branches, unrelated to the temporary embolization material or technique; this failed attempt had no adverse consequences. Thus, 32 patients finally underwent temporary PVE and were evaluable for the volumetric analysis (Fig. 1). The mean age was 63.4 years (SD 8.9), 25 (78%) of 32 patients were male, and the surgical indication was colorectal liver metastases in 18 (56%) patients, hepatocellular carcinoma in 8 (25%), cholangiocarcinoma (including one perihilar) in 5 (16%), and metastatic nasopharyngeal carcinoma in 1 (3%). All hepatocellular carcinomas arose in cirrhotic livers secondary to alcohol, HBV, or HCV. In 25 (78%) of 32 patients, tumour lesions were confined to the right liver, whereas in the remaining 7 (22%) patients they involved both the right and left liver, or extended to the biliary confluence. The median number of liver tumours was 3 (IQR 1–7). Among the 18 patients with colorectal liver metastases, the primary tumour was still in situ in 11 patients, whereas seven had previously undergone resection of the primary tumour. All patients managed for hepatic metastases received first-line chemotherapy. A patient with hepatocellular carcinoma was initially treated with transarterial chemoembolization. The extent of liver treated by transarterial chemoembolization was not prospectively recorded and could therefore not be evaluated as a potential determinant of liver regeneration. The demographic and clinical characteristics at baseline of the patients that finally underwent temporary PVE are summarized in Table 1.

**Fig. 1:**
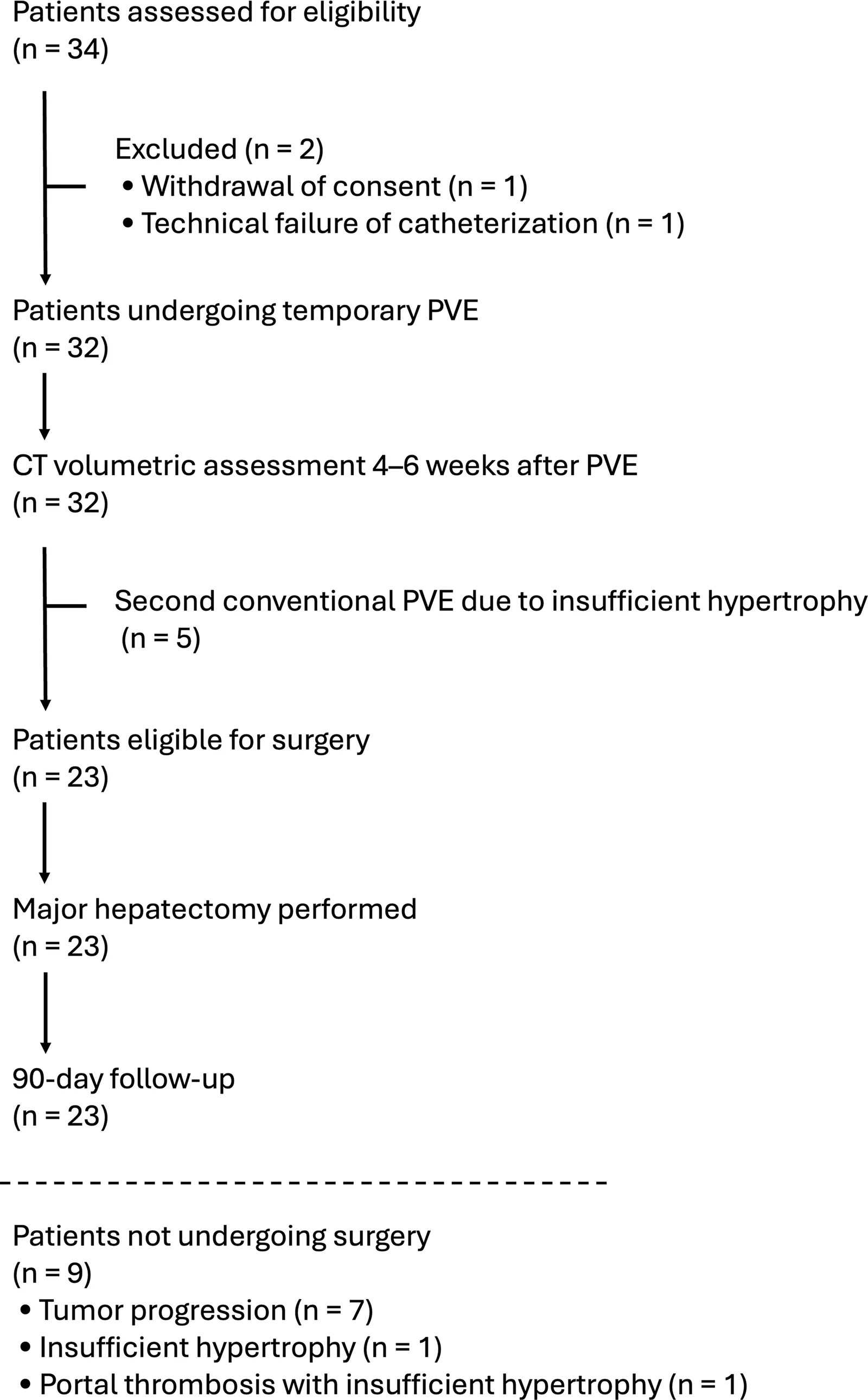
Study flowchart. CT: computed tomography; PVE: portal vein embolization.

**Table 1:** Baseline demographic and clinical characteristics of the study population (n = 32).

Variable	Value
Age (years), mean ± SD	63.4 ± 8.9
Gender, n (%)	
Male	25 (78.1)
Female	7 (21.9)
Weight (kg), mean ± SD	73.4 ± 14.2
Height (cm), mean ± SD	172 ± 8.3
Body Mass Index (kg/m^2^), mean ± SD	24.9 ± 4.3
Creatinine clearance (ml/min), mean ± SD	89.8 ± 27.2
Surgical indication, n (%)	
Colorectal liver metastases	18 (56.2)
Hepatocellular carcinoma	8 (25.0)
Cholangiocarcinoma	5 (15.6)
Nasopharyngeal carcinoma liver metastasis	1 (3.1)
Cirrhosis, n (%)	8 (25)
ASA class I, II, III, n (%)	5 (15.6), 20 (62.5), 7 (21.9)
Previous surgery, n (%)	24 (75.0)
First line chemotherapy, n (%)	20 (62.5)
Number of prior chemotherapy lines, 1, 2 or 3, n (%)	8 (40), 10 (50), 2 (10)
Anticoagulant therapy, n (%)	4 (12.5)
Antiplatelet therapy, n (%)	3 (9.4)
Tumour location n (%)	
Right liver	25 (78.1)
Right and left liver	7 (21.9)
Number of liver tumours, median [IQR]	3 [1–7]

SD: standard deviation, ASA: American Society of Anesthesiologists, IQR: interquartile range.

### Procedure, safety and tolerability

Temporary PVE was performed in 32 patients, with a median procedure time of 90 [70–120] minutes. In 31 (97%) of 32 patients, PVE involved the right portal branch, whereas in 1 (3%) patient, it involved both the left portal and right paramedian branches. The planned embolization was achieved in all patients. The PVE was classified by the radiologists as combined distal and proximal embolization in 20 (62%) of 32 patients, distal-only in 9 (28%), and proximal-only in 3 (9%). Liver function remained globally preserved after PVE, with only mild and transient biochemical abnormalities (Fig. 2).

**Fig. 2:**
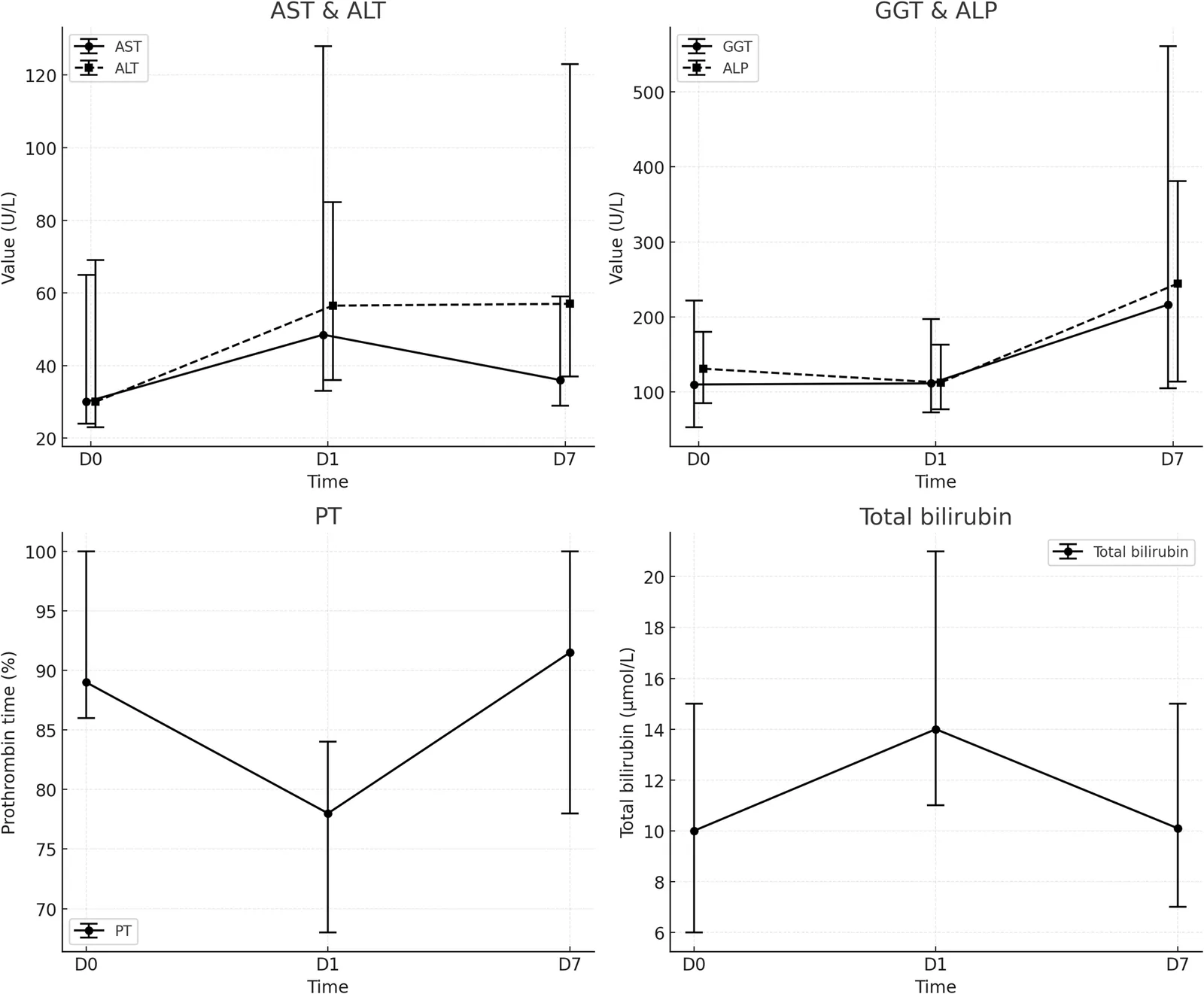
Evolution of liver function tests after portal vein embolization (PVE). AST: aspartate aminotransferase; ALT: alanine aminotransferase; γ-glutamyltransferase: GGT; alkaline phosphatase: ALP; PT: prothrombin time; D: day.

Intra-procedural morbidity occurred in 6 (18.7%) of 32 patients and consisted mainly of pain, nausea, or transient fever. No patient developed liver failure or sepsis after PVE. One (3.1%) of 32 patients, treated for bilobar colorectal liver metastases, with a history of multiple hepatic resections and local ablative procedures, developed portal vein thrombosis extending to the contralateral portal branch and superior mesenteric vein after embolization. Despite partial recanalization, the persistence of thrombosis and insufficient FLR hypertrophy precluded hepatectomy, and the patient was subsequently managed with palliative chemotherapy. Procedural safety and tolerability data are summarized in Table 2.

**Table 2:** Procedural safety and patient tolerance following temporary portal vein embolization (n = 32).

Variable	Value
Time from inclusion to embolization (days), median [IQR]	1 [1–8.5]
Procedure duration (minutes), median [IQR]	90.5 [70.5–120]
Type of embolization, n (%)	
Right	31 (96.9)
Left and right paramedian	1 (3.1)
Planned complete, n (%)	32 (100)
Type of occlusion, n (%)	
Proximal and distal	20 (62.5)
Proximal	3 (9.4)
Distal	9 (28.1)
Intra-procedural morbidity, n (%)	6 (18.7)
Abdominal pain	4 (66,7)
Fever	1 (16.7)
Nausea	1 (16.7)
Post-procedural morbidity^a^, n (%)	1 (3.1)

IQR: interquartile range.

a Portal vein thrombosis extension.

### Liver regeneration and portal recanalization after embolization

Abdominal CT was performed after a mean interval of 30 days (SD 6) following PVE. Complete and partial portal vein recanalization were observed in 17 (53%) and 9 (28%) of 32 patients, respectively, whereas no recanalization was observed in 6 (19%) patients. Despite complete or partial portal vein recanalization in 26 (81%) of 32 patients, clinically meaningful FLR hypertrophy was maintained. Volumetric analysis demonstrated a significant increase in the FLR. Mean FLR volume increased from 580 mL (SD 169) pre-PVE to 690 mL (SD 188) post-PVE, corresponding to a mean hypertrophy rate of 23.6% (SD 27.9) and a median of 20.5% (IQR 6.2–38.4). The median KGR was 1.94%/week (IQR 0.96–2.81). The mean FLR volume represented 40.9% (SD 10.9) of the total liver volume after PVE. The mean hypertrophy ratio was 9.5% (SD 10.2; 95% CI 5.8–13.1), compared with the historical reference value of 13% (P = 0.059). Before PVE, 12 (37.5%) of 32 patients had an FLR < 30%, compared with 3 (9.4%) of 32 patients after PVE. Similarly, 23 (71.9%) of 32 patients had an FLR < 40% before PVE, decreasing to 12 (37.5%) of 32 patients after PVE. The mean hypertrophy ratio was 5.7% (SD 5.6; 95% CI 0.9–10.4) in cirrhotic patients, which was significantly lower than the historical reference value of 13% (P = 0.023). In contrast, non-cirrhotic patients had a mean hypertrophy ratio of 10.7% (SD 11.1; 95% CI 6.3–15.4), with no statistically significant difference from the historical reference value of 13% (P = 0.327) (Fig. 3). Exploratory analyses of liver hypertrophy according to portal vein recanalization status and of recanalization according to embolization technique are summarized in Supplementary Table S1. Given the small subgroup sizes, these findings should be interpreted with caution. Five (15.6%) of 32 patients required a second PVE using a conventional technique because of insufficient FLR hypertrophy. Among these patients, three were treated for colorectal liver metastases, one for hepatocellular carcinoma arising in a cirrhotic liver, and one for cholangiocarcinoma. The second conventional embolization was performed without technical difficulties.

**Fig. 3:**
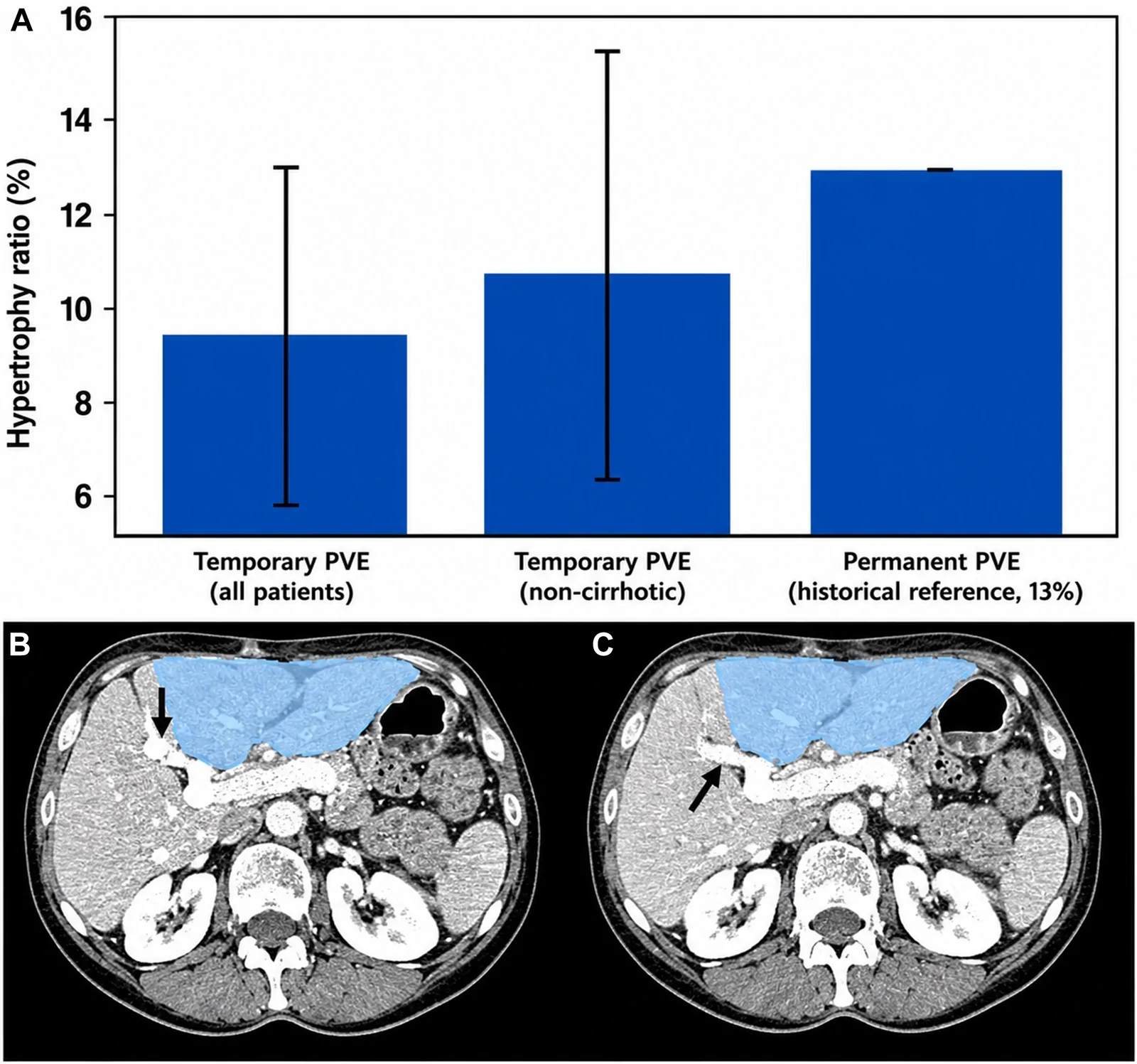
(A) Hypertrophy ratio of the future liver remnant after temporary portal vein embolization (PVE) in the overall cohort and in the non-cirrhotic subgroup, compared with the reference value reported after permanent PVE. (B and C) Computed tomography images of the future liver remnant (blue area) before (B) and one month after (C) temporary PVE in a patient undergoing right hepatectomy for metastatic liver disease. The black arrow in panel B indicates the middle hepatic vein, delineating the right and left liver. The black arrow in panel C indicates the right portal branch, showing complete recanalization one month after PVE.

### Surgical and oncological outcomes

Overall, liver hypertrophy achieved after temporary PVE was considered sufficient to proceed with surgery in most patients. Twenty-three (71.8%) of 32 patients subsequently underwent hepatectomy after a median interval of 50 days following PVE (IQR 35–77). The mean operative time was 357 min (SD 98), with a median blood loss of 600 mL (IQR 400–900); 4 (17%) of 23 patients required blood transfusion. Adhesions around the portal pedicle were absent or filmy in 17 (74%) of 23 patients, moderate in 5 (22%), and severe in 1 (4%). Intraoperative morbidity occurred in 5 (21.7%) of 23 patients, including three major bleeding events, one pneumothorax, and one case of gas embolism. As commonly observed after major hepatectomy, liver function tests were transiently impaired in the postoperative period. Their postoperative evolution is shown in Fig. 4. The overall postoperative morbidity rate at 90 days was 47.8% (11 of 23 patients), with liver-specific complications occurring in 7 (30.4%) of 23 patients, including transient ascites (n = 4), PHLF (n = 2), and biliary leakage (n = 1). Of the two patients who developed PHLF, one had no volumetric liver growth after PVE (KGR 0%/week), whereas the other had a KGR of 3.96%/week. No postoperative mortality was observed within 90 days. Specifically, among the five patients who required a second PVE using a conventional approach, four ultimately underwent surgery, and none developed PHLF. Histological changes attributable to prior PVE were observed in only three resected specimens. Surgical outcomes are summarized in Table 3.

**Fig. 4:**
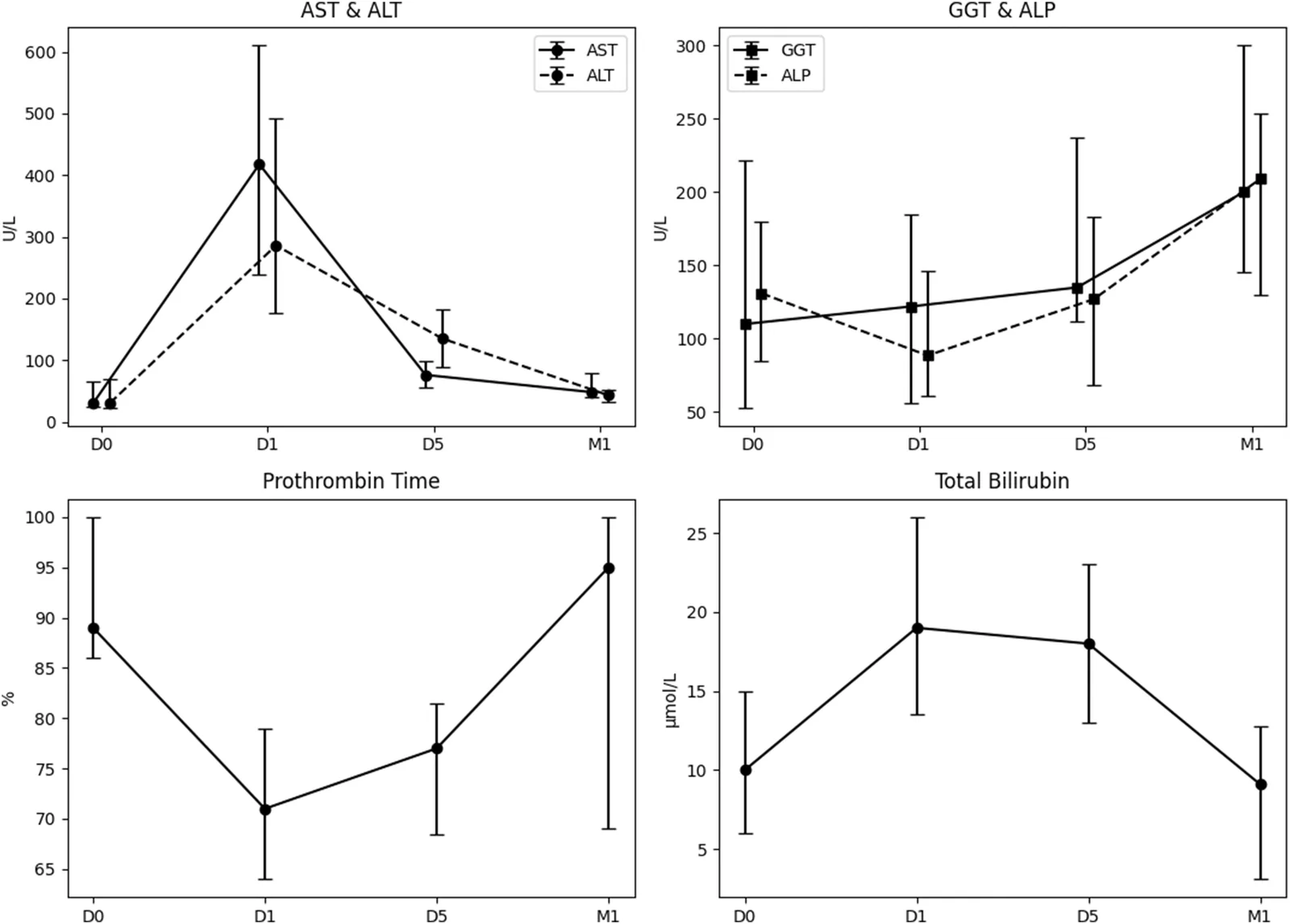
Evolution of liver function tests after major hepatectomy. AST: aspartate aminotransferase; ALT: alanine aminotransferase; γ-glutamyltransferase: GGT; alkaline phosphatase: ALP; PT: prothrombin time; D: day.

**Table 3:** Surgical outcomes after major hepatectomy following temporary portal vein embolization (n = 23).

Variable	Value
Interval from embolization to hepatectomy (days), median [IQR]	50 [35–77]
Operation time (minutes), mean ± SD	357 ± 98
Type of major liver resection, n (%)	
Right hepatectomy	16 (69.6)
Right lobectomy	5 (21.7)
Right lobectomy + segment I resection	2 (8.7)
Surgical approach, n (%)	
Laparotomy	13 (56.5)
Laparoscopy	10 (43.5)
Portal pedicle adhesions^a^, n (%)	
Grade 0	8 (34.8)
Grade 1	9 (39.1)
Grade 2	5 (21.7)
Grade 3	1 (4.3)
Blood loss (mL), median [IQR]	600 [400–900]
Blood transfusion, n (%)	4 (17.4)
Intraoperative morbidity, n (%)	5 (21.7)
Overall postoperative morbidity (at 90 days), n (%)	11 (47.8)
Dindo-Clavien Grade ≥ IIIa, n (%)	3 (27.2)
Liver specific morbidity (at 90 days), n (%)	7 (30.4)
Length of hospital stay (days), median [IQR]	8 [7–15]

IQR: interquartile range, SD: standard deviation.

a According Zühlke’s classification.

Nine (28.1%) of 32 patients did not undergo surgery, mainly due to disease progression (n = 7), insufficient hypertrophy (n = 1), or a combination of portal vein thrombosis extension and insufficient hypertrophy (n = 1). During follow-up, three of these patients died. Among patients who underwent hepatectomy, one death occurred during follow-up, 9 months after surgery, and was related to tumour progression.

## Discussion

This is the first prospective multicentre phase II study demonstrating that temporary PVE using an absorbable agent is feasible, safe, and capable of inducing clinically meaningful FLR hypertrophy before major hepatectomy. Although permanent embolization remains the standard approach, our findings suggest that sustained portal occlusion may not always be necessary to trigger a clinically relevant regenerative response. Liver hypertrophy occurred despite partial or complete portal vein recanalization in most patients, consistent with previous experimental studies and preliminary clinical observations^6^, ^7^, ^8^^,^^12^, ^13^, ^14^ Because recanalization was assessed on CT performed approximately one month after PVE, later spontaneous recanalization may have occurred in additional patients who underwent surgery shortly thereafter and therefore could not be evaluated over a longer follow-up. These findings suggest that temporary portal deprivation may be sufficient to induce clinically meaningful liver regeneration, and support the concept of reversible portal flow modulation as a potential alternative strategy for preoperative liver conditioning.

Temporary PVE using an absorbable agent achieved a mean FLR hypertrophy ratio of 9.5%, with no statistically significant difference from the historical reference value of 13% reported after permanent PVE. Although comparisons across studies should be interpreted cautiously because of differences in patient populations, embolic agents, volumetric endpoints, and methods used to quantify hypertrophy, the degree of hypertrophy observed in the present study is within the range reported after conventional PVE.^4^^,^^15^^,^^16^ These findings are also consistent with the work of de Baere et al., who demonstrated that clinically relevant liver hypertrophy can be achieved with different embolic materials, although the magnitude of hypertrophy may vary according to the embolic agent used.^1^ Furthermore, the high rate of portal vein recanalization observed after temporary PVE confirms our previous retrospective observations and indicates that restoration of portal flow does not preclude clinically meaningful liver regeneration.^8^ This degree of hypertrophy was sufficient to allow major hepatectomy in most patients and supports the potential role of temporary PVE as an alternative strategy in selected clinical settings.

The relationship between portal vein recanalization and liver regeneration deserves specific consideration. A recent retrospective study by Uemura et al. reported lower liver hypertrophy and KGR in patients with major portal vein recanalization after PVE with gelatin sponge particles.^17^ Interestingly, the median degree of hypertrophy in patients without recanalization was 9.3%, which was similar to the mean hypertrophy ratio of 9.5% observed in the present study despite partial or complete recanalization in 26 (81%) of 32 patients. Differences in the physical characteristics and distribution of the embolic material may contribute to these apparently divergent findings. Although Uemura et al. described the use of gelatin sponge particles, a previous report from the same institution, with several overlapping authors, described their PVE technique using gelatin sponge fragments prepared from gelatin sponge sheets.^18^ This differs from the gelatin powder used in EMBORES and may result in a different intraportal distribution and recanalization kinetics. Interestingly, in a canine model, Huang et al. used larger gelatin sponge strips (10 × 1 mm); these initially achieved complete portal occlusion but were associated with recanalization at 4 weeks and no significant compensatory hypertrophy.^19^ The timing of recanalization assessment also differed substantially between the clinical studies. Uemura et al. assessed portal vein recanalization by CT 2 weeks after PVE,^17^ whereas in EMBORES CT was performed after a mean interval of 30 days (SD 6). With an absorbable embolic material, portal vein patency is expected to evolve over time. Experimental data further support the hypothesis that the kinetics of recanalization may be important. In the canine study by Huang et al., because no intermediate angiographic assessment was performed, the actual timing of recanalization could not be determined.^19^ By contrast, our previous experimental studies of reversible PVE showed progressive restoration of portal flow during the first 2 weeks after embolization while liver regeneration was maintained.^6^ Taken together, these findings suggest that the biological effect of temporary PVE may depend not simply on the occurrence of recanalization, but on its kinetics and the duration of effective portal deprivation before portal flow is restored.

The hypertrophic response following temporary PVE was reduced in cirrhotic patients. This observation is consistent with the well-established impairment of liver regenerative capacity associated with cirrhosis.^15^^,^^20^^,^^21^ Temporary PVE using absorbable gelatin should be used with caution in patients with cirrhosis until further evidence becomes available. Given the lower hypertrophic response observed in patients with cirrhosis in our cohort, the efficacy of temporary PVE warrants further investigation across the spectrum of underlying liver parenchymal alterations, including varying degrees of fibrosis, steatosis, and chemotherapy-associated liver injury.

An important finding of this study is the favourable safety profile of temporary PVE. Major complications after conventional PVE are reported in approximately 2–5% of patients.^4^^,^^22^, ^23^, ^24^ Among the reported complications are those related to irreversible portal occlusion, including unintended embolization or extension of portal thrombosis to the FLR or mesenteric venous system. Only one clinically significant complication occurred (1 [3.1%] of 32 patients), consisting of portal vein thrombosis extending to the contralateral portal branch and superior mesenteric vein. No cases of liver failure, sepsis, or severe infectious complications were observed, and liver function remained largely preserved after embolization. In addition, complete or partial portal vein recanalization occurred in 26 (81%) of 32 patients, supporting the reversible nature of the embolic effect. Together, these findings suggest that temporary PVE is well tolerated and may offer a safe alternative approach to portal flow modulation in selected patients.

Major hepatectomy was ultimately performed in 23 (71.8%) of 32 patients, consistent with resection rates reported after conventional PVE.^4^^,^^15^^,^^16^ An interesting finding was the apparent ease of portal pedicle dissection, which contrasts with previous reports describing inflammatory changes and adhesions after permanent PVE.^15^^,^^25^^,^^26^ This observation may reflect the limited inflammatory reaction associated with temporary portal occlusion. Postoperative outcomes remained within the range reported in the literature, with 90-day morbidity occurring in 11 (47.8%) of 23 patients and only two cases of PHLF.^25^^,^^27^ These postoperative events should be interpreted in the context of the complexity of major hepatectomy and the underlying characteristics of the study population, rather than being solely attributed to the embolization procedure. Although volumetric hypertrophy is an important component of preoperative assessment, volume gain does not necessarily reflect a proportional increase in functional liver reserve. Given the occurrence of PHLF in two patients, future studies of temporary PVE should combine volumetric assessment with functional evaluation of the FLR, for example using 99mTc-mebrofenin hepatobiliary scintigraphy with single-photon emission computed tomography (SPECT)/CT. Overall, our surgical outcomes are consistent with previous reports of temporary PVE using absorbable gelatin, in which sufficient hypertrophy allowed hepatectomy in most patients without compromising postoperative outcomes.^8^

The reversible nature of temporary PVE may broaden future approaches to portal flow modulation in liver surgery. This approach may be particularly attractive in patients who require only a modest increase in FLR volume to safely undergo major hepatectomy, in whom temporary portal vein occlusion may provide sufficient regenerative stimulus while allowing subsequent restoration of portal flow. As previously suggested,^28^ reversible modulation of portal inflow could have applications beyond conventional preoperative liver conditioning. Because temporary PVE preserves the possibility of spontaneous portal vein recanalization, it may be particularly attractive in clinical situations where permanent portal occlusion could compromise subsequent therapeutic options. Further studies are required to define the clinical role of this strategy and identify the patients most likely to benefit.

This study has several limitations. First, despite encouraging results, the sample size was limited and the planned enrolment target was not reached, reducing the statistical power of the analysis. Consequently, the study should be considered hypothesis-generating, and the comparison with the historical reference value should be interpreted with caution. Second, the multicentre design may have introduced heterogeneity in patient selection, procedural techniques, and perioperative management. Because of the limited sample size, no subgroup analyses according to tumour type could be performed. Underlying liver parenchymal alterations and tumour types may influence the regenerative response after PVE and should therefore be specifically evaluated in larger future studies. Third, portal vein recanalization was assessed at a single time point, on CT performed approximately one month after PVE. Therefore, the timing and kinetics of recanalization could not be determined. Because most patients subsequently underwent hepatectomy, the longer-term evolution of portal vein patency could not be evaluated. Fourth, the absence of a control group precludes direct comparison with conventional permanent PVE. Consequently, temporary PVE should currently be regarded as an exploratory strategy requiring confirmation in adequately powered comparative prospective studies before its role in clinical practice can be established. Fifth, functional assessment of the FLR was not included in the study protocol, precluding evaluation of the relationship between volumetric hypertrophy and functional liver reserve. Finally, the study was designed to evaluate short-term liver regeneration and perioperative outcomes; therefore, no conclusions can be drawn regarding long-term oncological outcomes, which should be assessed in future comparative studies.

This prospective multicentre phase II study showed that temporary PVE using a fully absorbable embolic agent is feasible, safe, and capable of inducing clinically meaningful liver regeneration before major hepatectomy. The observation that clinically meaningful liver hypertrophy was achieved despite portal vein recanalization suggests that sustained portal occlusion may not always be necessary to induce effective liver regeneration. Further comparative studies are warranted to define the role of temporary PVE in clinical practice.

## Contributors

Conceptualization: HT, SM, ID.

Methodology: HT, SM, ID.

Investigation: all authors.

Formal analysis: HT, ID.

Writing—original draft: HT, DF, MC.

Writing—review & editing: all authors.

Supervision: HT, ID.

HT and ID directly accessed and verified the underlying participant-level data.

## Data sharing statement

Deidentified individual participant data underlying the results reported in this Article, together with the study protocol and statistical analysis plan, will be available upon reasonable request to the corresponding author beginning at publication. Data will be shared with qualified researchers whose proposed use of the data has been approved by the study investigators and the sponsor (Assistance Publique–Hôpitaux de Paris). Requests should include a methodologically sound research proposal. Data access will be granted following review and approval of the proposal and, where applicable, after signature of a data access agreement and compliance with institutional and regulatory requirements.

## Declaration of interests

The authors declare no conflicts of interest.

## Appendix A Supplementary data

EMBORES_protocole-Addendum_v1.1

EMBORES Institutional Review Board

EMBORES_English_Statistical plan

EMBORES PATIENT INFORMATION SHEET

EMBORES Amendment 18-07-2019

EMBORES Amendment 09-01-2019

EMBORES Amendment 10-10-2018

EMBORES Amendment 17-07-2018

EMBORES Amendment 8-06-2020

EMBORES Amendment 27-11-2020

EMBORES Amendment 15-01-2021

EMBORES Amendment 30-03-2022

Supplementary Table S1

Supplementary Material. Members of the EMBORES Study Group
