# Supplementary material for: Efficacy and safety of temporary selective portal vein embolization before major hepatectomy (EMBORES): a phase 2 trial
Source: eClinicalMedicine. 2026 Oct 12;101:104262. doi: 10.1016/j.eclinm.2026.104262 (PMC13681468; doi:10.1016/j.eclinm.2026.104262)
Supplement: Supplementary file 1 [file mmc1.pdf]

« Multicentric prospective study assessing the efficiency of preoperative reversible selective portal vein embolization in patients requiring major hepatic resection»

EMBORES

BIOMEDICAL RESEARCH PROTOCOL RELATING TO MEDICAL DEVICES

Version N°1.1 du 24/10/2017
Project Code : P150953/ N° IDRCB: 2016-A00878-43

Coordinating Investigator: Ibrahim DAGHER
Department of minimally invasive digestive surgery
Antoine Béclère Hospital
Tel. +33145374545
Email : ibrahim.dagher@aphp.fr

Scientific Director : Hadrien TRANCHART
Department of minimally invasive digestive surgery
Antoine Béclère Hospital
Tel.+33662627965
Email: hadrien.tranchart@aphp.fr

Sponsor : AP-HP
and by delegation: Clinical Research and Development Department (DRCD)
Hôpital Saint-Louis
1, avenue Claude Vellefaux
DRCD-Siège project referent: Agnès DORION
Tel. +33144841708
Email : agnes.dorion@aphp.fr

Entity responsible for monitoring research: Unité de Recherche Clinique (URC) Paris-Sud
Address
DRCD-URC (Clinical Research Unit) head:
Hélène AGOSTINI
Tel.+33145217130 Email: helene.agostini@aphp.fr
DRCD-URC (Clinical Research Unit) project referent:
Prisca ASSOHOTO
Tel.+33145212048
Email: prisca.assohoto@aphp.fr

Ce document est la propriété du DRCD / APHP. Toute reproduction est formellement interdite.

SIGNATURE page for a biomedical research PROTOCOL

Research Code: P 150201

Title: Multicentric prospective study assessing the efficiency of preoperative reversible selective portal vein embolization in patients requiring major hepatic resection

Version N° 1.1 of 24/10/2017

The research will be carried out in accordance with the protocol, with current good practices and with the legislative and regulatory provisions in force.

Coordinating Investigator:	
Pr Ibrahim DAGHER	Date: ……………/………/………..
Department of minimally invasive digestive surgery	Signature:
Antoine Béclère Hospital	

Scientific Director:	
Dr Hadrien TRANCHART	Date: ……………/………/………..
Department of minimally invasive digestive surgery	Signature:
Antoine Béclère Hospital	

Sponsor	
Assistance Publique – Hôpitaux de Paris	Date: ……………/………/………..
Clinical Research and Development Department	Signature:
Hôpital Saint Louis	
1 avenue Claude Vellefaux	
75010 PARIS	

The research received a favorable opinion from the CCP Ile de France VII on 07/02/2017 and authorization from the ANSM on 20/10/2017

Ce document est la propriété du DRCD / APHP. Toute reproduction est formellement interdite.

TABLE DES MATIÈRES

1 SUMMARY 6

2 SCIENTIFIC JUSTIFICATION FOR THE RESEARCH 11

2.1 Hypothesis for the research 11
2.2 Description of knowledge relating to the pathology in question 11
2.3 Summary of relevant pre-clinical experiments and clinical trials 12
2.4 Description of the population to be studied and justification for the choice of participants 12
2.5 Name and brief description of experimental medical device(s) 13
2.6 Description and explanation of how to use the device(s) and the duration of treatment 13
2.7 Summary of the known and foreseeable benefits and risks for the research participants 14

3 OBJECTIVES 15

3.1 Primary objective 15
3.2 Secondary objectives 15

4 RESEARCH DESIGN 15

4.1 Concise description of assessment criteria 15
4.2 Description of research methodology 16
4.3 Description of actions taken to reduce or avoid bias 17

5 PROCEDURE FOR THE RESEARCH 17

5.1 Screening period (V0; M-2 to D-1) 18
5.2 PVE period (V1; D0, D1 and D7) 19
5.3 Post-PVE visit (V2; Between Week 4 – Week 6): 21
5.4 Surgery (V3; Between Week 4 – Week 8): 21
5.5 Post-surgery visits (M1; M3; M6 after surgery): 22
5.6 End research visit (M12 after surgery): 23
5.7 Expected length of participation and description of the chronology and duration of the research. 25
5.8 Table or diagram summarising the chronology of the research 25
5.9 Distinction between care and research 27

6 ELIGIBILITY CRITERIA 27

6.1 Inclusion criteria 27
6.2 Non-inclusion criteria 28
6.3 Recruitment methods 28
6.4 Termination rules 29

7 MEDICAL DEVICE USED IN RESEARCH & TREATMENTS, PROCEDURES AND STRATEGIES ASSOCIATED WITH USING THE DEVICE 31

7.1 Experimental device being researched 31
7.2 Authorised and prohibited treatments (medicinal, non medicinal, surgical) including rescue medications 33
7.3 Description of the non-investigational treatment 33

8 ASSESSMENT OF PERFORMANCE OR EFFICACY 33

8.1 Description of parameters for assessing performance or efficacy 34
8.2 Anticipated methods and timetable for measuring, collecting and analysing the parameters for assessing performance or efficacy 34

Ce document est la propriété du DRCD / APHP. Toute reproduction est formellement interdite.

9 SPECIFIC RESEARCH COMMITTEES 34

9.1 Steering committee 34

10 SAFETY ASSESSMENT - RISKS AND RESTRICTIONS ADDED BY THE RESEARCH .. 35

10.1 Definitions 35
10.2 The investigator's roles 37
10.3 Specific features of the protocol 39
10.4 Procedures and deadlines for notifying the sponsor 42
10.5 Period for notifying the sponsor 44
10.6 The sponsor's roles 44
10.7 Data Safety Monitoring Board 48

11 DATA MANAGEMENT 50

11.1 Data collection methods 50
11.2 Right to access source data and documents 51
11.3 Data processing and storage of documents and data 52
11.4 Ownership of the data 54

12 STATISTICAL ASPECTS 54

12.1 Description of statistical methods to be used including the timetable for the planned interim analyses 54
12.2 Calculation hypotheses for the number of subjects required and the result 55
12.3 Method for taking into account missing, unused or invalid data 56
12.4 Management of modifications made to the analysis plan for the initial strategy 56

13 QUALITY CONTROL AND ASSURANCE 56

13.1 General organisation 56
13.2 Quality control 57
13.3 Case Report Form 58
13.4 Management of non-compliances 58
13.5 Audits / inspections 59
13.6 Primary investigator's commitment to assume responsibility 59

14 ETHICAL AND LEGAL CONSIDERATIONS 60

14.1 Methods for obtaining information and consent from research participants 60
14.2 Subject prohibited from participating in another research or an exclusion period anticipated after the research, if applicable 60
14.3 Compensation for subjects 60
14.4 Registration on the national register of subjects participating in biomedical research 61
14.5 Legal obligations 61
14.6 Request for an opinion from the Comité de Protection des Personnes (CPP, ethical review board) 61
14.7 Request for authorisation to ANSM 61
14.8 Commitment to compliance with the MR 001 "Méthodologie de Reference" 61
14.9 Modifications to the research 62
14.10 Final research report 62

15 FUNDING AND INSURANCE 62

15.1 Funding source 62
15.2 Insurance 62

16 PUBLICATION RULES 63

16.1 Mention of the affiliation of AP-HP for projects sponsored or managed by AP-HP 63
16.2 Mention of the AP-HP manager (DRCD) in the acknowledgements of the text 63
16.3 Mention of the financier in the acknowledgements of the text 63

17 BIBLIOGRAPHY 63

Ce document est la propriété du DRCD / APHP. Toute reproduction est formellement interdite.

Ce document est la propriété du DRCD / APHP. Toute reproduction est formellement interdite.

1 SUMMARY

Full title	Multicentric prospective study assessing the efficiency of preoperative reversible selective portal vein embolization in patients requiring major hepatic resection
Acronym	EMBORES
Coordinating Investigator	Ibrahim DAGHER<br>Department of minimally invasive digestive surgery<br>Antoine Béclère hospital, Clamart<br>Phone: + 33 1 45 37 45 45<br>Email: ibrahim.dagher@aphp.fr
Sponsor	Assistance Publique – Hôpitaux de Paris
Scientific justification	Portal vein embolization (PVE) is used before major hepatectomy to induce hypertrophy of the future liver remnant (FLR). A non-absorbable material is generally used for embolization, as it provides effective, permanent vascular occlusion. Our team has developed a minimally invasive technique of reversible PVE using gelfoam powder. The aim of this study is to assess the tolerance and efficiency of preoperative reversible selective PVE in patients requiring major hepatic resection.
Primary objective and assessment criterion	We hypothesize that preoperative reversible PVE allows to similarly increase FLR volume in comparison with classical non-reversible PVE in patients requiring major liver resection with limited FLR.<br>The hypertrophy ratio after classical non-reversible PVE is evaluated in literature as 13%. We estimated the hypertrophy ratio after reversible PVE as 9% in a previous study but this study was retrospective.<br>The aim of this pilot study is to estimate prospectively the hypertrophy ratio after reversible PVE.<br><br>Primary End Point: Hypertrophy ratio of FLR volume /

Ce document est la propriété du DRCD / APHP. Toute reproduction est formellement interdite.

	total liver volume between the baseline and after the PVE, evaluated by computed tomography scan volumetry 4-6 weeks after PVE.
Secondary objectives and assessment criteria	To compare the hypertrophy ratio after reversible PVE estimated in this study with the classical non-reversible PVE (13%).<br>To show benefits (related to PVE): Technical feasibility, Morbidity, Portal recanalization (partial and complete), Performing planned surgical procedure<br>To show benefits (related to hepatectomy): Per and postoperative morbidity<br>Secondary End Points:<br>Embolization: Percentage of completed PVE; Per and post procedure morbidity, Rate of portal recanalization, Rate of major liver procedures<br>Hepatectomy: Inflammation and adhesions during portal pedicles dissection, intra and postoperative morbidity
Experimental design	Single-arm feasibility study
Population involved	Adult patients requiring major hepatic resection
Inclusion criteria	- Patient aged between 18 and 80 years<br>- Patient requiring major liver resection (at least 3 segments)<br>- PVE indication decided in a multidisciplinary meeting<br>- Patients who are able to understand and follow instructions and who are able to participate in the study for the entire period<br>- Patients must have given their written informed consent to participate in the study after having received adequate previous information and prior to any study-specific procedures<br>- Patient affiliated to the French National Social

Ce document est la propriété du DRCD / APHP. Toute reproduction est formellement interdite.

	Security System
Non-inclusion criteria	- American Score of Anesthesiologist (ASA) ≥ 3<br>- Portal vein or hepatic vein thrombosis<br>- Associated (6 weeks before or after PVE) percutaneous liver treatment<br>- Patient not covered by social security service<br>- Patient under guardianship<br>- Participation in another clinical trial during the preceding 3 months.<br>- Patients with a medical disorder, condition, or history of such that would impair the patient's ability to participate or complete this study in the opinion of the investigator.<br>- Patients who have already had anaphylactic or anaphylactoid reactions during the injection of iodinated contrast medium (edema of Quincke…)<br>- Patients with an allergy to pork products<br>**During the hospitalization and before the PVE**<br>- Severe renal insufficiency (glomerular filtration rate ≤ 30mL/min eg calculated based on the Cockcroft formula).<br>- Pregnant women, or breast feeding women, or women with childbearing potential not using a combination of condoms and a safe and highly effective contraception method during the participation to the research (hormonal contraception with implants or oral contraceptives, or intrauterine devices).
Device undergoing research	Reversible PVE using Curaspon® (Absorbable Gelatin Hemostats), Cura Medical.
Other procedures added by the	NA

Ce document est la propriété du DRCD / APHP. Toute reproduction est formellement interdite.

research	
Risks added by the research	The risk added by the research is defined as D
Practical procedure	Percutaneous access to the portal vein is achieved under general anesthesia with ultrasound or fluoroscopic control. After assessment of portal venous anatomy, embolization of selected portal vein segments is performed using the powdered form of an absorbable gelatin.
Number of subjects chosen	50
Number of centers	National study involving 7 centers:
Research period	Duration of Recruitment: 18 months<br>Duration of participation of each patient: 18 months<br>Total duration of study: 36 months
Number of inclusions expected per center and per month	1 patient/ month / center
Statistical analysis	Descriptive analysis: characteristics of patients included will be summarized using frequency tables for categorical variables and mean, standard deviation etc. for continuous variables.<br>Primary analysis: based on full analysis set according to intent-to-treat (ITT) principle. The primary end point will be hypertrophy ratio of FLR volume / total liver volume between the baseline and after the PVE, evaluated by computed tomography scan volumetry 4-6 weeks after PVE. This ratio and the 95% confidence interval will be estimated. If necessary a per protocol analysis will be done.<br>Secondary analysis: The estimated ratio will be comparing to the classical ratio of 13% using a Student T test or a non-parametric Wilcoxon test.<br>All other secondary analysis will be analyzed descriptively with appropriate statistical methods.

Ce document est la propriété du DRCD / APHP. Toute reproduction est formellement interdite.

	Safety analysis: descriptive analysis.
Funding source	PHRC National 2015
Data Safety Monitoring Board anticipated	Yes

Ce document est la propriété du DRCD / APHP. Toute reproduction est formellement interdite.

2 SCIENTIFIC JUSTIFICATION FOR THE RESEARCH

2.1 Hypothesis for the research

Postoperative liver failure is the major cause of death following liver resection, directly related to the rate of remaining functional liver. The safe minimal size for future liver remnant (FLR) is approximately 25% for patients without significant underlying liver disease and 40% for patients with cirrhosis.

Portal vein embolization (PVE) is used before major hepatectomy, to induce hypertrophy of the FLR (1). Different embolic agents have been used for PVE. Since the embolized liver is most of the time resected, permanent embolization of the portal vein and its branches is frequently preferred. However, a theoretic risk of accidental definitive contralateral embolization is associated with non-reversible embolization, and the use of an absorbable agent could be safer.

A non-reversible material is generally used for PVE, as it provides effective, permanent vascular occlusion. However, a multi-stage management of hepatic malignancies is becoming increasingly common, and the use of an absorbable agent may increase the number of treatment combinations. Furthermore, reversible PVE might allow safer subsequent arterial chemo-embolization and may be more suitable for cirrhotic patients, as the associated functional decline would be transient. We can even imagine performing reversible PVE before living donor adult-to-adult liver transplantation.

During the last 10 years, our team has performed preliminary studies on the use of an absorbable agent for PVE (2, 3). Supported by these encouraging results, we decided to plan a multicentric single-arm prospective study assessing the efficiency of preoperative reversible selective portal vein embolization in patients requiring major hepatic resection. We hypothesize that the use of absorbable agent would be as efficient as the non-reversible in terms of increase of FLR volumes.

2.2 Description of knowledge relating to the pathology in question

Partial occlusion of the portal vein before major hepatectomy (resection of at least 3 liver segments) can be performed by percutaneous embolization or surgical ligation. In fact occlusion of one branch of the portal vein results in hemodynamic changes and the upregulation of various humoral mediators, leading to the atrophy of ipsilateral segments and hypertrophy of contralateral segments (4). One limitation of major hepatectomy is the liver

Ce document est la propriété du DRCD / APHP. Toute reproduction est formellement interdite.

volume remaining after liver resection or FLR, which should be sufficient to ensure its homeostatic function. If this residual volume is low, the risk of occurrence of serious postoperative complications increases and exposes the body to liver failure. The safe minimum size of the FLR is > 25% of the total functional liver for patients without significant underlying liver disease and > 40% of the total functional liver for patients with cirrhosis.

PVE can be achieved by percutaneous approach by injection of fluids (glues, sclerosing agents) or vaso-occlusive material (particles, coils, plug). In comparison to portal surgical ligation, percutaneous PVE reduces the risk of intra-procedure complications. Besides, PVE facilitates future major hepatectomy since portal pedicles are not previously dissected.

2.3 Summary of relevant pre-clinical experiments and clinical trials

Our team has developed a technique of reversible PVE using gelfoam powder before hepatocyte transplantation in order to improve engraftment in primates (2, 3). The recanalization of the embolized portal territory was complete after about 2 weeks. Reversible PVE allowed hypertrophy of 40% of the non-embolized liver (2). This hypertrophy was similar to the results that were previously reported with the use of a non-reversible material in human beings (5) and was stable one year after the procedure.

In a preliminary clinical study performed in Antoine Béclère hospital, 20 patients requiring major liver resection underwent reversible PVE. Reversible PVE resulted in sufficient hypertrophy of the non-embolized liver (29%), which allowed planning surgery in all patients (6). In this preliminary study, PVE was safely performed in 3 patients requiring an extensive embolization. During one procedure, a small leak of absorbable agent in the contralateral portal branch portal vein occurred. This incident did not have any clinical repercussions. The use of a non-reversible embolic agent in this patient could have led to a complete permanent portal vein occlusion. The harmless nature of reversible PVE might allow an increase of the number of patients that could receive extended selective PVE.

2.4 Description of the population to be studied and justification for the choice of participants

Ce document est la propriété du DRCD / APHP. Toute reproduction est formellement interdite.

Patients considered for extended hepatic resections (major hepatectomy: three or more segments) due to primary hepatic tumors or metastatic liver disease, in order to induce hypertrophy of the FLR before surgery.

2.5 Name and brief description of experimental medical device(s)

Embolization of selected portal vein segments is performed using the powdered form of an absorbable gelatin (Curaspon®; CuraMedical B.V, Assendelft, Netherlans). Gelatin powder is dissolved in a solution of 80% iodixanol (Visipaque® 270 mg/mL; GE Healthcare, Vélizy-Villacoublay, France) and 20% saline (final concentration of 0.08 g/mL) to suspend the particles of absorbable gelatin powder completely and to obtain a homogeneous mix visible during the embolization.

MD class: IIa
CE Marking: 0483

2.6 Description and explanation of how to use the device(s) and the duration of treatment

Percutaneous PVE is performed using an ipsilateral or contralateral approach. Percutaneous access to the portal vein is achieved using general anesthesia under ultrasound or fluoroscopic control. The portal branch through which vascular access is achieved is predefined during a multidisciplinary meeting. The left or right branch of the portal vein is punctured with a needle under ultrasound guidance, and a catheter is introduced in the main portal vein allowing direct portography. A microcatheter is pushed through the distal part of the segmental portal branch, and the embolization mixture is progressively injected until full obstruction of subsegmental and segmental portal branches. The microcatheter is progressively pulled back releasing the mixture into the sectorial branch. The embolization is considered complete when the embolization agent is visible in subsegmental, segmental, and sectorial branches without enhancement during the parenchymal phase. Approximately 4 g of absorbable gelatin powder is required to perform a right PVE. A final portogram using the catheter in the main portal vein is performed to ensure patency of the remaining portal branches. Duration of a typical PVE is 45 to 120 minutes. During the procedure, patients will be exposed x-ray (fluoroscopic control), so dose area product (DAP) estimations of irradiation

Ce document est la propriété du DRCD / APHP. Toute reproduction est formellement interdite.

for each patient will also be measured. PVE will be realized at each center by interventional radiologists and/or surgeons.

2.7 Summary of the known and foreseeable benefits and risks for the research participants

Gelfoam (strips or powder form), is routinely used for therapeutic arterial embolization in bleeding control for various indications (i.e. bone trauma, epistaxis, postpartum bleeding, renal vascular injuries, post-procedure bleeding in endovascular procedures etc.) (7-12). In addition, gelfoam is already used for PVE by several teams but in addition to other embolization agents (13). Finally, our team has previously reported the use of gelfoam powder for PVE in a preliminary retrospective analysis as stated above (6).

The main benefit expected for the patient is to decrease morbidity related to PVE. Indeed, a portal thrombosis extension in case of non-reversible PVE is a contraindication to surgery. The development of an alternative strategy will protect patients from this dramatic complication. Besides, the proof of the efficiency of reversible PVE will help develop other applications that could be associated with benefits for patients and public health:

- repetition of reversible PVE allowing to increase the size of FLR for patients with very small remnant liver
- safer subsequent arterial chemoembolization
- possibility to perform reversible PVE before adult to adult living donor transplantation

Besides, reversible PVE does not constitute theoretically a disadvantage or reduction of chances to undergo major liver surgery for patients. Indeed, in case of insufficient FLR regeneration, the possibility to perform a classical non-reversible PVE still exists.

In order to protect patients from any deleterious effect we have defined study-ending criteria:

After 10 procedures, the Safety Monitoring Board will stop the study in case of significant number of severe complications related to PVE as:

- portal thrombosis extension (symptomatic or not)
- allergenic reaction requiring intensive care unit management
- pulmonary embolism diagnosed within 15 days after PVE

Ce document est la propriété du DRCD / APHP. Toute reproduction est formellement interdite.

3 OBJECTIVES

3.1 Primary objective

We hypothesize that preoperative reversible PVE allows to similarly increase FLR volume in comparison with classical non-reversible PVE in patients requiring major liver resection with limited FLR.

The hypertrophy ratio after classical non-reversible PVE is evaluated in literature as 13%. We estimated the hypertrophy ratio after reversible PVE as 9% in a previous study but this study was retrospective.

The aim of this pilot study is to estimate prospectively the hypertrophy ratio after reversible PVE.

3.2 Secondary objectives

To compare the hypertrophy ratio after reversible PVE estimated in this study with the classical non-reversible PVE (13%).

To show benefits (related to PVE):

- Technical feasibility
- Morbidity
- Rate of portal partial and complete recanalization
- Performing planned surgical procedure

To show benefits (related to hepatectomy):

- Peroperative morbidity (portal pedicles dissection)
- Postoperative morbidity (liver insufficiency) up to 90 days after the surgery

4 RESEARCH DESIGN

4.1 Concise description of assessment criteria

4.1.1 Primary assessment criterion

Hypertrophy ratio of FLR volume / total liver volume between the baseline and after the PVE, evaluated by computed tomography scan volumetry 4-6 weeks after PVE.

Ce document est la propriété du DRCD / APHP. Toute reproduction est formellement interdite.

4.1.2 *Secondary assessment criteria*

<u>Embolization:</u>

- Technical feasibility: Percentage of completed, partially completed and undone PVE
- Per and post procedure (within 6 weeks) morbidity (portal thrombosis, pulmonary embolism and other complications)
- Liver tolerance (liver function tests at D0, D1 and D7)
- Rate of portal partial and complete recanalization (subsegmental, segmental and sectorial)
- Rate of patients considered after PVE for surgery in their centers and rate of patients that finally undergo surgery

<u>Hepatectomy:</u>

- Inflammation and adhesions during portal pedicles dissection: quantitative scale (14), please refer to Addendum 3
- Intraoperative morbidity up to 90 days after surgery (liver failure and other complications due to surgery)

4.2 Description of research methodology

4.2.1 *Experimental plan*

Single-arm prospective feasibility and safety study with an 18 months inclusion period and 18 months of follow-up period.

4.2.2 *Number of centres participating*

Seven regional centers are participating, including 5 university hospitals and 2 private non-profit public service hospitals.

- **Recruitment centres**

Surgery department will be considered as recruitment centres.

- **Non-recruitment centres**

Ce document est la propriété du DRCD / APHP. Toute reproduction est formellement interdite.

Reversible PVE procedure using Curaspon ® Powder, specific act of the research, will be performed in presence of interventional radiologists. Radiologist's centres will be considered as non-recruitment centres.

4.3 Description of actions taken to reduce or avoid bias

4.3.1 Identification of the subjects

For this research, the subjects will be identified as follows:

Centre No. (3 numerical positions) - Selection order No. of the person in the centre (4 numerical positions) - surname initial - first name initial

This reference is unique and will be retained for the entire research period.

4.3.2 Centralization of CT-scan volumetry

Results of CT-scan volumetry at baseline and at 4 to 6 weeks after PVE procedure will be centralized. Volumes will be assessed by one independent blind observer from anonymized CT-scan series (CD-rom). A standard operational procedure for the centralization of CT-scan volumetry will be drafted.

4.3.3 Training of operators

Before the first PVE, a senior operator will train different participants of the study. If necessary, at the first PVE, a senior interventional radiologist will oversee the procedure.

5 PROCEDURE FOR THE RESEARCH

Before any examination or act related to the research, the investigator collects the free and informed consent in writing of the person undergoing research

Subjects whose consent is sought	Who informs the subject and collects their consent	When is the subject informed	When is the subject's consent collected
Legal participants	Investigator (Digestive surgeon)	Screening period during the pre-embolization consultation	Screening period during the pre-embolization consultation or later during the hospitalization

Ce document est la propriété du DRCD / APHP. Toute reproduction est formellement interdite.

5.1 Screening period (V0; M-2 to D-1)

Screening period includes procedures performed in routine in order to plan PVE prior to a major liver resection:

Step 1: Abdominal CT-scan / CT-scan volumetry

Abdominal CT-scan will be performed in the radiology department of the center, up to 2 months before reversible PVE. Serial scanning will be performed with CT-scanner and images will be enhanced with an intravenous bolus injection of contrast agent (liver protocol: arterial, portal and venous phases).

Then, a CT-scan liver volumetry will be performed, as it always performed when planning to perform a PVE before major liver resection.

Step 2: Multidisciplinary meeting:

No specific pre-embolization investigations will be recommended except a CT-scan liver volumetry. Pre-existing liver function tests will be retrieved in medical records of patients. All consecutive eligible patients will be discussed at a weekly multidisciplinary meeting including surgeons, oncologists, radiologists and pathologists and the decision to perform pre-operative PVE will be taken on an individual basis as the decision to perform or not the subsequent liver surgery. The portal branches that will be occluded are defined during this multidisciplinary meeting.

Step 3: Pre-embolization consultation

Pre-embolization visit will be planned up to one day before reversible PVE.

During this consultation:

- Investigator will check eligibility criteria,
- All selected patients will receive an explanation of the study protocol,
- Written consent will be collected,

In a scenario where the pre-embolization consultation takes place before the 3 days preceding PVE, a prescription will be delivered in routine for β-hCG test and creatinine/estimated glomerular filtration rate (Cockcroft formula) in an external laboratory unit. Creatinine/estimated glomerular filtration rate and β-hCG test should be done within 3 days before reversible PVE. Patients will have to bring results the day of the PVE procedure.

Ce document est la propriété du DRCD / APHP. Toute reproduction est formellement interdite.

Step 4: Consultation with the anesthetist

Consultation with the anesthetist will be planned within one month before reversible PVE. ASA score will be determined and assessment of cardiac function will be done at anesthetist's discretion.

5.2 PVE period (V1; D0, D1 and D7)

Reversible PVE will be performed in a period of 2 months following pre-embolization abdominal CT-scan. PVE hospitalization will last 1-2 days. The day of the PVE, criteria eligibility will be checked. **If all criteria are fulfilled, the patient is included in the study and the written consent will be collected if it was not collected during the pre-embolization consultation. The investigator will complete the e-CRF in order to include the patient before the PVE**.

At D0, the following investigations will be performed:

- Medical examination (Weight, Height, Heart Rate, Arterial pressure)
- Complete blood count (Numération Formule Sanguine: Hématie, Hémoglobine, Hématocrites, VGM, CCMH, Leucocytes, Réticulocytes)
- Liver function tests (ASAT, ALAT, GGT, Alkaline Phosphatase, Total and Conjugated bilirubin)
- Prothrombin time
- Reversible PVE with Curaspon® Powder

Percutaneous access to the portal vein is achieved under general anesthesia with ultrasound or fluoroscopic control. Venous cartography of the liver is achieved with fluoroscopic control. All centers will use Visipaque ® 270 mg/mL; GE Healthcare, Vélizy-Villacoublay, France, as fluoroscopic agent. PVE technique remains unchanged but a mixture of Curaspon® is injected.

Patient's management during hospitalization will be globally performed at the discretion of each center.

- Monitor intraprocedure morbidity
- Adverse events

Ce document est la propriété du DRCD / APHP. Toute reproduction est formellement interdite.

Adverse events will be questioned and documented in the e-CRF. After discharge, patients will be handed a booklet describing adverse events that require emergency consultation (abdominal pain, fever…) and contact information for the digestive surgeon who is available on 24-hour call according to radiology society recommendations. All adverse events will be indexed in this booklet. At each visit, patients should bring their booklet.
Every patient will be given a personal protocol card with the name and the contact information of the main investigator to call in case of any adverse event (Addendum 7).

- Concomitant treatments

Concomitant treatments will be questioned and documented in the e-CRF.

At D1, the following investigations will be performed:

- Complete blood count (NFS: Hématie, Hémoglobine, Hématocrites, VGM, CCMH, Leucocytes, Réticulocytes)
- Liver function tests (ASAT, ALAT, GGT, Alkaline Phosphatase, Total and Conjugated bilirubin)
- Prothrombin time
- Monitor postoperative morbidity (portal thrombosis, pulmonary embolism and complications)
- Adverse events
- Concomitant treatments

At D7, the following investigations will be performed:

- Complete blood count (NFS: Hématie, Hémoglobine, Hématocrites, VGM, CCMH, Leucocytes, Réticulocytes)*
- Liver function tests (ASAT, ALAT, GGT, Alkaline Phosphatase, Total and Conjugated bilirubin)*
- Prothrombin time*
- Monitor postoperative morbidity (portal thrombosis, pulmonary embolism and complications)

Ce document est la propriété du DRCD / APHP. Toute reproduction est formellement interdite.

- Adverse events
- Concomitant treatments

* If the patient discharged, a prescription will be delivered, in routine, in order to make samples at D7 in an external laboratory unit.

5.3 Post-PVE visit (V2; Between Week 4 – Week 6):

Visit 2 will be performed between 4 to 6 weeks after percutaneous PVE procedure.

All patients will return to hospital for an ambulatory assessment in their respective center in order to monitor post-procedure morbidity.

At post-PVE visit, the following investigations will be performed:

- Medical examination (Height, Weight, Heart Rate, Palpation)
- Abdominal CT-scan / CT-scan volumetry

A CT-scan volumetry will be performed 4 to 6 weeks after PVE in the radiology department of the center. The CT-scan will be performed with contrast intravenous injection (liver protocol: arterial, portal and venous phases) as it is routinely performed after PVE.

- Monitor postoperative morbidity (portal thrombosis, pulmonary embolism and complications)
- Adverse events
- Concomitant treatments

5.4 Surgery (V3; Between Week 4 – Week 8):

Visit 3 will be usually performed between 4 to 8 weeks after percutaneous PVE procedure.

All patients who underwent PVE procedure with sufficiently hypertrophy of non-embolized liver segments will return to hospital in order to undergo liver surgery. Decision to proceed with surgery will be left on investigator's discretion.

The day of the surgery, at V3, the following investigations will be performed:

- Medical examination
- Standard laboratory blood sampling (complete blood count, liver function tests, prothrombin time).

Ce document est la propriété du DRCD / APHP. Toute reproduction est formellement interdite.

- Liver surgery
- Assessment of the level of inflammation and adhesions during portal pedicles dissection

The senior surgeon will evaluate the level of inflammation and adhesions during portal pedicles dissection using a quantitative scale (Addendum 3)

- Monitor intraoperative morbidity.
- Adverse events

Postoperative complications will be classified as specific liver-related complications (cirrhotic decompensation, hemorrhage, biliary collection) and general complications. Complication severity will be stratified according to the modified Clavien classification (16).

- Concomitant treatments

At D5 after the surgery, the following investigations will be performed as usual:

- Medical examinations
- Standard laboratory blood sampling
- Monitor postoperative liver function (liver function tests including total bilirubin and Prothrombin time)

Liver failure will be defined using the "50-50 criterion" (prothrombin time < 50%, total bilirubin > 50 µm/L) (15).

Ascites will be defined as follows: > 500mL/day postoperative drainage or percutaneous puncture.

- Concomitant treatments
- Adverse events

5.5 Post-surgery visits (M1; M3; M6 after surgery):

All patients who underwent liver surgery will return to hospital (if not still hospitalized) for an ambulatory assessment in their respective center at a systematic follow-up visit one month (M1 ± 1 week), three months (M3 ± 2 weeks), and 6 months (M6 ± 2 weeks).

At each visit after the surgery, the following investigations will be performed as usual:

- Medical examinations

Ce document est la propriété du DRCD / APHP. Toute reproduction est formellement interdite.

- Complete blood count*
- Liver function tests*
- Prothrombin time*
- Monitor postoperative morbidity
- Concomitant treatments
- Adverse events

* at M3 and M6, blood test will be done if necessary

5.6 End research visit (M12 after surgery):

For patients who undergo liver surgery, a safety follow-up visit will be planned 12 months after the liver surgery.

For patients who did not undergo liver surgery, a safety follow-up visit will be planned 12 months after the reversible PVE.

In case of prematurely termination of research, this safety follow-up should be realized.

At end research visit, the following investigations will be performed:

- Medical examinations
- Complete blood count*
- Liver function tests*
- Prothrombin time*
- Monitor postoperative morbidity
- Concomitant treatments
- Adverse events

* Blood tests will be done if necessary

Ce document est la propriété du DRCD / APHP. Toute reproduction est formellement interdite.

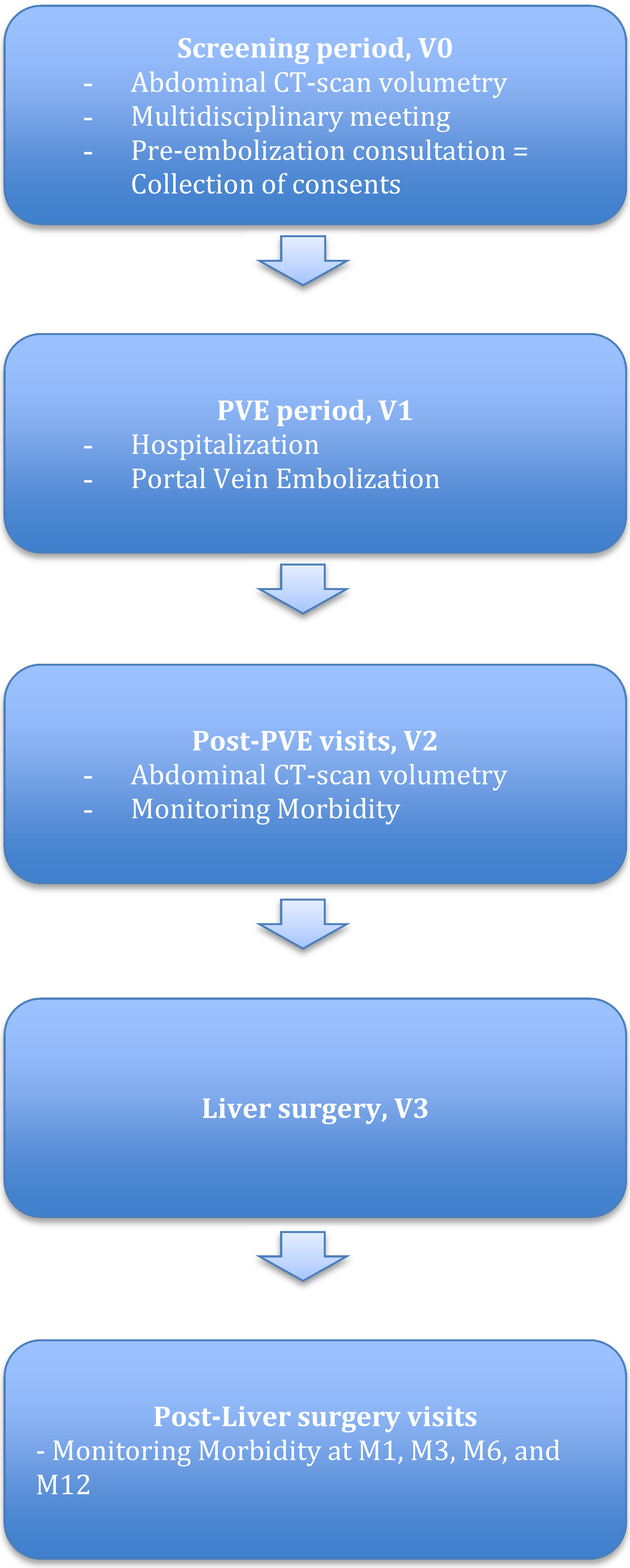

Ce document est la propriété du DRCD / APHP. Toute reproduction est formellement interdite.

5.7 Expected length of participation and description of the chronology and duration of the research.

Inclusion period	18 months
The included subjects' length of participation, of which:	
Maximum period between selection and reversible PVE	2 months
Maximum period between reversible PVE and surgery	2 months
Follow-up period:	14 months
Exclusion period	1 month
Total duration of study	36 months

5.8 Table or diagram summarising the chronology of the research

Visit	*Screening period (V0)*	*PVE period (V1)*			*Post-PVE visit (V2)*	*Surgery period (V3)*	*Post-surgery visits*				*End research visit*
Week	*D-60 to D-1*	*D0*	*D1*	*D7*	*Between W4-W6*	*BetweenW4-W8*	*D5 after surgery*	*M1 after surgery*	*M3 after surgery*	*M6 after surgery*	*M12 after surgery*[1]
Informed consent	*X*										
Serum β-hCG	*X*[2,3]										
Creatinine / estimated glomerular filtration rate (Cockcroft formula)	*X*										
Abdominal CT- scan	*X*[4]				*X*[5]						
CT-scan liver volumetry	*X*[4]				*X*[5]						
Complete blood count (NFS: Hématie, Hémoglobine, Hématocrites, VGM, CCMH, Leucocytes, Réticulocytes)		*X*	*X*	*X*		*X*	*X*	*X*	*X**	*X**	*X**

Liver function tests (ASAT, ALAT, GGT, Alkaline phosphatase, Total and Conjugated bilirubin)		*X*	*X*	*X*		*X*	*X*	*X*	*X**	*X**	*X**
Prothrombin time		*X*	*X*	*X*		*X*	*X*	*X*	*X**	*X**	*X**
Medical examination (Weight, Height, HR, Arterial pressure)		*X*			*X*	*X*	*X*	*X*	*X*	*X*	*X*
Reversible Portal Vein Embolization with Curaspon®		<u>*X*</u>									
Intraprocedure morbidity monitoring (PVE)		*X*									
Postprocedure morbidity monitoring (PVE)			*X*	*X*	*X*						
Liver surgery						*X*					
Inflammation and Adhesion Scale						*X*					
Intraoperative morbidity monitoring (Hepatectomy)						*X*					
Post-operative morbidity monitoring (Hepatectomy)							*X*	*X*	*X*	*X*	*X*
Concomitant Medication	*X*	*X*	*X*	*X*	*X*	*X*	*X*	*X*	*X*	*X*	*X*
Adverse event reporting	*X*	*X*	*X*	*X*	*X*	*X*	*X*	*X*	*X*	*X*	*X*

[1] For patients who undergo liver surgery, a safety follow-up visit will be planned 12 months after liver surgery. For patients who did not undergo liver surgery, a safety follow-up visit will be planned 12 months after reversible PVE.
[2] Only for women with childbearing potential.
[3] β-hCG test and Creatinine / estimated glomerular filtration rate should be done within 3 days before reversible PVE.
[4] CT-scan liver volumetry will be done no later than up to 2 months before inclusion visit.
[5] CT-Scan liver volumetry will be realized to 4-6 weeks after the PVE procedure
X* Blood test will be done if necessary according to investigator's discretion
X Procedures and treatments added because of the research

5.9 Distinction between care and research

**Procedures and treatments carried out as part of the research**	**Procedures and treatments associated with care**	**Procedures and treatments added because of the research**
Para-clinical exam (liver function tests)	*X*	
Reversible PVE using Curaspon®		*X*
Abdominal CT-scan / CT-scan volumetry	*X*	
Liver surgery	*X*	
Follow-up visits	*X*	

6 ELIGIBILITY CRITERIA

6.1 Inclusion criteria

- Patient aged between 18 and 80 years
- Patient requiring major liver resection (at least 3 segments)
- PVE indication decided in a multidisciplinary meeting
- Patients who are able to understand and follow instructions and who are able to participate in the study for the entire period
- Patients must have given their written informed consent to participate in the study after having received adequate previous information and prior to any study-specific procedures

Ce document est la propriété du DRCD / APHP. Toute reproduction est formellement interdite.

- Patient affiliated to the French National Social Security System

6.2 Non-inclusion criteria

- American Score of Anesthesiologist (ASA) ≥ 3
- Portal vein or hepatic vein thrombosis
- Associated (6 weeks before or after PVE) percutaneous liver treatment
- Patient not covered by social security service
- Patient under guardianship
- Participation in another clinical trial during the preceding 3 months.
- Patients with a medical disorder, condition, or history of such that would impair the patient's ability to participate or complete this study in the opinion of the investigator.
- Patients who have already had anaphylactic or anaphylactoid reactions during the injection of iodinated contrast medium (edema of Quincke…)
- Patients with an allergy to pork products

During the hospitalization and before the PVE

- Severe renal insufficiency (glomerular filtration rate ≤ 30mL/min eg calculated based on the Cockcroft formula).
- Pregnant women, or breast feeding women, or women with childbearing potential not using a combination of condoms and a safe and highly effective contraception method (hormonal contraception with implants or oral contraceptives, or intrauterine devices).

Intravenous contrast allergy was not considered as a non-inclusion criterion. However, patients with previous reaction to contrast agents will receive premedication before contrast intravenous injection as usual.

6.3 Recruitment methods

	Number of subjects
Sample size	*50*

Ce document est la propriété du DRCD / APHP. Toute reproduction est formellement interdite.

Number of centers	*7*
Inclusion period (months)	*18*
Number of subjects/centre	*8*
Number of subjects/centre/month	*1*

6.4 Termination rules

6.4.1 Criteria and methods for premature termination of research

Any subject can withdraw from participating in the research at any time and for any reason.

If a subject leaves the research prematurely, data relating to the subject can be used unless an objection was recorded when the subject signed the consent form.

In this case, the investigator must:

- Document the reason(s) in the medical file and the case report form:
  - Explicit withdrawal of consent

- Collect the assessment criteria when participation in the research ends, if the subject agrees

6.4.2 Criteria and methods for the premature termination of research procedure

- The investigator can permanently end the procedure of the research of a subject's for any reason that affects the subject's safety or which would be in the subject's best interests.
- In case of patient instability due to anesthesia
- In case of important bleeding leading to hemodynamic instability and life-threatening complication requiring critical care management or causing death (Grade IV-V)
- In case of portal vein access impossibility
- In case of impossibility to perform a selective portal vein embolization

Ce document est la propriété du DRCD / APHP. Toute reproduction est formellement interdite.

In case of a premature termination of the research procedure, the subject is still included in the study and must continue the follow-up visit of the research, until the end of his participation in order to assemble data of evaluation criteria. The investigator must document the reason.
For patients unable to undergo PVE, management will be performed according to local practices.

The case report form must list the various reasons for premature termination of the research procedure:

- ☐ Ineffective
- ☐ Adverse reaction
- ☐ Other medical problem
- ☐ Subject's personal reasons

6.4.3 Methods for replacing subjects, if applicable

No subjects will be replaced.

6.4.4 Terminating part or all of the research

AP-HP as sponsor or the Competent Authority (ANSM) can prematurely terminate all or part of the research, temporarily or permanently, upon the recommendation of a data and safety monitoring board in the following situations:

- first of all, if suspected unexpected serious adverse reactions (SUSARs) are observed in the patients participating from the study.
- likewise, unexpected facts, new information about the product, in light of which the objectives of the research or of the clinical programme are unlikely to be achieved, can lead AP-HP as sponsor or the Competent Authority (ANSM) to prematurely halt the research.
- AP-HP as sponsor reserves the right to permanently suspend inclusions at any time if it appears that the inclusion objectives are not met.

If the research is terminated prematurely, the decision and justification will be given by the sponsor, AP-HP, to the Competent Authority (ANSM) and to the CPP within 15 days, along with recommendations from the Data and Safety Monitoring Board.

Ce document est la propriété du DRCD / APHP. Toute reproduction est formellement interdite.

7 MEDICAL DEVICE USED IN RESEARCH & TREATMENTS, PROCEDURES AND STRATEGIES ASSOCIATED WITH USING THE DEVICE

7.1 Experimental device being researched

7.1.1 Device identification

CURASPON® POWDER is an Absorbable Gelatin Powder Hemostat. Curaspon® is developed by CuraMedical B.V (Assendelft, The Netherlands).

7.1.2 Intended purpose of device

During reversible selective portal vein embolization in patients requiring major hepatic resection, Curaspon is used as a reversible agent to block blood flow in selected portal branches. The aim is to atrophy the resected liver and induce a hypertrophy of the contralateral liver with a reversible agent.

Gelfoam (strips or powder form), is routinely used for therapeutic arterial embolization in bleeding control for various indications (i.e. bone trauma, epistaxis, postpartum bleeding, etc.) (7-12). In addition, gelfoam is already used for PVE by several teams but in addition to other embolization agents (13). In 2008, our team has developed a technique of reversible PVE using gelfoam powder, before hepatocyte transplantation in order to improve engraftment in primates (2,3). The reversible PVE allowed a hypertrophy of 40% of the non-embolized liver (2). This hypertrophy was similar to the results that were previously reported with the use of a non-reversible material in human beings (4) and was stable one year after the procedure.
In a preliminary clinical study we performed in Antoine Béclère hospital 20 reversible PVE in patients requiring major liver resection. Reversible PVE resulted in a sufficient hypertrophy of the non-embolized liver (29%), which allowed planning surgery in all patients (6). In this preliminary study, PVE was safely performed in 3 patients requiring an extensive embolization.

7.1.3 How to use the device

Ce document est la propriété du DRCD / APHP. Toute reproduction est formellement interdite.

Percutaneous PVE is performed using an ipsilateral or contralateral approach. Percutaneous access to the portal vein is achieved using general anesthesia under ultrasound or fluoroscopic control. The portal branch through which vascular access is achieved is predefined during a multidisciplinary meeting. The left or right branch of the portal vein is punctured with a needle under ultrasound guidance, and a catheter is introduced in the main portal vein allowing direct portography. A microcatheter is pushed through the distal part of the segmental portal branch, and the embolization mixture is progressively injected until full obstruction of subsegmental and segmental portal branches. The microcatheter is progressively pulled back releasing the mixture into the sectorial branch.

Embolization of selected portal vein segments is performed using the powdered form of an absorbable gelatin powder (Curaspon®; CuraMedical B.V, Assendelft, The Netherlands).. Gelatin powder is dissolved in a solution of 80% iodixanol (Visipaque® 270 mg/mL; GE Healthcare, Vélizy-Villacoublay, France) and 20% saline - NaCl 0.9% (final concentration of 0.08 g/mL) to suspend the particles of absorbable gelatin powder completely and to obtain a homogeneous mix visible during the embolization. The mixture will be realized by interventional radiologists and/or surgeons in operating room. The embolization is considered complete when the embolization agent is visible in subsegmental, segmental, and sectorial branches without enhancement during the parenchymal phase. Approximately 4 g of absorbable gelatin powder is required to perform a right PVE. A final portogram using the catheter in the main portal vein is performed to ensure patency of the remaining portal branches. Duration of a typical PVE is 45 to 120 minutes. During the procedure, patients will be exposed x-ray (fluoroscopic control), so dose area product (DAP) estimations of irradiation for each patient will also be measured. PVE will be realized at each center by interventional radiologists and/or surgeons.

7.1.4 Summary of necessary training and experience for device usage

A training session will be conducted by an experienced radiologist for the preparation and use of CURASPON® during a PVE. A demonstrating video will be also showed before and during the opening of centers. If necessary, all the participants will have the possibility to attend a PVE with Curaspon® at Antoine Béclère hospital, the leading centre.

The embolization procedure will be performed as usual. Thus, no specific learning curve will be necessary.

Ce document est la propriété du DRCD / APHP. Toute reproduction est formellement interdite.

Before the first PVE, a senior operator will train different participants of the study. If necessary, at the first PVE, a senior interventional radiologist will oversee the procedure.

7.2 Authorised and prohibited treatments (medicinal, non medicinal, surgical) including rescue medications

Anticoagulants are forbidden. The treatment should be stopped at least 24 hours before the embolization.

7.3 Description of the non-investigational treatment

- Iodixanol (Visipaque®), 270 mg mg/mL, injection solution (RCP ANSM du 03/07/2015) to dissolved gelatin powder (17).
- Sodium Chloride 0.9% BIOPERF, solution for perfusion (RCP ANSM du 26/06/2015) to dissolved gelatin powder (18).
- In case of previous allergic reaction to contrast medium, the following protocol can be used :
  - 1 tablet Atarax 100 mg (RCP ANSM du 09/12/2013), the day before the exam, at bedtime (19)
  - 1 tablet Atarax 100 mg, 2 hours before the exam
  - 1 tablet Polaramine repetabs (RCP ANSM du 13/10/2011), morning and evening, 48 hours before the exam for hyper-allergic patients (20)

However premedication can also be performed according to habits of each center, after validation by the radiologist investigator.

8 ASSESSMENT OF PERFORMANCE OR EFFICACY

The percentage of FLR volume / total liver volume will be evaluated by CT scan volumetry 4 to 6 weeks after PVE. All abdominal CT scan will be performed in the radiology department of the center and with contrast intravenous injection (liver protocol: arterial, portal and venous phases).

Volumes will be assessed by one independent blind observer using Advance 4D volumetric software (GE Healthcare, Little Chalfont, United Kingdom), from anonymized CT

Ce document est la propriété du DRCD / APHP. Toute reproduction est formellement interdite.

scan series (compact disc). Total, non-embolized (FLR) volume and embolized liver volume (ELV) will be measured.

The following formulas will be used to calculate percentage of FLR volume:

%FLR volume prePVE = (FLR prePVE volume / (total liver prePVE volume – tumor volume prePVE)) x 100

%FLR volume postPVE = (FLR postPVE volume / (total liver postPVE volume – tumor volume postPVE)) x 100

The following formulas will be used to calculate hypertrophy ratio:

%FLR volume postPVE - %FLR volume prePVE

8.1 Description of parameters for assessing performance or efficacy

See section 4.1

8.2 Anticipated methods and timetable for measuring, collecting and analysing the parameters for assessing performance or efficacy

See section 5.7

9 SPECIFIC RESEARCH COMMITTEES

9.1 Steering committee

Members of the committee: *Pr Ibrahim DAGHER, Chair (Antoine Beclère Hospital), Dr Hadrien TRANCHART (Surgeon), Dr Panagiotis LAINAS (Surgeon), URC responsible ; URC project referent ; URC biostatistician ; Clinical Research Associate and DRCD project referent.*

Missions:

- Defining the objective, compiling the protocol, and proposing protocol amendments during the study.
- Defining the general organization of the study, coordinating the information, initially determining the methodology, and monitoring implementation of the study.

Ce document est la propriété du DRCD / APHP. Toute reproduction est formellement interdite.

- Proposing management during the study taking into account the recommendations of the data safety monitoring board (DSMB) (refer to section 10.7). The sponsor, DRCD, will remain the decision-maker.

Operating methods: Every 6 months, the steering committee will be consulted to evaluate inclusion rate, and patient's safety**.**

10 SAFETY ASSESSMENT - RISKS AND RESTRICTIONS ADDED BY THE RESEARCH

10.1 Définitions

According to Article R1123-46 of the French Public Health Code :

- **Adverse event**

Any untoward medical occurrence in a trial subject, which does not necessarily have a causal relationship with the clinical trial or with the investigational product.

- **Adverse reaction of medical device or *In Vitro* Diagnostic Medical Device (IVD MD)**

Any untoward and adverse reaction to a medical device, or any incident that could have caused such a reaction if appropriate action had not been taken, in a trial subject or in a medical device user or any reaction related to a malfunction or deterioration in an *in vitro* diagnostic medical device which is harmful for the health of a trial subject.

- **Serious adverse event or reaction**

Any adverse event or reaction that at any dose of medication, results in death, threatens the life of the trial subject,, requires hospitalisation or prolongs existing hospitalisation, causes a severe or long-term disability or handicap, or results in a congenital abnormality or deformity.

- **Unexpected adverse reaction**

Any adverse reaction, of which the nature, severity or outcome is not consistent with the information related to the products, interventions and procedures used in the context of the clinical trial.

.

According to Article R5215-15 of the French Public Health Code:

- **Incident**

Ce document est la propriété du DRCD / APHP. Toute reproduction est formellement interdite.

- An untoward and adverse reaction occurring when using a medical device as intended;
- An untoward and adverse reaction occurring when a medical device is used not in accordance with the manufacturer's instructions;
- Any malfunction or alteration in the features or performance of a medical device;
- Any erroneous indication, omission and inadequacy in the labelling (instruction manual, user manual or maintenance manual).

According to Article R. 1123-46 of the Code de la Santé Publique and the guidelines for sponsors of clinical trials conducted on medical devices and *in vitro* diagnostic medical devices (ANSM)

- **New safety issue**

Any data which could significantly lead to a re-evaluation of the benefit-risk ratio of a MD or IVD MD under investigation or which could result in modifications concerning the use of the MD or IVD MD or the study procedures, such as for example:
a) any clinically significant increase in the frequency of occurrence of a serious expected adverse reaction;
b) suspected unexpected serious adverse reactions which have occurred in trial subjects who have terminated their participation in the trial and which are notified by the investigator to the sponsor together with follow-up reports;
c) any new safety issue relating to the conduct of the clinical trial or the use of the investigational MD or IVD MD, that may impact the safety of the trial subjects.

Examples:

- a serious adverse event likely to be related to the investigations and the diagnostic procedures of the trial and which could modify the conduct of the trial,
- a significant risk for the trial subjects such as for example a lack of effectiveness of the MD or IVD MD used in the treatment of a life threatening disease,
- significant safety results from a completed pre-clinical study which may impact the benefit-risk assessment (such as a biomechanical study),
- the premature termination, or temporary suspension for safety reasons, of a trial conducted on the same investigational medical device in another country,
- an unexpected serious adverse reaction associated with a non-investigational health product required for carrying out the trial (e.g. challenge agents, emergency treatment).

d) recommendations of the independent Data and Safety Monitoring Board (DSMB), if necessary, if they are relevant to public health;
e) any suspected unexpected serious adverse reaction transmitted to the sponsor by another sponsor of another clinical trial involving the same MD or IVD MD in another country.

Ce document est la propriété du DRCD / APHP. Toute reproduction est formellement interdite.

10.2 The investigator's roles

The investigator must **assess the seriousness criteria of each adverse event** and record all serious and non-serious adverse events in the case report form (eCRF).

The investigator must **document** serious adverse events **as thoroughly as possible** and provide a definitive medical diagnosis, if possible.

The investigator assesses the severity of the adverse events by using more general terms: according to the Dindo-Clavien classification of surgical complications.

TABLE : Dindo-Clavien classification of surgical complications.

Grades	Definition
Grade I	Any deviation from the normal postoperative course without the need for pharmacological treatment or surgical, endoscopic and radiological interventions.
	Allowed therapeutic regimens are: drugs as antiemetics, antipyretics, analgetics, diuretics and electrolytes and physiotherapy. This grade also includes wound infections opened at the bedside.
Grade II	Requiring pharmacological treatment with drugs other than such allowed for grade I complications.<br>Blood transfusions and total parenteral nutrition are also included.
Grade III	Requiring surgical, endoscopic or radiological intervention
Grade IIIa	Intervention not under general anesthesia
Grade IIIb	Intervention under general anesthesia
Grade IV	Life-threatening complication requiring intensive care management
Grade IVa	Single organ dysfunction (including dialysis)
Grade IVb	Multi organ dysfunction
Grade V	Death of a patient

The investigator assesses the causal relationship between the serious adverse events and the experimental medical device for a reversible PVE, its implementation action, the procedure

Ce document est la propriété du DRCD / APHP. Toute reproduction est formellement interdite.

added by the research (non-experimental treatment required for carrying out the research: Visipaque®, Atarax® and Polaramine repetabs®).
The method used by the investigator, based on the WHO method (WHO Uppsala Monitoring Centre), is based on the four following terms of causality:

- Certain
- Probable/likely
- Possible
- Unlikely

Their definitions are in the below table (extract from WHO-UMC causality categories, 17/04/2012).

TABLE: WHO-UMC causality categories (extrait)

**Causality term**	Assessment criteria*
**Certain**	- Event or laboratory test abnormality, with plausible time relationship to drug intake**<br>- Cannot be explained by disease or other drugs<br>- Response to withdrawal plausible (pharmacologically, pathologically)<br>- Event definitive pharmacologically or phenomenologically (i.e. an objective and specific medical disorder or a recognized pharmacological phenomenon)<br>- Rechallenge satisfactory, if necessary
**Probable / Likely**	- Event or laboratory test abnormality, with reasonable time relationship to drug intake**<br>- Unlikely to be attributed to disease or other drugs<br>- Response to withdrawal clinically reasonable<br>- Rechallenge not required
**Possible**	- Event or laboratory test abnormality, with reasonable time relationship to drug intake**<br>- Could also be explained by disease or other drugs<br>- Information on drug withdrawal may be lacking or unclear
**Unlikely**	- Event or laboratory test abnormality, with a time to drug intake**<br>- that makes a relationship improbable (but not impossible)

Ce document est la propriété du DRCD / APHP. Toute reproduction est formellement interdite.

	- Disease or other drugs provide plausible explanations

*All points should be reasonably complied with
*** Or study procedures*

The investigator must notify the sponsor without delay on the day when the investigator becomes aware of any incident. He must do so by completing the incident or incident risk report form (see appendix 5) and faxing it without delay to DRCI Safety Department (AP-HP) at +33 (0)1 44 84 17 99.

10.3 Specific features of the protocol

10.3.1 Serious adverse events that do not require the investigator to immediately notify the sponsor

These serious adverse events are only recorded in the "adverse event" section of the electronic case report form.

- **<u>Adverse events due to the portal vein embolization:</u>**

Intraprocedure adverse events:

- Anesthesia related complications (Grade I-II-III)
- Important bleeding leading to hemodynamic instability (Grade I-II-III)
- Biliary duct puncture (Grade I-II-III)

Postprocedure adverse events:

- Hemoperitoneum (Grade I-II-III)
- Liver abscess (Grade I-II-III)
- Puncture site abscess (Grade I-II-III)
- Ascites (> 500mL/day postoperative drainage or percutaneous puncture) (Grade I-II-III)
- Fever (Grade I-II-III)
- Liver hematoma depicted on CT scanner performed when necessary (Grade I-II-III).
- Symptomatic biliary leak (Grade I-II-III)

- **<u>Adverse events due to the hepatectomy</u>**

Intraoperative adverse events:

- Anesthesia related complications (Grade I-II-III)
- Bleeding (Grade I-II-III)

Ce document est la propriété du DRCD / APHP. Toute reproduction est formellement interdite.

- Extra hepatic visceral lesion (Grade I-II-III)
- Failure to perform the procedure (tumoral progression or perioperative difficulties) (Grade I-II-III)

Postoperative adverse events:

Specific morbidity:

- Biliary collection (Grade I-II-III)

General morbidity:

- Pulmonary (pleural effusion, atelectasia, pneumonia, pulmonary embolism etc.) (Grade I-II-III)
- Parietal (postoperative hernia, abdominal wall hematoma or abcess, etc.) (Grade I-II-III)
- Urinary (infection) (Grade I-II-III)
- Cardiovascular (cardiac failure, myocardial infarction, phlebitis) (Grade I-II-III)
- Postoperative functional ileus (Grade I-II-III)
- Gastroparesis (Grade I-II-III)
- Central or peripheral venous catheter infection (Grade I-II-III)

The occurrence of serious adverse events listed above when Grade ≥ 4 will be immediately notified to sponsor.

- **Normal and natural evolution of the pathology:**
    - Planned hospitalization for the protocol (CT scan, PVE, liver surgery)
    - planned hospitalization concerning the tumoral disease or the underlying liver disease
    - hospitalization for routine treatment or surveillance concerning the tumoral disease or the underlying liver disease (not related to a worsening of health)
    - worsening of the tumoral disease or the underlying liver disease

- **Special circumstances**
    - hospitalization of a pre-existent disease
    - hospitalization for medical or surgical treatment planned before the study

Ce document est la propriété du DRCD / APHP. Toute reproduction est formellement interdite.

- hospitalization for administrative or social reasons
- emergency room visit (<12 hours)

- **<u>Adverse events likely to be associated with the treatments prescribed as part of the patient's care during the monitoring of the research</u>**

10.3.2 Serious adverse events that require the investigator to immediately notify the sponsor

The investigator must report all adverse events that meet one of the seriousness criteria below, except for events listed in section 10.3.1 as not requiring immediate notification to the sponsor:<br>1- Death<br>2- Life threatening situation<br>3- Requiring hospitalisation or prolonging hospitalisation<br>4- Persistent or significant disability or incapacity<br>5- Congenital abnormality or birth defect<br>6- Or any other adverse event considered "medically significant".

The investigator will specify if the serious adverse event follows an incident related to the experimental medical device.

Other events requiring immediate notification to the sponsor:

- The serious adverse events associated with specific research procedures or exams, and which are expected, are:
  - Pulmonary embolism diagnosed within 15 days after PVE
  - Severe allergenic reaction to the embolizing agent (immediate reaction between 5 to 60 min)
  - Portal thrombosis extension (symptomatic or not) within 2 weeks

- For serious adverse events due to portal vein embolization:
  - Liver insufficiency (liver function tests) within a week
  - Peritonitis within 72 hours

Ce document est la propriété du DRCD / APHP. Toute reproduction est formellement interdite.

- Hemoperitoneum within 48 hours

- For serious adverse events due to hepatectomy
  - Cirrhotic decompensation within a week
  - Important bleeding necessitating transfusion within 48 hours

- Pregnancy

Any pregnancy occurring during the clinical trial, even if not associated with an adverse event must be reported to the sponsor by the investigator as soon as he/she has knowledge of.

- Serious adverse events listed in section 10.3.1 when Grade ≥ 4 will be immediately notified to sponsor.

10.4 Procedures and deadlines for notifying the sponsor

10.4.1 Serious adverse events

Notification of an SAE must initially be provided in a written report using the special form for reporting SAE (see Addendum 5). The report must be signed by the investigator.

Each item in the form must be completed by the investigator so that the sponsor can carry out the appropriate analysis.

The initial report to the sponsor must be rapidly followed up by one or more additional written reports describing the course of the event and any complementary information.

Whenever possible, the investigator will provide the sponsor with any documents that may be useful (medical reports, laboratory test results, results of additional exams, etc.). These documents must be made anonymous. In addition, the documents must include the following: research acronym, number and initials of the subject, nature and date of the serious adverse event.

Ce document est la propriété du DRCD / APHP. Toute reproduction est formellement interdite.

Any adverse event will be monitored until fully resolved (stabilization at a level considered acceptable by the investigator, or return to the previous state) even if the subject has left the trial.

The initial notification, the SAE follow-up reports and all other documents must be sent to the sponsor via fax only to the Vigilance Division of the DRCD, fax No. **01 44 84 17 99**.

For studies using e-CRF:
- the investigator completes the SAE notification form in the e-CRF, validates, prints and signs the form before sending it *via* fax.
- if it is not possible to connect to the e-CRF, the investigator will complete, sign and send the SAE notification form found in Addendum 5. As soon as the connection is restored, the SAE notification form in the e-CRF must be duly completed.

The investigator must comply with all requests from the sponsor for additional information.
For all questions relating to the notification of an adverse event, the Vigilance Division of the DRCD can be contacted via email: vigilance.drc@aphp.fr

10.4.2 Pregnancy discovery

The investigator completes the "form for monitoring a pregnancy that developed during a biomedical research", found in Addendum 6 and sends it by fax to the Vigilance Division at **01 44 84 17 99**.

The investigator must monitor the pregnant woman throughout her pregnancy or until the pregnancy is terminated, and must notify the sponsor of the outcome of the pregnancy, using this form.

If the outcome of the pregnancy falls within the definition of a serious adverse event (miscarriage, pregnancy termination, foetal death, congenital abnormality, etc.), the investigator must follow the procedure for reporting SAE.

Ce document est la propriété du DRCD / APHP. Toute reproduction est formellement interdite.

The initial pregnancy notification, the SAE follow-up reports and all other documents must be sent to the sponsor via fax only to the Vigilance Division - of the DRCD, fax No. **01 44 84 17 99**.

10.5 Period for notifying the sponsor

The investigator must notify the sponsor without delay of all SAE that occur in research subjects:

- after the embolisation
- throughout the period during which the participant is monitored,
- with no time limit, if the SAE is likely to be due to the medical device or to the research procedures

10.6 The sponsor's roles

The sponsor, represented by its Vigilance Division, continuously assesses the safety of each medical device throughout the research.

10.6.1 Analysis and declaration of serious adverse events

The sponsor assesses:

- the seriousness of all adverse events reported
- the causal relationship of these events with each experimental medical device and /or its implementation action and/or specific medical procedures/exams added by the research and with other possible treatments,
- the expected or unexpected nature of these adverse reactions.

All serious adverse events which the investigator and/or the sponsor believe could reasonably have a causal relationship with the experimental medical device are considered as suspected adverse reactions.

Reference safety information

Ce document est la propriété du DRCD / APHP. Toute reproduction est formellement interdite.

The assessment of the expectedness of serious adverse events/reactions by the sponsor is based on :

- For serious adverse events related to the experimental medical device and which are expected, are:
  - It is necessary to refer to the instructions or operating manual of Curaspon® Powder found in Addendum 4.
  - Pulmonary embolism diagnosed within 15 days after PVE
  - Severe allergenic reaction to the embolizing agent (immediate reaction between 5 to 60 min)
  - Portal thrombosis extension (symptomatic or not) within 2 weeks

- For serious adverse events due to implementation action of the experimental medical device (portal vein embolization) and which are expected, are:
  - Liver insufficiency (liver function tests) within a week
  - Peritonitis within 72 hours
  - Hemoperitoneum within 48 hours

- For serious adverse events related to the procedure of the research and which are expected (non experimental treatment required for carrying out the research):
  - the SmPC of Visipaque® (Iadixanol) should be consulted (RCP ANSM du 03/07/2015), (17)
  - the SmPC of Atarax should be consulted (RCP ANSM du 09/12/2013), (19)
  - the SmPC of Polaramine repetabs should be consulted (RCP ANSM du 13/10/2011), (20)

- For serious adverse events due to hepatectomy :
  - Cirrhotic decompensation within a week
  - Important bleeding necessitating transfusion within 48 hours

- Serious adverse events listed in section 10.3.1 when Grade $\geq$ 4 will be immediately notified to sponsor.

Ce document est la propriété du DRCD / APHP. Toute reproduction est formellement interdite.

10.6.1.1 Declaration to ANSM and CPP

The sponsor shall declare to the French National Security Agency of Medicines and Health Products (ANSM) and to the relevant protection committee (CPP):

- any suspected unexpected serious adverse reactions due to a medical device undergoing research (SUSAR)
- any serious adverse event likely to be linked with the implementation of the medical device being researched.

NOTE: The sponsor will report any incident that results in a serious adverse event to the ANSM (dedicated ANSM email for the clinical trials conducted on MD/IVD MD and materiovigilance unit).

According to Article R.1123-53 of the Code de la Santé Publique and the guidelines for sponsors of clinical trials conducted on medical devices and in vitro diagnostic medical devices (ANSM), the sponsor reports all suspected unexpected serious adverse reactions (SUSAR) and serious adverse events that may be related to the implementing gesture of the medical device in France and elsewhere, within the regulatory deadline, to the ANSM:

- for fatal or life-threatening suspected unexpected serious adverse reactions and fatal or life-threatening serious adverse events possibly related to the implementing gesture of a medical device, without delay upon knowledge by the sponsor.
- for all other suspected unexpected serious adverse reactions and serious adverse events possibly related to the implementing gesture of the medical device, within 15 days following receipt.
- for fatal or life-threatening suspected unexpected serious adverse and fatal or life-threatening serious adverse events possibly related to the implementing gesture of a medical device, additional relevant information must be sent to the ANSM within 8 days following receipt.
- for all other types of suspected unexpected serious adverse reactions and serious adverse events possibly related to the implementing gesture of a medical device, additional relevant information must be sent to the ANSM within 15 days following receipt.

A copy of these declarations shall be sent to the relevant CPP.

10.6.1.2 Information on investigators

Ce document est la propriété du DRCD / APHP. Toute reproduction est formellement interdite.

The sponsor must notify all relevant investigators about any data that could adversely affect the safety of the research subjects.

Specific cases of serious adverse events of special interest:
At the request of ANSM, the sponsor may be asked to declare serious adverse events of special interest, in accordance with the same procedures and deadlines as SUSARs.

10.6.2 Analysis and declaration of other safety data

According to Article R.1123-46 of the Code de la Santé Publique and the guidelines for sponsors of clinical trials conducted on medical devices and in vitro diagnostic medical devices (ANSM), an emerging safety issue is defined by any data which could significantly led to re-evaluation of the benefit-risk ratio of the trial or the product under investigation, modifications in the use of this product, in the conduct of the trial or in the trial documents, or to suspension, interruption or modification in the protocol of the clinical trial or other similar trials. For trials involving the first administration or use of a health product in healthy volunteers: any serious adverse reaction.

The sponsor will report to the competent authority and the Ethics Committee without delay upon knowledge any emerging safety issues and, if applicable, describe what urgent safety measures have been taken by the sponsor.

Following the initial declaration of any emerging safety issue, the sponsor will report any additional relevant information about the emerging safety issue in the form of a follow-up report, which must be sent to ANSM and CPP no later than 15 days upon knowledge of the sponsor.

If the suspected unexpected serious adverse reaction meets the definition of an emerging safety issue, the sponsor will report both the SUSAR and the emerging safety issue to the ANSM according to the appropriate modalities and within the regulatory timelines as previously described.

10.6.3 Annual safety report

Once a year for the duration of the clinical trial, the sponsor must draw up an annual safety report (RAS ou annual safety report – [ASR]) which includes, in particular:
- an analysis of the safety of the research subjects,
- a line listing of suspected serious adverse reactions and a list of serious adverse events that could be linked to the medical device’s implementation, that occurred during the period covered by the report,

Ce document est la propriété du DRCD / APHP. Toute reproduction est formellement interdite.

- a cumulative summary tabulation of serious adverse events that have occurred since the start of the research

The report must be delivered no later than 60 days after the first inclusion in the trial.

10.7 Data Safety Monitoring Board

The Data and Safety Monitoring Board (DSMB) can be established by the sponsor. Its primary mission is to serve as a committee for monitoring safety data. It can have other missions, such as monitoring efficacy data (especially if the protocol includes interim analyses).
The DSMB is mentioned in Article L. 1123-7 of the French Public Health Code.

The sponsor is responsible for justifying the creation or absence of a supervisory committee to the Competent Authority (ANSM) and to the CPP.

A DSMB will be convened for this biomedical research. The DSMB will hold its preliminary meeting before the first inclusion of the first subject. All missions as well as the precise operating methods of the DSMB are described in the charter for the research's DSMB.

10.7.1 General information about the DSMB

The DSMB makes recommendations to the sponsor about the continuation, modification or termination of the research. The recommendations that the DSMB can make are:

- to continue the research with no modifications
- to continue the research with a modification to the protocol and/or to the monitoring of subjects
- to temporarily halt inclusions
- to permanently terminate the research in light of:
  - safety data: serious adverse reactions
  - efficacy data: proven futility or efficacy

Ce document est la propriété du DRCD / APHP. Toute reproduction est formellement interdite.

The DSMB is appointed by the sponsor and is made up of 3 people with no connection to the research, including two specialists in the medication being studied and one radiologist.

The DSMB has a consultative role in advising the sponsor on safety issues such as tolerance and re-assessment of the benefit-risk ratio during the research.

The DSMB must hold its preliminary meeting before the first inclusions of the first subject and ideally before the protocol is submitted to the competent authority and the CPP. The committee's agenda will be as follows:

<u>Definition of the DSMB's missions</u>:

- Validation of the research methodology:

The proposed methodology for the clinical trial will be validated by the DSMB so that it does not jeopardise the safety of subjects, in particular relating to the inclusion methods.

- Validation of tolerance monitoring methods:
    - nature of the evaluated parameters
    - frequency of the evaluations, consultation schedule

- Validation of termination criteria:
    - criteria for terminating a subject's participation for tolerance reasons
    - criteria for the temporary or permanent termination of the research (leading to the establishment of certain recommendations ("stopping rules"))

- Modification of the protocol and recommendations:

In light of the analysis of tolerance data for the research, the DSMB can, when applicable: propose substantial modifications in order to modify certain data, in particular relating to the protocol (inclusion and non-inclusion criteria, monitoring, additional exams, etc.). Likewise the DSMB can issue any recommendations it deems useful in order to best ensure the safety of the research subjects and to maintain a favorable benefit-risk balance throughout the research.

<u>Definition of the DSMB's operating methods</u>:

Ce document est la propriété du DRCD / APHP. Toute reproduction est formellement interdite.

- meeting types (open session, then closed sessions) and schedule
- desired methods and format of SAE notification from the sponsor to the DSMB

The DSMB appoints its chairman at the first meeting.

The DSMB has a consultative role. The final decision concerning the conduct of the clinical trial relies on the sponsor.

The sponsor retains decision-making authority. When applicable, the sponsor delivers its decision, with justification, and DSMB reports to the Competent Authority (ANSM) and the CPP.

11 DATA MANAGEMENT

11.1 Data collection methods

Data collection will be performed using an eCRF, CleanWeb (Télémédecine).

The CRF will be completed for each patient included in the trial, and verification versus source data by the clinical monitor in the investigational centre will be done.

The Investigator is responsible for ensuring that, during the clinical study, the research team, which will be under his/her supervision, will comply with the site Standard Operating Procedures.

The investigator should make sure that all clinical study data recorded on a paper or electronic CRF are accurate, complete and readable.

All the clinical study information should be recorded, handled and stored in a way that allows its accurate reporting, interpretation and verification.

Data recorded in the CRF, which are drawn from source data or documents, should correspond to the data appearing in these documents. All variations should be explained, documented and approved by the investigator or his/her delegate.

CRFs will be completed only by authorized persons. This authorization should be documented.

The investigator or his/her delegate will only allow individuals with the required qualifications or training in CRFs completion and data verification to take part in this task. Confirmation of this qualification or training should be completed.

Ce document est la propriété du DRCD / APHP. Toute reproduction est formellement interdite.

For reasons of security and confidentiality of subjects and their data, only non- ambiguous subject identification codes should be used for identification of all data reported in the CRF for each subject.

The CRF will be completed as soon as the data are available or during the subject's evaluation or follow-up visit. If, for any reason, information required in the CRF cannot be provided, it is recommended that specific codes be defined for the missing data. These codes should be defined at the time the CRFs are designed.

If data are transformed during processing, it should always be possible to compare the original data and observations with the processed data. In order to respect this principle, a tracking mechanism for all modifications of the data should be established. This process of control, verification and correction should allow for comparison with the source data, and should make it possible to determine by whom, when and why the modification was made.

Any modifications made to CRFs should be justified, reviewed, authorized and approved by the investigator. To confirm this approval, it is recommended that the investigator signs and dates the CRF only when the correction process is finished and the CRF is ready to be transmitted to personnel responsible for capture and processing of the data, designated by the investigator.

Archiving of the CRFs and investigator files will be done by the trial centres according to GCP, under responsibility of the principal investigator.

11.2 Right to access source data and documents

11.2.1 Access to data

In accordance with GCPs:

- the sponsor is responsible for obtaining the permission of all parties involved in the research to guarantee direct access to all locations where the research will be carried out, to the source data, to the source documents and the reports, with the goal of quality control and audit by the sponsor

- the investigators will make available to those in charge of monitoring, quality control and audit relating to the biomedical research the documents and personal data strictly necessary for these controls, in accordance with the legislative and regulatory provisions in force (Articles L.1121-3 and R.5121-13 of the French Public Health Code).

Ce document est la propriété du DRCD / APHP. Toute reproduction est formellement interdite.

11.2.2 Source documents

Source documents are defined as any original document or object that can prove the existence or accuracy of a piece of information or a fact recorded during the research. These documents will be kept for 15 years by the investigator or by the hospital in the case of a hospital medical file.

11.2.3 Data confidentiality

Those responsible for biomedical research quality control (Article L.1121-3 of the French Public Health Code) will take all necessary precautions to ensure the confidentiality of information about the experimental devices, the research, the research subjects and in particular the identity of the subjects and the results obtained.

These individuals, as well as the investigators themselves, are subject to professional secrecy (in accordance with the conditions set out in Articles 226-13 and 226-14 of the Penal Code).

During or after the biomedical research, the data collected about the research subjects and sent to the sponsor by the investigators (or any other specialised parties) will be made non-identifying.

Under no circumstances should the names and addresses of the subjects involved be shown.

The sponsor will ensure that each research subject has given permission in writing for access to personal information about him or her which is strictly necessary for the quality control of the research.

11.3 Data processing and storage of documents and data

11.3.1 Identification of the manager and the location(s) for data processing

The Cleanweb e-CRF will be programmed by a data manager of the Paris-Sud clinical research unit.

11.3.2 Data entry

Data entry will be carried out on electronic media via a web browser. Securized login and password will be attributed a data manager of the Paris-Sud clinical research unit (URC).

Ce document est la propriété du DRCD / APHP. Toute reproduction est formellement interdite.

11.3.3 Data processing (CNIL, the French Data Protection Authority) in France

This research falls under the "Méthodologie de référence" (MR-001) according to the provisions of Article 54, paragraph 5 of modified Law No. 78-17 of 6 January 1978 relating to information technology, data files and privacy. This change was approved in a decision made on 5 January 2006. AP-HP, the research sponsor, has signed a commitment to comply with this " Méthodologie de référence ".

11.3.4 Archival

Specific documents for biomedical research relating to a medical device or *in vitro* diagnostic medical device will be archived by the investigator and the sponsor for a period of 15 years after the end of the research.

This indexed archival includes, in particular:

- A sealed envelope containing the original copies of all information sheets and consent forms signed for all individuals at the centre that participated in the research for the investigator
- A copy of all the information notes and consent forms signed for all subjects at the centre that participated in the research for the sponsor
- "Research" binders for the Investigator and the sponsor, including:
    - the successive versions of the protocol (identified by the version no. and date), and the appendices
    - the ANSM authorizations and CPP favorable opinions
    - letters of correspondence
    - the inclusion list or register
    - the appendices specific to the research
    - the final research report
- The data collection documents.

Ce document est la propriété du DRCD / APHP. Toute reproduction est formellement interdite.

11.4 Ownership of the data

AP-HP is the owner of the data, which cannot be used or disclosed to a third party without its prior approval.

12 STATISTICAL ASPECTS

Statistical analyses will be performed by the biostatistician at the URC Paris-Sud (Bicêtre Hospital) with SAS® software according to a plan drafted and validated before database lock.

12.1 Description of statistical methods to be used including the timetable for the planned interim analyses

12.1.1 Descriptive analysis

A flow chart will describe the number of eligible patients, and the number of patients included and the number of patients lost to follow-up or withdrawn from study.
Demographic variables and baseline characteristics will be summarized.
All variables will be analyzed descriptively with appropriate statistical methods: categorical variables by frequency tables and continuous variables by sample statistics (ie mean, standard deviation, minimum, median, quartiles and maximum).

12.1.2 Patients' validity

All included patients who had PVE will be valid for the safety/ intent to treat (ITT) population.
A patient is valid for the per protocol analysis if the patient:

- Is valid for safety / ITT
- Has an adequate PVE procedure
- If completing the study, has an adequate follow-up at 4-6 weeks, or if withdrawn due to death or complication occurrence
- Shows no major protocol deviations

Final decisions regarding validity will be made during the Validity Review Meeting and documented in the Validity Review Report.

12.1.3 Primary analysis

The primary variable will be the evolution of the hypertrophy ratio of FLR volume / total liver volume between the baseline and after the PVE, evaluated by computed tomography scan 4-6 weeks after PVE following the procedure. These ratios will be calculated in patients valid for safety / ITT as described in paragraph 8. This evolution will be given with the 95% confidence interval.

Depending on the number and type of protocol violations, the primary analysis of the primary endpoint may be duplicated in the per protocol set. This per protocol analysis will be supportive.

12.1.4 Secondary analysis

We will compare the estimated ratio with the classical ratio of 13% given in literature (Student T test or if necessary a non parametric Wilcoxon test).

All other secondary endpoints will be analyzed descriptively with appropriate statistical methods: categorical variables by frequency tables and continuous variables by sample statistics (ie mean, standard deviation, minimum, median, quartiles and maximum). The correspondent 95% confidence intervals will be given.

12.1.5 Interim analysis

No interim analysis is planned.

12.2 Calculation hypotheses for the number of subjects required and the result

A sample size of 44 evaluable patients is needed to obtain a 95% confidence interval, equal to the sample mean plus or minus 2% (standard deviation 6.5%).

Ce document est la propriété du DRCD / APHP. Toute reproduction est formellement interdite.

A global sample size of 50 patients will be included to take account of 10% of non evaluable patients.

12.3 Method for taking into account missing, unused or invalid data

The CRF's programming will limit invalid data as much as possible by restricting the possible values to type during online completion of CRF.
Missing data will be not replaced.

12.4 Management of modifications made to the analysis plan for the initial strategy

The statistical analysis will follow a Statistical Analysis Plan which will have to be validated by the coordinating investigator, the methodologist and the URC's biostatistician, before the database lock (at the latest). Any modification posterior to the database lock will lead to a post-hoc SAP.

13 QUALITY CONTROL AND ASSURANCE

Each biomedical research project managed by AP-HP is ranked from A to D according to the projected risk incurred by research subjects using the classification of biomedical research sponsored by AP-HP.

13.1 General organisation

The sponsor must be responsible for the safety and respect of those subjects who have agreed to participate in the research. The sponsor must implement a quality assurance system to best monitor the conduct of the research in the investigation centres.

The objectives of monitoring the research, as defined in the French Good Clinical Practices (BPC section 5.18.1), are to verify that:

- the rights, safety and protection of the research subjects are met
- the data reported is exact, complete and consistent with the source documents

Ce document est la propriété du DRCD / APHP. Toute reproduction est formellement interdite.

- the research is carried out in accordance with the protocol in force, with the French GCPs and with the legislative and regulatory provisions in force

13.1.1 Strategy for opening the centres

The strategy for opening the centres established for this research is determined using the appropriate monitoring plan.

13.1.2 Level of centre monitoring

In the case of this research, which is considered D risk, the appropriate monitoring level has been determined based on the complexity, the impact and the budget for the research. Thus, the sponsor and the coordinating investigator have agreed on the logistic score and impact, resulting in a research monitoring level to be implemented: level high

13.2 Quality control

Paris-Sud Clinical Research Unit will be responsible for the proper conduct of the research, for collecting and documenting, recording and reporting the data generated in writing, in accordance with the Standard Operating Procedures applied within the DRCD and in accordance with the French Good Clinical Practices as well as with the legislative and regulatory provisions in force.

The investigator and the members of the investigator's team agree to make themselves available during Quality Control visits carried out at regular intervals by the Clinical Research Associate. During these visits, the following elements will be reviewed:

- written consent
- compliance with the research protocol and with the procedures defined therein
- quality of the data collected in the case report form: accuracy, missing data, consistency of the data with the "source" documents (medical files, appointment books, original copies of laboratory results, etc.)

Ce document est la propriété du DRCD / APHP. Toute reproduction est formellement interdite.

- management of the products used

13.3 Case Report Form

All information required according to the protocol must be entered in the case report forms. The data must be collected as and when they are obtained, and clearly recorded in these case report forms. Each missing data item must be coded.

This digital case report form will be implemented in each of the centres thanks to a web-based data collection medium. Investigators will be given a document offering guidance in using this tool.

When the investigators complete the case report via the Internet, the CRA can view the data quickly and remotely. The investigator is responsible for the accuracy, quality and relevance of all the data entered. In addition, the data are immediately verified as they are entered, thanks to consistency checks. Thus, the investigator must validate any changes to the values in the case report form. These modifications will be subject to an audit trail. A justification can be added when applicable, as a comment. A printout, authenticated (signed and dated) by the investigator, will be requested at the end of the research. The investigator must archive a copy of the authenticated document that was delivered to the sponsor.

13.4 Management of non-compliances

Any events that occur as a result of non-compliance, by the investigator or any other individual involved in conducting the research, with the protocol, with the standard operating procedures, with the good clinical practices or with the legislative and regulatory provisions in force must be noted in a declaration of non-compliance addressed to the sponsor. As a first step, major or critical non-compliances will be reviewed and processed by the DRCD's medical coordinator in order to implement the necessary corrective or preventive actions. Next, the non-compliances will be sent to the Quality - Risk Management Division of the DRCD for verification and analysis. These verifications could result in the investigator in charge of the research location in question being asked for information or could lead to compliance or audit visits.

Ce document est la propriété du DRCD / APHP. Toute reproduction est formellement interdite.

13.5 Audits / inspections

The investigators agree to accept the quality assurance audits carried out by the sponsor as well as the inspections carried out by the competent authorities. All data, documents and reports may be subject to regulatory audits and inspections. Medical secrecy cannot be invoked in opposition to these audits and inspections.

An audit can be carried out at any time by individuals appointed by the sponsor and who are not associated with the research directors. The objective of the audit is to ensure the quality of the research, the validity of the results and compliance with the legislation and regulations in force.

The individuals who lead and monitor the research agree to comply with the sponsor's requirements and with the competent authority regarding research audits or inspections.

The audit may be applicable to all stages of the research, from the development of the protocol to the publication of the results and the organisation of the data used or produced as part of the research.

13.6 Primary investigator's commitment to assume responsibility

Before starting the research, each investigator will give the sponsor's representative a copy of his/her personal curriculum vitæ, signed and dated, with his/her number in the RPPS (Répertoire Partagé des Professionnels de Santé, Collective Database of Health Professionals).

Each investigator will undertake to comply with the legislation and to carry out the research according to French GCP, adhering to the Declaration of Helsinki terms in force.

The primary investigator at each participating centre will sign a responsibility commitment (standard DRCD document) which will be sent to the sponsor's representative.

The investigators and their employees will sign a delegation of duties form specifying each person's role.

Ce document est la propriété du DRCD / APHP. Toute reproduction est formellement interdite.

14 ETHICAL AND LEGAL CONSIDERATIONS

14.1 Methods for obtaining information and consent from research participants

In accordance with Article L1122-1-1 of the French Public Health Code, no biomedical research can be carried out on a person without free and informed consent, obtained in writing after the person has been given the information specified in Article L.1122-1 of said Code.

The subject will be granted a reflection period between the time when the subject receives the information and the time when he or she signs the consent form.

The free and informed consent, in writing, of the subject is obtained by the investigator, or by a doctor representing the investigator, before the inclusion of the subject in the research.

The information sheet and a copy of the consent form, signed and dated by the research subject and by the investigator or the doctor representing the investigator, are given to the individual prior to his or her participation in the research.

In addition, the investigator will specify in the research participant's medical file the methods used for obtaining his or her consent as well as the methods used for providing information with the goal of obtaining their consent. The investigator will retain the original signed and dated copy of the subject's consent form.

14.2 Subject prohibited from participating in another research or an exclusion period anticipated after the research, if applicable

During this period, the subject may not participate in other biomedical research protocols relating to a medical device or an *in vitro* diagnostic medical device until after 1 month.

14.3 Compensation for subjects

Patients will receive no financial compensation.

Ce document est la propriété du DRCD / APHP. Toute reproduction est formellement interdite.

14.4 Registration on the national register of subjects participating in biomedical research

As mentionned in article L.5311-1 of the French public health code, patients included in EMBORES study will not be registered in the national file of persons participating to a research protocol.

14.5 Legal obligations

14.5.1 The sponsor's role

Assistance Publique - Hôpitaux de Paris (AP-HP) is the sponsor of this research and by delegation, the Clinical Research and Development Department (DRCD) carries out the research's missions in accordance with Article L.1121-1 of the French Public Health Code. Assistance Publique - Hôpitaux de Paris reserves the right to halt the research at any time for medical or administrative reasons. In this case, notification will be sent to the investigator

14.6 Request for an opinion from the Comité de Protection des Personnes (CPP, ethical review board)

AP-HP, as sponsor, obtains for this biomedical research prior to starting the research, the favourable opinion of the appropriate CPP, within the scope of its authority and in accordance with the legislative and regulatory provisions in force.

14.7 Request for authorisation to ANSM

AP-HP, as sponsor, obtains for this biomedical research relating to a medication for human use and prior to starting the research, authorisation from the ANSM, within the scope of its authority and in accordance with the legislative and regulatory provisions in force.

14.8 Commitment to compliance with the MR 001 "Méthodologie de Reference"

AP-HP, the research sponsor, has signed a commitment to comply with this "Méthodologie de reference".

Ce document est la propriété du DRCD / APHP. Toute reproduction est formellement interdite.

14.9 Modifications to the research

Any substantial modification to the protocol by the coordinating investigator must be sent to the sponsor for approval. After approval is given, the sponsor must obtain, prior to starting the research, a favourable opinion from the CPP and authorisation from the ANSM within the scope of their respective authorities.

The information sheet and the consent form can be revised if necessary; in particular if there is a substantial modification to the research or if adverse reactions occur.

14.10 Final research report

The final biomedical research report referred to in Article R1123-60 of the French Public Health Code is drawn up and signed by the sponsor and the investigator. A summary of the report written according to the competent authority's reference plan will need to be sent to the competent authority and ethical review board within one year after the end of the research, meaning the end of the participation of the last research subject.

15 FUNDING AND INSURANCE

15.1 Funding source

The Financial support is provided by the Programme Hospitalier de Recherche Clinique – PHRC 2015 (Ministère de la Santé).

15.2 Insurance

For the duration of the research, the Sponsor will take out an insurance policy covering the sponsor's own civil liability as well as the civil liability of all the doctors involved in carrying out the research. The sponsor will also provide full compensation for all harmful consequences of the research for the research subjects and their beneficiaries, unless the sponsor can prove that the harm is not the fault of the sponsor or any agent. The act of a third party or the voluntary withdrawal of the person who initially consented to participate in the research cannot be invoked against said compensation.

Assistance Publique- Hôpitaux de Paris (AP-HP) has taken out insurance from HDI-GERLING through BIOMEDIC-INSURE for the full research period, covering its own civil

Ce document est la propriété du DRCD / APHP. Toute reproduction est formellement interdite.

liability and that of any agent (doctor or research staff), in accordance with Article L.1121-10 of the French Public Health Code.

16 PUBLICATION RULES

16.1 Mention of the affiliation of AP-HP for projects sponsored or managed by AP-HP

Results from this study may be presented at scientific congresses or/and published in peer-reviewed journals. Publications projects will be discussed by the trial's principal investigators, all co-workers implicated in the trial and the sponsor, as regard to their contents and conclusions before final versions of abstracts, texts, and posters are disclosed.

In publications resulting from this clinical trial, AP-HP will be mentioned in the affiliations or in the authors and APHP administrator (DRCD) and the funding source (if it originates from APHP) will be mentioned.

16.2 Mention of the AP-HP manager (DRCD) in the acknowledgements of the text

”The sponsor was Assistance Publique – Hôpitaux de Paris (Département de la Recherche Clinique et du Développement)”

16.3 Mention of the financier in the acknowledgements of the text

“The research was funded by a grant from Programme Hospitalier de Recherche Clinique - PHRC 2015 (Ministère de la Santé)”

This research will be registered on the website http://clinicaltrials.gov/ before the beginning of the research

17 BIBLIOGRAPHY

1. Azoulay D, Raccuia JS, Castaing D, Bismuth H. Right portal vein embolization in preparation for major hepatic resection. J Am Coll Surg. 1995;181(3): 266-9.
2. Lainas P, Boudechiche L, Osorio A, et al. Liver regeneration and recanalization time course following reversible portal vein embolization. J Hepatol. 2008;49(3): 354-62.
3. Dagher I, Nguyen TH, Groyer-Picard MT, et al. Efficient hepatocyte engraftment and long-term transgene expression after reversible portal embolization in nonhuman primates. Hepatology. 2009;49(3): 950-9.

Ce document est la propriété du DRCD / APHP. Toute reproduction est formellement interdite.

4. Yokoyama Y, Nagino M, Nimura Y, et al Mechanisms of hepatic regeneration following portal vein embolization and partial hepatectomy: a review. World J Surg 2007; 31: 367-374.

5. Capussotti L, Muratore A, Baracchi F, et al. Portal vein ligation as an efficient method of increasing the future liver remnant volume in the surgical treatment of colorectal metastases. Arch Surg. 2008;143(10): 978-82.

6. Tranchart H, Catherine L, Maitre S, Lainas P, De Laveaucoupet J, Dagher I. Efficient liver regeneration following temporary portal vein embolization with absorbable gelatin sponge powder in humans. J Vasc Interv Radiol. 2015;26(4): 507-15.

7. Ben-Menachem Y1, Coldwell DM, Young JW, Burgess AR. Hemorrhage associated with pelvic fractures: causes, diagnosis, and emergent management. Am J Roentgenol. 1991 ; 157(5): 1005-14.

8. Dondelinger RF, Trotteur G, Ghaye B, Szapiro D. Traumatic injuries: radiological hemostatic intervention at admission. Eur Radiol. 2002;12(5): 979-93.

9. Vedantham S1, Goodwin SC, McLucas B, Mohr G. Uterine artery embolization: an underused method of controlling pelvic hemorrhage. Am J Obstet Gynecol. 1997; 176(4):938-48.

10. Davis KR. Embolization of epistaxis and juvenile nasopharyngeal angiofibromas. Am J Roentgenol. 1987; 148(1):209-18.

11. Dorffner R1, Thurnher S, Prokesch R, Bankier A, Turetschek K, Schmidt A, Lammer J. Embolization of iatrogenic vascular injuries of renal transplants: immediate and follow-up results. Cardiovasc Intervent Radiol. 1998 Mar-Apr;21(2):129-34.

12. Mayer J1, Tacher V2, Novelli L2, Djabbari M2, You K2, Chiaradia M2, Deux JF2, Kobeiter H2. Post-procedure bleeding in interventional radiology. Diagn Interv Imaging. 2015 Jul-Aug;96(7-8):833-40. doi: 10.1016/j.diii.2015.06.009. Epub 2015 Jun 29.

13. Van Lienden KP, van den Esschert JW, de Graaf W, Bipat S, Lameris JS, van Gulik TM, van Delden OM. Portal vein embolization before liver resection: a systematic review. Cardiovasc Intervent Radiol. 2013; 36(1): 25-34

14. Zühlke HV1, Lorenz EM, Straub EM, Savvas V. Pathophysiology and classification of adhesions. Langenbecks Arch Chir Suppl II Verh Dtsch Ges Chir. 1990: 1009-16.

Ce document est la propriété du DRCD / APHP. Toute reproduction est formellement interdite.

15. Balzan S, Belghiti J, Farges O, Ogata S, Sauvanet A, Delefosse D, Durand F. The "50-50 criteria" on postoperative day 5: an accurate predictor of liver failure and death after hepatectomy. Ann Surg. 2005; 242(6): 824-8.

16. Dindo D, Demartines N, Clavien PA. Classification of surgical complications: a new proposal with evaluation in a cohort of 6,336 patients and results of a survey. Ann Surg 2004; 240: 205–213.

17. Visipaque. Résumé des Caractéristiques du Produit. ANSM [Internet] available from : http://agence-prd.ansm.sante.fr/php/ecodex/frames.php?specid=60960777&typedoc=R&ref=R0261332.htm

18. Chlorure de Sodium 0.9% BIOPERF. Résumé des Caractéristiques du Produit. ANSM [Internet] available from : http://agence-prd.ansm.sante.fr/php/ecodex/frames.php?specid=68768088&typedoc=R&ref=R0261177.htm

19. Atarax. Résumé des Caractéristiques du Produit. ANSM [Internet] available from : http://agence-prd.ansm.sante.fr/php/ecodex/frames.php?specid=60447340&typedoc=R&ref=R0233988.htm

20. Polaramine REPETABS,. Résumé des Caractéristiques du Produit. ANSM [Internet] available from : http://agence-prd.ansm.sante.fr/php/ecodex/frames.php?specid=68956617&typedoc=R&ref=R0199397.htm

Ce document est la propriété du DRCD / APHP. Toute reproduction est formellement interdite.

Multicentric prospective study assessing the efficiency of preoperative reversible selective portal vein embolization in patients requiring major hepatic resection

"EMBORES"

ADDENDUM AU PROTOCOLE VERSION XX

Table des matières

ADDENDUM 1: LIST OF RECRUITMENT CENTERS 67
ADDENDUM 2: LIST OF NON-RECRUITMENT CENTERS 68
ADDENDUM 3: INFLAMMATION AND ADHESIONS SCALE DURING PORTAL PEDICLES DISSECTION 69
ADDENDUM 4: CE MARKING, OPERATING MANUAL OF CURASPON® 70
ADDENDUM 5: FORM FOR REPORTING SERIOUS ADVERSE EVENTS 72
ADDENDUM 6 : FORM FOR REPORTING PREGNANCY THAT DEVELOPED DURING A BIOMEDICAL RESEARCH 76
ADDENDUM 7 : PERSONAL PROTOCOL CARD 79

Ce document est la propriété du DRCD / APHP. Toute reproduction est formellement interdite.

17.1 Addendum 1: List of recruitment centers

**Center N°**	**Hospital**	**Department (Co-investigators)**	**Fisrt name surname**
001	CHU Antoine Béclère	Department of Minimally Invasive Digestive Surgery	Pr Ibrahim DAGHER
002	CHU Beaujon	Department of Hepato-Bilio - Pancreatic Surgery and Liver Transplantation	Pr Olivier SOUBRANE
003	Institut Gustave Roussy	Department of Digestive Oncology	Dr Diane GOERE
004	CHU Henri Mondor	Department of Digestive Surgery	Pr Daniel AZOULAY
005	Institut Mutualiste Montsouris	Department of Digestive Diseases	Pr Brice GAYET
006	CHU Kremlin Bicêtre	Department of Digestive Surgery	Pr Stéphane BENOIST
007	CHU Paul Brousse	Department of Digestive Surgery	Dr Eric VIBERT

Ce document est la propriété du DRCD / APHP. Toute reproduction est formellement interdite.

17.2 Addendum 2: List of non-recruitment centers

Center N°	Hospital	Department (Co-investigators)	Fisrt name surname
501	CHU Antoine Béclère	Department of Radiology	Dr Sophie MAITRE
502	CHU Beaujon	Department of Radiology	Pr Valérie VILGRAIN
503	Institut Gustave Roussy	Department of Radiology	Pr Thierry DE BAERE
504	CHU Henri Mondor	Department of Radiology	Pr Hicham KOBEITER
505	Institut Mutualiste Montsouris	Department of Radiology	Dr Jean Brice GAYET
506	CHU Kremlin Bicêtre	Department of Radiology	Dr Olivier PLANCHE

No service of radiology is involved in the research for the Paul Brousse Hospital

Ce document est la propriété du DRCD / APHP. Toute reproduction est formellement interdite.

17.3 Addendum 3: Inflammation and Adhesions Scale during portal pedicles dissection

Scale used to assess the level of inflammation and adhesions during portal pedicles dissection according Zuhlke et al. Langenbecks Arch Chir Suppl II Verh Dtsch Ges Chir 1990.

Grade	Severity of of peripedicular adhesions
0	None
1	Filmy adhesion,easy to divide by blunt dissection
2	Stronger adhesions but easiliy divided by sharp dissection, beginning of vascularization
3	Thick vascular adhesion, lysis possible by sharp dissection only
4	Strong and dense adhesion; at risk of organ injury with division

Ce document est la propriété du DRCD / APHP. Toute reproduction est formellement interdite.

17.4 Addendum 4: CE Marking, operating manual of CURASPON®

EC Certificate

mdc medical device certification GmbH
Notified Body 0483
herewith grants

CuraMedical B.V.
Industrieweg 6B
1566 JP ASSENDELFT
Netherlands

for the scope

Curaspon®
(sterile haemostatic absorbable gelatin sponge and powder)

the

EC Design Examination Certificate

The examination of the design of the product by mdc has proven that the design meets the requirements according to

Annex II – Section 4
of the Council Directive 93/42/EEC

of 14 June 1993 concerning medical devices.

This certificate is only valid in connection with a valid mdc certificate according to Annex II – excluding section 4 for the above mentioned products.

Valid from	2012-05-08
Valid until	2017-05-07
Registration no.	4091.11.11/0
Report no.	E 4091.11 / 2012-05-07
Stuttgart	2012-05-07

Head of Certification Body

Akkreditiert durch Zentralstelle der Länder für Gesundheitsschutz bei Arzneimitteln und Medizinprodukten ZLG-ZE-975.94.04

mdc medical device certification GmbH
Kriegerstraße 6
D-70191 Stuttgart, Germany
Phone: +49-(0)711-253597-0
Fax: +49-(0)711-253597-10
Internet: http://www.mdc-ce.de

For electronic publication only

Notice d'utilisation Curaspon®

1. Description du produit
L'éponge de gélatine Curaspon® est constituée de 100% de gélatine purifiée. Son utilisation en tant qu'hémostatique résorbable est indiquée pour de multiples applications chirurgicales. Elle est insoluble dans l'eau, et complètement résorbable. Sa structure poreuse et ses interstices lui permettent d'absorber plus de 40 fois son propre poids de sang et de fluides. La capacité d'absorption de Curaspon® est fonction de son volume, augmentant avec le volume de gélatine. De plus, en raison de sa nature et de son caractère neutre, elle peut être utilisée comme support de médicament (la bibliographie pour les médicaments compatibles doit être consultée pour des informations complètes de prescription).
Elle est disponible en plusieurs tailles/formats. Elle est conditionnée individuellement en (double) blister, sache ou flacon/container, stérilisée aux rayons gamma, est à usage unique.

2. Indications
2.1 Indications générales : L'éponge de gélatine Curaspon® est utilisée au cours ou en fin de procédures chirurgicales pour réaliser une hémostase locale, par effet de tamponnement.

2.2 En chirurgie Dentaire. Curaspon® cube est une aide pour réaliser l'hémostase et un moyen de comblement pour les extractions dentaires, les amputations de racines et l'ablation de kystes, tumeurs et les dents impactées.

2.3 En chirurgie ORL, d'éponge a été prouvé que son utilisation était efficace pour le calibrage et le maintien du fascia ou du périchondre dans l'oreille moyenne (application à sec) sur site ou dans le canal de l'oreille externe après tympanoplastie.

2.4 En chirurgie rectale, hémorroïdaire et gynécologique, l'éponge de gélatine Curaspon® est utilisée pour réaliser une hémostase par effet de tamponnement.

3. Contre-indications
Allergies aux produits porcins.
L'éponge de gélatine de doit pas être utilisée au cours de la fermeture de plaies cutanées, en raison d'une éventuelle interférence avec la cicatrisation. Curaspon® ne doit pas être utilisé sans antibiotique sur les sites infectés.

4. Effets indésirables
Il n'y a pas eu de complications rapportées pour ce dispositif utilisé de manière adéquate conformément à cette notice d'utilisation et en évitant toute quantité excédentaire.
La formation de granulation tissulaire lors d'interventions sur l'oreille moyenne a été rapportée dans au moins une étude sur animaux.

5. Mode d'emploi :
L'éponge de gélatine Curaspon® peut être utilisée à sec ou saturée de sérum physiologique.
Utilisée à sec, l'éponge est découpée aux dimensions souhaitées, comprimée et appliquée sur le site hémorragique en exerçant une légère pression d'une à deux minutes jusqu'à l'obtention de l'hémostase. Utilisée humectée, l'éponge doit être immergée dans le sérum physiologique, puits comprimée entre les doigts de la main gantée (pour expulser l'air contenu dans les interstices), replacée et conservée dans le sérum jusqu'à son utilisation.
Une fois l'hémostase obtenue après des applications (parfois multiples) de l'éponge de gélatine Curaspon® peut être laissé in situ. Toute quantité excédentaire doit être retirée.
En fonction du mode d'utilisation et du site opératoire, le temps de résorption de l'éponge est de 4 semaines maximum. Appliquée pour des hémorragies de la muqueuse nasale, rectale ou vaginale, l'éponge liquéfie au bout de 2 à 5 jours. Il convient de noter qua la résorption est un processus où aussi bien te volume que les caractéristiques de résorption régressent constamment dans le temps.

5.1 La poudre de gélatine (**poudre pâte**) : La poudre est préparée par broyage d'éponges de gélatine résorbable. Ajouter un gramme de poudre dans 3 – 4 ml de sérum physiologique puis malaxer avec les doigts gantés jusqu'à obtenir la viscosité et la consistance désirées. Elle est indiquée dans les opérations impliquant une hémorragie de l'os spongieux. On peut étaler ou presser la pâte contre la surface hémorragique pour contrôler le saignement. Une application précise de la pâte sur le site hémorragique peut être réalisée à l'aide d'une seringue. Une fois que le saignement s'arrête, il faut enlever l'excès de pâte.

5.2 Eponge ORL : Découper l'éponge de gélatine de manière à l'adapter à la cavité ou à lésion auditive, nasale ou buccale puis l'insérer pour soutenir et séparer les tissus pour prévenir les adhérences et/ou pour maitriser l'hémorragie par effet de tamponnement. L'excès d'éponge doit être retiré.

Remarque importante!
Dans les cas où l'éponge de gélatine a été comprimée (par le fabricant ou l'utilisateur final), elle peut se dilater avec l'absorption de fluides (l'éponge recouvrant sa forme initiale). L'éponge Curaspon® doit être retirée après usage (si possible) et après l'hémostase dans les cavités radiculaires, les laminectomies, autours et à proximité de cavités foraminales osseuses, les zones de confinement osseux, la moelle épinière et/ou le nerf et le chiasma optiques ou les espaces tissulaires fermés avec présence d'os. Cela pourrait donner lieu à une pression indésirable contre les structures adjacentes et par là même à des douleurs chez le patient ou créer un risque potentiel de lésion nerveuse.

Il convient d'éviter les quantités excédentaires de Curaspon® notamment dans les cavités osseuses, car le retour à son volume initial peut interférer avec la fonction normale et/ou pourrait causer une éventuelle nécrose par compression des tissus environnant.
La formation de granulation tissulaire a été observée au cours d'interventions ORL sur l'oreille moyenne. Dans les cas d'infections postopératoires, une ré-intervention peut être nécessaire pour retirer la partie infecte et permettre le drainage.

Mises en garde
Ne pas utiliser lorsque l'emballage a été ouvert ou endommagé.
L'éponge de gélatine Curaspon® résorbable est un dispositif médical stérile à usage unique qui ne doit pas être re-stérilisé.

6. Stockage et manipulation – généralités
Il convient de conserver ce produit dans son emballage extérieur protecteur scellé. Avant ouverture, il faut vérifier l'emballage assurant l'intégrité de la barrière stérile. N'ouvrir l'emballage que juste avant son utilisation.

6.1 Conditionnement et stérilité
Les produits sont conditionnés stériles.
Le conditionnement consiste en :
- Emballage pour stockage et transport (conditionnement secondaire)
- Emballage stérile (conditionnement primaire)

Le conditionnement est soumis et conforme à la législation Européenne et internationale ainsi qu'aux autres standards applicables. Le conditionnement protège la gélatine des effets externes et garantie sa stérile pendant le stockage.

6.2 Manipulation de l'emballage stérile
Retirer le produit de son emballage stérile en mettant en œuvre une technique aseptique acceptable.

6.3 Stérilisation
Curaspon® est stérilise aux rayons Gamma.

6.4 Stockage
Les éponges de gélatine Curaspon® doivent être stockées au sec dans leur conditionnement d'origine à température ambiante. Eviter les rayons solaires directs.

LC5002.01.FR

Ce document est la propriété du DRCD / APHP. Toute reproduction est formellement interdite.

17.5 Addendum 5: Form for reporting Serious Adverse Events

Direction de l'Organisation Médicale et des relations avec les Universités (DOMU)<br><br>Département de la Recherche Clinique et du Développement (DRCD)	ASSISTANCE PUBLIQUE HÔPITAUX DE PARIS<br>**Formulaire de notification d'un Evènement Indésirable Grave (EIG) survenant au cours d'une Recherche Biomédicale portant sur un Dispositif Médical (DM)**	PARTIE RESERVEE AU PROMOTEUR<br>REFERENCE VIGILANCE :<br>Référence GED : REC-DTYP-0270

Dès la prise de connaissance de l'EIG par l'investigateur, ce formulaire doit être dûment complété (3 pages), signé et retourné sans délai au secteur Vigilance du DRCD-Siège par télécopie au +33 (0)1 44 84 17 99

Notification initiale ☐ **Suivi d'EIG** ☐ **N° du suivi** |__|__|

1. Identification de la recherche

**Acronyme : EMBORES**	**Date de notification :** |__|__| |__|__| |_2_|_0_|__|__| (jj mm aaaa)
**Code de la Recherche : P150953**<br>**Autre référence :**<br>**Risque :** D<br>**Plan expérimental :** Ouvert	**Date de prise de connaissance de l'EIG par l'investigateur :** |__|__| |__|__| |_2_|_0_|__|__| (jj mm aaaa)

Titre complet de la Recherche Biomédicale :
Multicentric prospective study assessing the efficiency of preoperative reversible selective portal vein embolization in patients requiring major hepatic resection

2. Identification du centre investigateur

Nom de l'établissement : ......<br>Ville et code postal : ......<br>Service :	Investigateur (nom/prénom) : ......<br>Tél : ...... Fax : ......

3. Identification et antécédents de la personne se prêtant à la recherche

Référence de la personne : |__|__|__| - |__|__|__|__|__| - |__| - |__| (n°centre - n° ordre de sélection - initiale nom - initiale prénom)

Sexe : ☐ M ☐ F

Date de naissance : |__|__| |__|__| |__|__|__|__| (jj mm aaaa)

Poids : |__|__|__| kg

Taille : |__|__|__| cm

Age : |__|__|ans

Date de signature du consentement : |__|__| |__|__| |_2_|_0_|__|__| (jj mm aaaa)

Antécédents médicaux-chirurgicaux/familiaux pertinents pour l'évaluation du cas (joindre un CRH anonymisé le cas échéant) :
......

	Date de réalisation (jj/mm/aaaa)	Chronologie: Avant la survenue de l'EIG	Chronologie: Après la survenue de l'EIG
Date de l'embolisation portale réversible par injection d'une suspension saline à base de CURASPON® POWDER et de VISIPAQUE ®:	|__|__| |__|__| |_2_|_0_|__|__| ☐ Non applicable	☐	☐
Date de l'hépatectomie :	|__|__| |__|__| |_2_|_0_|__|__| ☐ Non applicable	☐	☐

4. Description du DM expérimental *(barrer l'encadré si DM expérimental non utilisé)*

Dénomination commune / Dénomination commerciale (si marquage CE) Modèle / Version (y compris de logiciel)	N° de série	N° de lot	Indication pour laquelle le DM a été utilisé	Date d'utilisation du DM chez le participant *(jj/mm/aa)*	Si DM stérile : Date de stérilisation et date de péremption *(jj/mm/aa)*
CURASPON® POWDER	......	......	Embolisation portale réversible	le |__|__| |__|__| |__|__|	du |__|__| |__|__| |__|__| au |__|__| |__|__| |__|__|

Ce document est la propriété du DRCD / APHP. Toute reproduction est formellement interdite.

Acronyme : Erreur ! Source du renvoi introuvable.

Référence de la personne se prêtant à la recherche : |__|__|__| - |__|__|__|__|__| - |__| - |__|
n°centre - n° ordre de sélection - initiale nom - initiale prénom

PARTIE RESERVEE AU PROMOTEUR

REFERENCE VIGILANCE :

Référence GED : REC-DTYP-0270

5. Procédures et actes ajoutés par la recherche

Nom commercial (de préférence) ou Dénomination Commune Internationale	Voie(1)	Posologie (préciser l'unité ex : mg/j)	Dates d'administration *(du jj/mm/aa au jj/mm/aa)*	En cours (2)	Indication	***Action prise*** *0 : poursuite sans modification de la posologie 1 : arrêt 2 : diminution de la posologie 3 : augmentation de la posologie 4 : ne sais pas*	***Causalité de l'EIG*** *0 : non lié au médicament 1 : lié au médicament 2 : ne sais pas*
Visipaque®			du |__|__| |__|__| |__|__| au |__|__| |__|__| |__|__|	☐	Embolisation portale réversible		
Atarax®	VO		du |__|__| |__|__| |__|__| au |__|__| |__|__| |__|__|	☐	Pré-médication		
Polaramine repetabs®	VO		du |__|__| |__|__| |__|__| au |__|__| |__|__| |__|__|	☐	Pré-médication		

6. Médicament(s) concomitant(s) au moment de l'EIG, à l'exclusion de ceux utilisés pour traiter l'événement indésirable *(compléter le tableau ci-après et si nécessaire l'annexe relative aux médicaments concomitants ou barrer l'encadré si non applicable)*

⇨ **Annexe jointe au présent formulaire :** ☐ Oui ☐ Non

Nom commercial (de préférence) ou Dénomination Commune Internationale	Voie(1)	Posologie (préciser l'unité ex : mg/j)	Dates d'administration *(du jj/mm/aa au jj/mm/aa)*	En cours (2)	Indication	***Action prise*** *0 : poursuite sans modification de la posologie 1 : arrêt 2 : diminution de la posologie 3 : augmentation de la posologie 4 : ne sais pas*	***Causalité de l'EIG*** *0 : non lié au médicament 1 : lié au médicament 2 : ne sais pas*
			du |__|__| |__|__| |__|__| au |__|__| |__|__| |__|__|	☐			
			du |__|__| |__|__| |__|__| au |__|__| |__|__| |__|__|	☐			

Voie d'administration : VO=voie orale ; IM=Intramusculaire ; IV=intraveineuse ; SC=sous-cutanée ou autre (à préciser) (2) En cours au moment de la survenue de l'EIG

7. Evènement indésirable grave [EIG]

Diagnostic : ☐ Définitif ☐ Provisoire

....

....

....

Date de survenue des premiers symptômes : |__|__| |__|__| |_2_|_0_|__|__|

Préciser lesquels :

....

....

....

Fait-il suite à un incident technique (3) lié au DM ?

☐ Non ☐ Oui Si oui, décrire l'incident :

....

....

....

Date de survenue de l'incident : |__|__| |__|__| |_2_|_0_|__|__|

Des actions techniques ont-elles été prises vis-à-vis du DM ?

☐ Non ☐ Oui Si oui, préciser :

....

(3) Code de la Santé Publique : articles L. 5212-2, R. 5212-14 à 16

Organe(s) concerné(s) :

....

....

....

....

....

....

....

....

Critères de gravité :

☐ Nécessite ou prolonge l'hospitalisation :

du |__|__| |__|__| |_2_|_0_|__|__|

au |__|__| |__|__| |_2_|_0_|__|__| ☐ *en cours*

☐ Décès

☐ Mise en jeu du pronostic vital

☐ Incapacité ou handicap important ou

Ce document est la propriété du DRCD / APHP. Toute reproduction est formellement interdite.

**Date d'apparition de l'EIG :**<br>|__|__| |__|__| |_2_|_0_|__|__| jj mm aaaa<br>**Heure de survenue :** |__||__| hh |__||__| min<br>☐ *donnée manquante*	**Délai entre la date de la dernière utilisation ou de la mise en place du DM ou la date de la procédure/acte associé et la date de survenue de l'EIG :**<br>|__||__| / |__||__| |__||__| jj hh min	durable<br>☐ Anomalie ou malformation congénitale<br>☐ Autre(s) critère(s) médicalement significatif(s), préciser :<br>…………………………<br>…………………………
**L'évènement a-t-il conduit à une interruption de l'utilisation du DM expérimental?**<br>☐ Non ☐ Oui Date : |__|__| |__|__| |_2_|_0_|__|__| ☐ Non applicable<br>L'arrêt de l'utilisation du DM expérimental a été : ○ Provisoire ○ Définitif<br>Le cas échéant, date de réutilisation du DM expérimental : |__|__| |__|__| |_2_|_0_|__|__| Récidive de l'EIG après réutilisation : ○ Non ○ Oui Date : |__|__| |__|__| |_2_|_0_|__|__| ○ Non applicable<br>**Des mesures symptomatiques ont-elles été prises ?**<br>☐ Non ☐ Oui Date : |__|__| |__|__| |_2_|_0_|__|__| Préciser :<br>………………………………………………………		**Degré de sévérité – Classification Dindo Clavien :**<br>☐ Grade I ☐ Grade IV<br>☐ Grade II ☐ Grade IV a<br>☐ Grade III ☐ Grade IV b<br>☐ Grade III a ☐ Grade V<br>☐ Grade III b

Evolution de l'événement

☐ **Décès**<br>○ sans relation avec l'EIG<br>○ en relation avec l'EIG	Date : |__|__| |__|__| |_2_|_0_|__|__| jj mm aaaa	☐ **Sujet non encore rétabli**, préciser :<br>○ Etat stable ○ Amélioration ○ Aggravation
☐ **Résolu :**<br>○ sans séquelles<br>○ avec séquelles, préciser lesquelles :<br>……………………………	Date : |__|__| |__|__| |_2_|_0_|__|__| jj mm aaaa<br>|__|__| |__|__| hh min	☐ **Evolution inconnue**

8. Autre(s) étiologie(s) envisagée(s)

☐ Non ☐ Oui Si oui, préciser : ……………………………………………
……………………………………………………………………
……………………………………………………………………

9. Examen(s) complémentaire(s) réalisé(s)

☐ Non ☐ Oui Si oui, préciser date, nature et résultats : [joindre les bilans anonymisés]……………
……………………………………………………………………
……………………………………………………………………

10. Selon l'investigateur, l'événement indésirable grave est (plusieurs cases possibles)

Lié à la recherche biomédicale :

☐ Oui : ○ **au DM de la recherche : CURASPON® Powder pour embolisation portale réversible**
☐ Relation certaine ☐ Relation probable ☐ Relation possible ☐ Relation improbable (non exclue)
○ **au geste de mise en œuvre du DM de la recherche (embolisation)**
○ **aux procédures/actes de la recherche biomédicale :**
○ **Visipaque®** ☐ Relation certaine ☐ Relation probable ☐ Relation possible ☐ Relation improbable (non exclue)
○ **Atarax®** ☐ Relation certaine ☐ Relation probable ☐ Relation possible ☐ Relation improbable (non exclue)
○ **Polaramine repetabs®** ☐ Relation certaine ☐ Relation probable ☐ Relation possible ☐ Relation improbable (non exclue)
○ **à l'hépatectomie**

Ce document est la propriété du DRCD / APHP. Toute reproduction est formellement interdite.

<table>
<tr><td>Notificateur</td><td>Investigateur</td><td rowspan="3">Tampon du service :</td></tr>
<tr><td>Nom et fonction :</td><td>Nom :</td></tr>
<tr><td>Signature :</td><td>Signature :</td></tr>
</table>

☐ Non :	○ **à la progression de la maladie faisant l'objet de la recherche :** maladie nécessitant une hépatectomie majeure<br>○ **à un (ou plusieurs) médicament(s) concomitant(s) administré(s), le(s)quel(s) :** ……………………<br>○ **à une maladie intercurrente, laquelle :** ……………………<br>○ **autre, préciser :** ……………………

Ce document est la propriété du DRCD / APHP. Toute reproduction est formellement interdite.

17.6 Addendum 6 : Form for reporting Pregnancy that developed during a biomedical research

Direction de l'Organisation Médicale et des relations avec les Universités (DOMU)<br><br>Département de la Recherche Clinique et du Développement (DRCD)	ASSISTANCE PUBLIQUE HÔPITAUX DE PARIS<br><br>***Notification et suivi d'une grossesse apparue au cours d'une recherche biomédicale portant sur un Dispositif Médical***	**PARTIE RESERVEE AU PROMOTEUR**<br><br>**REFERENCE INTERNE :**<br><br>Référence GED : REC-DTYP-0185

Ce formulaire doit être dûment complété (2 pages), signé et retourné sans délai au secteur Vigilance du DRCD-Siège par télécopie au +33 (0)1 44 84 17 99

1. Identification de la recherche

Acronyme : **EMBORES**
Code P : **P150953**

Notification initiale ☐ **Suivi de notification** ☐ **N° du suivi** |__|__|

Date de notification : |__|__| |__|__| |_2_|_0_|__|__|
jj mm aaaa

Date de prise de connaissance de la grossesse par l'investigateur : |__|__| |__|__| |_2_|_0_|__|__|
jj mm aaaa

Titre complet de la Recherche Biomédicale :
Multicentric prospective study assessing the efficiency of preoperative reversible selective portal vein embolization in patients requiring major hepatic resection

2. Identification du centre investigateur

Nom de l'établissement : ....
Ville et code postal : ....
Service : ....

Investigateur (nom/prénom) : ....
Tél : Fax :

3. Identification de la personne présentant une grossesse

Référence de la personne : |__|__|__|__| - |__|__|__|__|__| - |__| - |__|
n°centre – n° ordre de sélection – initiale nom – initiale prénom

Date de naissance : |__|__| |__|__| |___|___|__|__|
Date d'inclusion : |__|__| |__|__| |_2_|_0_|__|__|

Date des dernières règles : |__|__| |__|__| |_2_|_0_|__|__|
Et/ou date début de grossesse : |__|__| |__|__| |_2_|_0_|__|__|

Cas particulier d'une exposition paternelle : ☐ Oui ☐ Non

Référence de la personne : |__|__|__|__| - |__|__|__|__|__| - |__| - |__|
n°centre – n° ordre de sélection – initiale – initiale nom prénom

Date de naissance : |__|__| |__|__| |___|___|__|__|
Date d'inclusion : |__|__| |__|__| |_2_|_0_|__|__|

Expositions au cours de la grossesse :

Tabac :	☐ non	☐ oui (préciser nombre de paquets/année) :	☐ arrêt (préciser date) :	☐ poursuite
Alcool :	☐ non	☐ oui (préciser unités OH) :	☐ arrêt (préciser date) :	☐ poursuite
Drogue :	☐ non	☐ oui (préciser substance) :	☐ arrêt (préciser date) :	☐ poursuite
Autre (préciser) :				

4. Antécédents maternels

Médicaux :

Chirurgicaux :

Obstétricaux : |__|__| geste |__|__| pare

Préciser si fausse couche, grossesse extra-utérine, interruption de grossesse (médicale ou volontaire), mort *in utero*, malformation congénitale, pathologie congénitale/néonatale non malformative, … (*nombre, date et nature/raison si applicable).*

Ce document est la propriété du DRCD / APHP. Toute reproduction est formellement interdite.

5. DM expérimental administré ou non pendant la grossesse ou s'il s'agit d'une exposition paternelle

Dénomination commune / Dénomination commerciale (si marquage CE) Modèle / Version (y compris de logiciel)	N° de série	N° de lot	Indication pour laquelle le DM a été utilisé	Date d'utilisation du DM chez le participant *(jj/mm/aa)*	Si DM stérile : Date de stérilisation et date de péremption *(jj/mm/aa)*
CURASPON® POWDER	....	....	Embolisation portale réversible	le |__|__| |__|__| |__|__|	du |__|__| |__|__| |__|__| au |__|__| |__|__| |__|__|

	Date de réalisation *(jj/mm/aaaa)*	Chronologie : Avant la survenue de l'EIG	Chronologie : Après la survenue de l'EIG
Date de l'embolisation portale réversible par injection d'une suspension saline à base de CURASPON® POWDER et de VISIPAQUE®:	|__|__| |__|__| |_2_|_0_|__|__| ☐ Non applicable	☐	☐
Date de l'hépatectomie :	|__|__| |__|__| |_2_|_0_|__|__| ☐ Non applicable	☐	☐

6. Procédures et actes ajoutés par la recherche

Nom commercial (de préférence) ou Dénomination Commune Internationale	Voie(1)	Posologie (préciser l'unité ex : mg/j)	Dates d'administration *(du jj/mm/aa au jj/mm/aa)*	En cours (2)	Indication	***Action prise*** *0 : poursuite sans modification de la posologie 1 : arrêt 2 : diminution de la posologie 3 : augmentation de la posologie 4 : ne sais pas*	***Causalité de l'EIG*** *0 : non lié au médicament 1 : lié au médicament 2 : ne sais pas*
Visipaque®			du |__|__| |__|__| |__|__| au |__|__| |__|__| |__|__|	☐	Embolisation portale réversible		
Atarax®	VO		du |__|__| |__|__| |__|__| au |__|__| |__|__| |__|__|	☐	Pré-médication		
Polaramine repetabs®	VO		du |__|__| |__|__| |__|__| au |__|__| |__|__| |__|__|	☐	Pré-médication		

7. Médicament(s) concomitants administré(s) dans le cadre du soin

(Cf. annexe « Liste relative aux médicaments concomitants » complétée :* ☐ *Oui* ☐ *Non applicable)

Nom commercial (de préférence) ou Dénomination Commune Internationale	Date de première administration	Date de dernière administration Ou en cours	Voie d'administration(1)	Posologie / 24h
	|__|__| |__|__| |_2_|_0_|__|__|	|__|__| |__|__| |_2_|_0_|__|__| ☐ En cours		
	|__|__| |__|__| |_2_|_0_|__|__|	|__|__| |__|__| |_2_|_0_|__|__| ☐ En cours		
	|__|__| |__|__| |_2_|_0_|__|__|	|__|__| |__|__| |_2_|_0_|__|__| ☐ En cours		

(1) Voie d'administration : VO=voie orale ; IM=Intramusculaire ; IV=intraveineuse ; SC=sous-cutanée ou autre (à préciser)

8. Suivi de la grossesse

☐ Echographiques. Date(s) et résultats à préciser *(joindre les CR anonymisés)* :

☐ Autres examens. Date(s) et résultats à préciser *(joindre les CR anonymisés)* :

9. Grossesse en cours ☐ (faxer un nouveau formulaire complété à l'issue de la grossesse pour le suivi de la notification initiale)

ou issue de la grossesse ☐ (compléter ci-dessous)

Ce document est la propriété du DRCD / APHP. Toute reproduction est formellement interdite.

Date : |__|__| |__|__| |_2_|_0_|__|__| Terme : |__|__| SA |__|__| J

☐ Fausse couche
→ Examen anatomo-pathologique disponible : ☐ Non ☐ Oui, précisez le résultat :

☐ Grossesse extra-utérine
→ Examen anatomo-pathologique disponible : ☐ Non ☐ Oui, précisez le résultat :

☐ Interruption de grossesse → Raison :
→ Examen anatomo-pathologique disponible : ☐ Non ☐ Oui, précisez le résultat :

☐ Accouchement : ☐ Spontané ☐ Provoqué ☐ Voie basse ☐ Césarienne

Naissance multiple : ☐ Non ☐ Oui, précisez le nombre :
Souffrance fœtale : ☐ Non ☐ Oui, précisez :
Mort-né : ☐ Non ☐ Oui, précisez :
Placenta normal : ☐ Oui ☐ Non, précisez :
Liquide amniotique : ☐ Clair ☐ Autre, précisez :
Anesthésie : ☐ Générale ☐ Péridurale ☐ Rachianesthésie ☐ Aucune

10. Nouveau-né (Si naissance multiple, compléter les parties 1, 2, 3, 9 et 10 d'un nouveau formulaire et le faxer)

Sexe : ☐ Masculin ☐ Féminin
Poids : |__|__|__|__| grammes Taille : |__|__|__| cm Périmètre crânien : |__|__|__| cm
APGAR : 1 minute : _____ 5 minutes : _____ 10 minutes : _____

Malformation(s) congénitale(s) : ☐ Non ☐ Oui, précisez :

Pathologie(s) congénitale(s)/néonatale(s) non malformative(s) : ☐ Non ☐ Oui, précisez :

Le nouveau-né a-t-il bénéficié d'un suivi particulier à la naissance : ☐ Non ☐ Oui, précisez : ☐ Non applicable

**Notificateur**	**Investigateur**	Tampon du service :
Nom et fonction :<br>Signature :	Nom :<br>Signature :	

Ce document est la propriété du DRCD / APHP. Toute reproduction est formellement interdite.

17.7 Addendum 7 : Personal protocol card

CARTE PATIENT

Nom : ………………………………… Prénom : …………………………….

Je participe à la recherche biomédicale : EMBORES

« Etude prospective multicentrique évaluant l'efficacité de l'embolisation portale sélective réversible en pré-opératoire chez les patients devant bénéficier une hépatectomie majeure»

dont le promoteur est l'AP - HP

Sous l'identifiant patient : ….…………………….

Je suis suivi par le Dr ………………………………………………

A l'hôpital ……………………………………………………….

Tel. : …………………………………

Merci de garder cette carte en permanence avec vous et de la présenter à tout professionnel de santé

Ce document est la propriété du DRCD / APHP. Toute reproduction est formellement interdite.
