# Supplementary material for: Efficacy and safety of temporary selective portal vein embolization before major hepatectomy (EMBORES): a phase 2 trial
Source: eClinicalMedicine. 2026 Oct 12;101:104262. doi: 10.1016/j.eclinm.2026.104262 (PMC13681468; doi:10.1016/j.eclinm.2026.104262)
Supplement: Supplementary file 2 [file mmc10.pdf]

FAVORABLE OPINION OF THE ETHICS COMMITTEE (CPP)

Research Project No. 16-028

Kremlin-Bicêtre, November 27, 2020

The Committee was notified on November 4, 2020 of a substantial amendment submitted by Ms. Williams on behalf of AP■HP concerning the EMBORES study.

Multicenter Prospective Study Evaluating the Efficacy of Reversible Selective Portal Vein Embolization as a Preoperative Strategy in Patients Undergoing Major Hepatectomy (EMBORES – ID■RCB No. 2016■A00878■43).

Principal Investigator: Professor DAGHER, Department of Digestive Surgery, Antoine Béclère Hospital.

The Committee reviewed Substantial Amendment No. 6 dated November 3, 2020. The amendment concerned a 6■month extension of the inclusion period, an update of the formula used to calculate future liver remnant hypertrophy, clarification of the centralized CT■scan review process for volumetric assessment, and an update of the investigators list including a change of principal investigator at one participating center.

Committee Conclusion

Considering that the amendment does not modify either the methodological integrity of the study or the protection of participating subjects, the Committee adopted the following decision:

FAVORABLE OPINION WITHOUT RESTRICTIONS

Anne■Marie TABURET

Chair

Comité de Protection des Personnes
Ile-de-France VII
Hôpital de Bicêtre – 78 rue du général Leclerc – 94275 Le Kremlin Bicêtre Cedex
Présidente : Anne-Marie TABURET – Secrétaire : Brigitte PILATE

Projet de Recherche N° : ***16-028*** Kremlin Bicêtre le 27 novembre 2020

Le Comité a été saisi le 4 novembre 2020
D'une modification substantielle par Madame WILLIAMS du DRCD – Hôpital Saint Louis – 1 avenue Claude Vellefaux – 75475 PARIS CEDEX 10 pour le compte du promoteur AP-HP – 3 avenue Victoria – 75004 PARIS concernant le projet de recherche qui avait obtenu un avis favorable du C.P.P. le 11 août 2015, le 26 juillet 2016, le 10 mai 2017, le 17 juillet 2018, le 10 octobre 2018 et le 9 janvier 2019, le 18 juillet 2019 et le 8 juin 2020 et intitulé :
«ETUDE PROSPECTIVE MULTICENTRIQUE EVALUANT L'EFFICACITE DE L'EMBOLIE PORTALE SELECTIVE REVERSIBLE EN PREOPERATOIRE CHEZ LES PATIENTS DEVANT BENEFICIER D'UNE HEPATECTOMIE MAJEURE »
(Référence du promoteur : EMBORES - ID-RCB : n° 2016-A00878-43)

dont l'investigateur principal est le Professeur DAGHER – Service de Chirurgie Digestive – Hôpital Antoine Béclère – 157 rue de la Porte de Trivaux – 92141 CLAMART Cedex

Le comité a examiné la modification substantielle n° 6 datée du 3 novembre 2020 (protocole v5.0 du 12/10/2020 et résumé v5.0 du 12/10/2020) à la séance du 18 novembre 2020 et portant sur la prolongation de la période d'inclusion de 6 mois supplémentaires, la mise à jour de la formule de calcul du taux d'hypertrophie du futur foie restant et précision sur la procédure de relecture centralisée des CT-scan pour le calcul des volumétries et la mise à jour de la liste des investigateurs avec changement d'investigateur principal dans le centre : le Dr David FUKS, investigateur principal du centre 005 – IMM est remplacé par le Pr Brice GAYET avec NIFC v4.0 du 12/10/2020, Addenda au protocole v5.0 du 12/10/2020, Courrier argumentaire de l'investigateur coordonnateur du 03/11/2020, FAMS du 03/11/2020, Tableau comparatif de la MS6 du 03/11/2020, CV et Attestation BPC du Pr Brice GAYEY et Attestation d'assurance du 04/11/2020

Membres présents lors de la délibération de votre protocole
Premier Collège :
- Recherche biomédicale : Monsieur M. BOTTLAENDER (T), Monsieur F. HIRSCH (T), Madame C. HILL (S), Monsieur G. P. de FILIPPO, pédiatre (T), Monsieur R. de BEAUREPAIRE (S) et Madame K. BOURDIC (S)
- Pharmacien : Madame A. M. TABURET (T) et Madame D. BLONDELON (S)

Deuxième collège :
- Personne qualifiée en éthique : Monsieur P. CASAURANG (T)
- Psychologue : Madame M. COSQUER (T)
- Juriste : Madame F. BOISSY (T) et Madame LAFOY (T)
- Associations agréées : Madame A. LABBE (T) et Monsieur C. COTTET (T)

Le Comité :
- considérant que l'amendement en référence ne modifie ni le respect méthodologique, ni la protection des personnes participant à l'étude

a adopté la délibération suivante :

AVIS FAVORABLE sans restriction

Anne-Marie TABURET
Présidente
