# Supplementary material for: Efficacy and safety of temporary selective portal vein embolization before major hepatectomy (EMBORES): a phase 2 trial
Source: eClinicalMedicine. 2026 Oct 12;101:104262. doi: 10.1016/j.eclinm.2026.104262 (PMC13681468; doi:10.1016/j.eclinm.2026.104262)
Supplement: Supplementary file 3 [file mmc11.pdf]

FAVORABLE OPINION OF THE ETHICS COMMITTEE (CPP)

Research Project No. 16-028

Kremlin-Bicêtre, January 15, 2021

The Committee was notified on January 4, 2021 of a substantial amendment submitted by Ms. Williams of the Department of Clinical Research and Development (DRCD), Saint-Louis Hospital, on behalf of the sponsor AP-HP, concerning the research project entitled:

Multicenter Prospective Study Evaluating the Efficacy of Reversible Selective Portal Vein Embolization as a Preoperative Strategy in Patients Undergoing Major Hepatectomy (EMBORES – ID■RCB No. 2016■A00878■43).

Principal Investigator: Professor DAGHER, Department of Digestive Surgery, Antoine Béclère Hospital, Clamart, France.

The Committee reviewed Substantial Amendment No. 7 dated January 4, 2021 during its meeting of January 13, 2021. The amendment concerned the update of the investigators list dated December 30, 2020.

Committee Conclusion

Considering that the amendment does not modify either the methodological aspects of the study or the protection of participating subjects, the Committee adopted the following decision:

FAVORABLE OPINION WITHOUT RESTRICTIONS

Anne■Marie TABURET

Chair

Comité de Protection des Personnes
Ile-de-France VII
BICETRE
Hôpital de Bicêtre – 78 rue du général Leclerc – 94275 Le Kremlin Bicêtre Cedex
Présidente : Anne-Marie TABURET – Secrétaire : Brigitte PILATE

Projet de Recherche N° : ***16-028*** Kremlin Bicêtre le 15 janvier 2021

Le Comité a été saisi le 4 janvier 2021
D'une modification substantielle par Madame WILLIAMS du DRCD – Hôpital Saint Louis – 1 avenue Claude Vellefaux – 75475 PARIS CEDEX 10 pour le compte du promoteur AP-HP – 3 avenue Victoria – 75004 PARIS concernant le projet de recherche qui avait obtenu un avis favorable du C.P.P. le 11 août 2015, le 26 juillet 2016, le 10 mai 2017, le 17 juillet 2018, le 10 octobre 2018 et le 9 janvier 2019, le 18 juillet 2019, le 8 juin 2020 et le 27 novembre 2020 et intitulé :
«ETUDE PROSPECTIVE MULTICENTRIQUE EVALUANT L'EFFICACITE DE L'EMBOLIE PORTALE SELECTIVE REVERSIBLE EN PREOPERATOIRE CHEZ LES PATIENTS DEVANT BENEFICIER D'UNE HEPATECTOMIE MAJEURE »
(Référence du promoteur : EMBORES - ID-RCB : n° 2016-A00878-43)

dont l'investigateur principal est le Professeur DAGHER – Service de Chirurgie Digestive – Hôpital Antoine Béclère – 157 rue de la Porte de Trivaux – 92141 CLAMART Cedex

Le comité a examiné la modification substantielle n° 7 datée du 4 janvier 2021 à la séance du 13 janvier 2021 et portant sur la mise à jour de la liste des investigateurs du 30/12/2020

<u>Membres présents lors de la délibération de votre protocole</u>
Premier Collège :
- Recherche biomédicale : Monsieur M. BOTTLAENDER (T), Monsieur F. HIRSCH (T), Madame C. HILL, épidémiologiste (S), Monsieur G. P. de FILIPPO, pédiatre (T) et Madame K. BOURDIC (S)
- Pharmacien : Madame A. M. TABURET (T) et Madame D. BLONDELON (S)

Deuxième collège :
- Personne qualifiée en éthique : Monsieur P. CASAURANG (T)
- Psychologue : Madame M. COSQUER (T)
- Juriste : Madame F. BOISSY (T)
- Associations agréées : Madame A. LABBE (T), Monsieur C. COTTET (T) et Monsieur G. MARDUEL (S)

Le Comité :
- considérant que l'amendement en référence ne modifie ni le respect méthodologique, ni la protection des personnes participant à l'étude

a adopté la délibération suivante :

AVIS FAVORABLE sans restriction

Anne-Marie TABURET
Présidente
