# Supplementary material for: Efficacy and safety of temporary selective portal vein embolization before major hepatectomy (EMBORES): a phase 2 trial
Source: eClinicalMedicine. 2026 Oct 12;101:104262. doi: 10.1016/j.eclinm.2026.104262 (PMC13681468; doi:10.1016/j.eclinm.2026.104262)
Supplement: Supplementary file 4 [file mmc12.pdf]

Opinion of the Île-de-France VII Ethics Committee on a Substantial Amendment

CPP Information

Name of the Ethics Committee: Île-de-France VII Ethics Committee
Address: Hôpital de Bicêtre, 78 rue du Général Leclerc, 94270 Le Kremlin-Bicêtre, France
Email: cpp.idf.7-bicetre@orange.fr

Sponsor Information

Organization: ASSISTANCE PUBLIQUE – HÔPITAUX DE PARIS
Name: Sabrina Williams

Investigator

Principal Investigator: Prof. Ibrahim DAGHER

File Information

SI Number: 16.00500.016028-MS09
National Number: 2016-A00878-43
Internal Reference: P150953
Regulatory Framework: Jardé Law
Research Category: Category 1
Product or Procedure: Non-invasive medical device

Title: Multicenter Prospective Study Evaluating the Efficacy of Reversible Selective Portal Vein Embolization as a Preoperative Strategy in Patients Undergoing Major Hepatectomy

Substantial Amendment Number: 9

Reason for the Substantial Amendment: Change of Principal Investigator in the Department of Surgery at Beaujon University Hospital: Professor Olivier SOUBRANE is replaced by Professor Mickaël LESURTEL.

Summary of Previous Opinions

This application was reviewed during the committee meeting of March 28, 2022. Based on the submitted documentation, the following opinion was issued on March 30, 2022. The date of notification of the opinion in the information system shall be considered the official date.

FAVORABLE OPINION

Persons Participating in the Deliberation

Documents Reviewed by the Ethics Committee (tables intentionally left untranslated in the original document).

March 30, 2022

Anne-Marie TABURET

Chair of the Ethics Committee

Comité de Protection des Personnes
Ile-de-France VII
BICETRE

Comité de Protection des Personnes Ile-de-France VII
CHU de BICETRE – 78 rue du Général Leclerc – 94275 LE KREMLIN BICETRE CEDEX

Bureau :
Présidente Anne-Marie TABURET
Vice Présidente Françoise BOISSY
Secrétaires généraux Claude COTTET
François HIRSCH
Michel BOTTLAENDER
Catherine HILL

Secrétariat : Madame PILATE-DAUSSY et Madame HAYE
(Portail des Champs – Secteur Bleu – Porte 74 bis – RDC)
E-mail : cpp.idf.7-bicetre@wanadoo.fr
Site internet : http://cpp.idf.7.bicetre.free.fr
Téléphone : 01 45 21 28 46 – Télécopie : 01 45 21 21 45
Portable : 06 21 25 49 30

Premier Collège
Michel BOTTLAENDER Médecin investigateur
François HIRSCH chercheur
Thi Chien TRAN Epidémiologiste
Gian Paolo DE FILIPPO pédiatre
Renaud de BEAUREPAIRE Neurobiologiste
Katia BOURDIC Technicienne d'étude clinique
Catherine HILL Epidémiologiste
Anne-Marie TABURET Pharmacien hospitalier
Muriel BOCQUENTIN Pharmacien hospitalier

Deuxième Collège
Pascal CASAURANG Ethique
Agnès SPIQUEL Ethique
Mireille COSQUER Psychologue
Françoise BOISSY Juriste
Valérie-Ann LAFOY Juriste
Claire OLIVIER Juriste
Annie LABBE Représentant des associations
Claude COTTET Représentant des associations
Georges MARDUEL Représentant des associations

Avis du Comité de protection des personnes Ile de France VII sur Modification Substantielle

Informations CPP

Nom du CPP : Comité de protection des personnes Ile de France VII
Adresse : Hôpital de Bicêtre - 78 rue du Général Leclerc 94270 LE KREMLIN BICETRE France
Courriel : cpp.idf.7-bicetre@orange.fr
Numéro de téléphone :

Informations promoteur

Organisme : ASSISTANCE PUBLIQUE - HOPITAUX DE PARIS
Nom et prénom : Williams Sabrina

Investigateur

Investigateur : Pr Ibrahim DAGHER

Informations dossier

Numéro SI : 16.00500.016028-MS09
Numéro national : 2016-A00878-43
Référence interne : P150953
Règlementation : Loi Jardé
Qualification de recherche : Catégorie 1
Produit ou acte : Dispositif médical non invasif

Titre : Multicentric prospective study assessing the efficiency of preoperative reversible selective portal vein embolization in patients requiring major hepatic resection
Numéro de la MS : 9
Motif de la MS : Changement d'Investigateur principal du service de chirurgie du CHU de Beaujon : le Pr Olivier SOUBRANE est remplacé par le Pr Mickaël LESURTEL

Récapitulatif des avis antérieurs

Dossier	Avis	Date d'émission
16.00500.016028	*Favorable*	31/01/2022

Ce dossier a été étudié en séance le 28/03/2022. Au vu du dossier soumis, l'avis suivant a donc été émis le 30/03/2022. C'est la date de la notification de l'avis sur le SI qui fait foi.

Avis favorable

Personnes ayant délibéré

Collège	Catégorie	Nom et prénom
Collège I	Qualification RIPH - Biostatistique ou épidémiologie	TRAN Thi Chien
Collège I	Qualification RIPH - Biostatistique ou épidémiologie	HILL Catherine
Collège I	Qualification RIPH - Autre	de FILIPPO Gian Paolo
Collège I	Pharmacien hospitalier	TABURET Anne-Marie
Collège I	Qualification RIPH - Autre	BOTTLAENDER Michel
Collège I	Pharmacien hospitalier	BOCQUENTIN Muriel
Collège I	Qualification RIPH - Autre	HIRSCH François
Collège II	Représentant d'association agréée	MARDUEL Georges
Collège II	Représentant d'association agréée	LABBE Annie

Documents analysés par le CPP

Catégorisation	Intitulé	Date de dépôt
ATT - Attestation	*2018-A02404-51_PENOFOR_GCP_Pr Lesurtel_20210604.pdf*	15/03/2022
CVI - CV investigateurs	*2018-A02404-51_PENOFOR_CV_Lesurtel_20211006.pdf*	15/03/2022
Courrier de demande de modification substantielle	*EMBORES_Courrier saisine CPP_MS9_20220315.pdf*	15/03/2022

Formulaire de demande de MS	*EMBORES_FAMS_MS9_CPP_20220315.pdf*	15/03/2022
JUS - Justification lieux de recherche	*EMBORES_justif-adequation-moyens_MS9_20220315.pdf*	15/03/2022
LIS - Liste investigateurs	*EMBORES_Addendum1_ListeCentres_20220111.pdf*	15/03/2022
Les informations justifiant le bien-fondé de chaque modification	*EMBORES_tableau-comparatif-MS9_20220111.pdf*	15/03/2022
Tableau comparatif mettant en évidence les modifications apportées dans les documents de la demande initiale	*EMBORES_tableau-comparatif-MS9_20220111.pdf*	15/03/2022
Tableau récapitulatif de l'ensemble des MS et MNS survenues depuis le DI	*EMBORES_Tableau récapitulatif des MS et MNS_MS9_20220315.pdf*	18/03/2022

Le 30 Mars 2022

Anne-Marie TABURET

Présidente du CPP
