# Supplementary material for: Efficacy and safety of temporary selective portal vein embolization before major hepatectomy (EMBORES): a phase 2 trial
Source: eClinicalMedicine. 2026 Oct 12;101:104262. doi: 10.1016/j.eclinm.2026.104262 (PMC13681468; doi:10.1016/j.eclinm.2026.104262)
Supplement: Supplementary file 5 [file mmc2.pdf]

Committee for the Protection of Persons (CPP) – Ile-de-France VII

Kremlin-Bicêtre, February 7, 2017

Research Project No. 16-028

The Committee reviewed the research project entitled:
“Multicenter Prospective Study Evaluating the Efficacy of Reversible Selective Portal Vein Embolization as a Preoperative Strategy in Patients Undergoing Major Hepatectomy”
(EMBORES – ID-RCB No. 2016-A00878-43).

Principal Investigator: Professor Dagher, Department of Digestive Surgery, Antoine-Béclère Hospital, Clamart, France.

The Committee examined the protocol, study summary, investigational device documentation, responses to questions raised during previous reviews, investigator list, and the patient information sheet and informed consent form.

After deliberation, the Committee considered: the scientific value of the research project; the appropriateness of the proposed methodology; the adequacy of the informed consent process; the favorable benefit–risk balance for participating patients. The Committee therefore issued the following decision:

FAVORABLE OPINION WITHOUT RESTRICTIONS

Signed on behalf of the Committee.
François Hirsch
Chair of the Session of December 7, 2016

Comité de Protection des Personnes
Ile-de-France VII

Hôpital de Bicêtre – 78 rue du général Leclerc – 94275 Le Kremlin Bicêtre Cedex
Présidente : Françoise BOISSY – Secrétaire : Brigitte PILATE

Projet de Recherche N° : ***16-028*** Kremlin Bicêtre le 7 février 2017

Le Comité a été saisi le 25 août 2016

Par Madame FAVREL FEUILLADE du DRCD – Hôpital Saint Louis – 1 avenue Claude Vellefaux – 75475 PARIS CEDEX 10 pour le compte du promoteur AP-HP – 3 avenue Victoria – 75004 PARIS
concernant le projet de recherche intitulé :
«ETUDE PROSPECTIVE MULTICENTRIQUE EVALUANT L'EFFICACITE DE L'EMBOLIE PORTALE SELECTIVE REVERSIBLE EN PREOPERATOIRE CHEZ LES PATIENTS DEVANT BENEFICIER D'UNE HEPATECTOMIE MAJEURE »
(Référence du promoteur : EMBORES - ID-RCB : n° 2016-A00878-43)

dont l'investigateur principal est le Professeur DAGHER – Service de Chirurgie Digestive – Hôpital Antoine Béclère – 157 rue de la Porte de Trivaux – 92141 CLAMART Cedex

Le comité a examiné les informations relatives à ce projet (protocole version n° 1 du 24/08/2016, résumé version française n° 1 du24/08/2016 et document technique du dispositif médical) et les réponses que vous lui avez apporté issues des questions de la première lecture lors des séances du 14 septembre 2016 et du 7 décembre 2016 avec liste des investigateurs version n° 1 du 24/08/2016) et lettre d'information et de consentement version française n° 1.1 du 28/11/2016

<u>Membres présents lors de la délibération de votre protocole</u>
Premier Collège :
- Recherche biomédicale : Madame C RUBINO épidémiologiste (S), Monsieur R. de BEAUREPAIRE (T), Monsieur M. BOTTLAENDER (S) et Monsieur F. HIRSCH (T)
- Pharmacien : Madame D. BLONDELON (S) (T)
- Infirmière : Madame B. LEVY(T)

Deuxième collège :
- Ethique : Monsieur P. CASAURANG (T)
- Juristes : Madame F. BOISSY (T) et Madame V. A. LAFOY (T)
- Associations agréées : Monsieur C. COTTET (T) et Madame A. LABBE (T)

Le Comité :
- **considérant l'intérêt du projet de recherche**
- **le respect d'une méthodologie adaptée à la question posée**
- **considérant le respect d'un consentement libre et éclairé formulé au regard d'une note d'information adaptée**
- **considérant la balance bénéfice risque favorable pour le patient**

a adopté la délibération suivante :

AVIS FAVORABLE sans restriction

François HIRSCH
Président à la séance du 7 décembre 2016
