# Supplementary material for: Efficacy and safety of temporary selective portal vein embolization before major hepatectomy (EMBORES): a phase 2 trial
Source: eClinicalMedicine. 2026 Oct 12;101:104262. doi: 10.1016/j.eclinm.2026.104262 (PMC13681468; doi:10.1016/j.eclinm.2026.104262)
Supplement: Supplementary file 6 [file mmc3.pdf]

Statistical Analysis Plan

Multicenter Prospective Study Evaluating the Efficacy of Reversible Partial Portal Vein Embolization as a Preoperative Strategy in Patients Requiring Major Hepatectomy

EMBORES

Date: May 2, 2024

Version: 1.0

Name	Role
Author: Kawther NHERI	Biostatistician
Reviewed by: Prof. Lamiae GRIMALDI	Head of Clinical Research Unit (CRU) – Methodologist
Approved by: Prof. Hadrien TRANCHART	Principal Investigator

Table of Contents

1. PREAMBLE
2. STUDY OBJECTIVES AND ENDPOINTS
2.1 Objectives
2.1.1 Primary Objective
2.1.2 Secondary Objectives
2.2 Endpoints
2.2.1 Primary Endpoint
2.2.2 Secondary Endpoints
3. STUDY METHODOLOGY
4. NUMBER OF SUBJECTS
5. GENERAL CONSIDERATIONS
6. SUMMARY OF STUDY DATA
7. FLOW DIAGRAM
8. STATISTICAL ANALYSIS
9. SAFETY ANALYSIS
10. SENSITIVITY ANALYSIS
11. STATISTICAL REPORT

1. Preamble

The statistical analysis of the EMBORES study will be performed in accordance with the Statistical Analysis Plan described below. The Statistical Analysis Plan, prepared by the statistician, will subsequently be validated by the Principal Investigator. Final validation will take place immediately before the start of the statistical analysis, following review of the latest data management quality control checks and a preliminary description of the available sample sizes for each variable. Statistical analyses may only begin after the database has been locked by the data management team.

2. Study Objectives and Endpoints

Primary objective: To prospectively estimate the change in the hypertrophy ratio of the future liver remnant (FLR) volume following reversible portal vein embolization.

Secondary objectives: compare FLR hypertrophy after reversible PVE with conventional irreversible PVE (13%), assess feasibility, morbidity, portal recanalization, completion of planned surgery, and perioperative/postoperative morbidity.

Primary endpoint: Change in FLR hypertrophy ratio between baseline and 4–6 weeks after portal vein embolization assessed by CT volumetry.

Secondary endpoints: FLR hypertrophy ratio, completed embolization procedures, peri/post-procedural morbidity, portal recanalization, major hepatectomy rate, inflammation/adhesions during portal pedicle dissection, and intra/postoperative morbidity.

3. Study Methodology

This is a prospective single-arm feasibility and safety study with a 36-month enrollment period and a maximum follow-up period of 18 months.

The objective of this study is to evaluate the safety and efficacy of preoperative reversible portal vein embolization (rPVE) in patients requiring major liver resection.

Portal vein embolization (PVE) is used before major hepatectomy to induce hypertrophy of the future liver remnant (FLR).

4. Number of Subjects

A sample size of 44 evaluable patients is required to obtain a 95% confidence interval with a precision of ±2% and a standard deviation of 6.5%.

To account for an anticipated 10% loss to follow-up, a total of 50 patients will be enrolled.

5. General Considerations

The final analysis will be performed using the validated and locked database after finalization and approval of this Statistical Analysis Plan.

5.1 Definition of Study Populations

5.1.1 Description of Protocol Deviations

5.1.1.1 Description of Deviations from Inclusion and Exclusion Criteria

Patients who do not fulfill all required inclusion criteria and/or who meet one or more exclusion criteria will be identified and listed.

5.1.1.2 Description of Deviations Related to Reversible Portal Vein Embolization

- Patients who did not undergo rPVE
- Duration of the rPVE procedure outside the predefined range of 45 to 120 minutes
- Volumetric CT scan and post-rPVE abdominal CT scan not performed between 4 and 6 weeks after inclusion
- Pre-embolization visit not scheduled on the day before embolization
- rPVE not performed within 2 months after the screening abdominal CT scan
- Treatment not discontinued 24 hours before embolization
- Missing data at Day 0, Day 1, Day 7, Week 4–6, Month 3, Month 6, or Month 12
- Follow-up visits not performed according to protocol schedule

5.1.1.3 Description of Deviations Related to Hepatectomy

- Patients who did not undergo hepatectomy
- Hepatectomy not performed between Week 4 and Week 8 after rPVE
- Postoperative follow-up visits not performed within protocol windows

5.1.2 Description of Analysis Populations

Included Population:
Patients who satisfy all inclusion criteria and none of the exclusion criteria.

Analysis Population:
Included patients who underwent reversible portal vein embolization (rPVE).

6. Summary of Study Data

Quantitative variables will be summarized using mean, standard deviation, median, quartiles, minimum and maximum.

Qualitative variables will be summarized using frequency tables including counts, percentages, and missing values.

6.1 Derived Variables

%FLR Volume Pre-PVE
%FLR Volume Post-PVE
FLR Volume Hypertrophy Ratio
Change in FLR Volume Hypertrophy Ratio
Time from Inclusion CT Scan to rPVE
Time from rPVE to Hepatectomy

6.2 Conventions and Software

Statistical analyses will be conducted using SAS version 9.4 or later.

7. Flow Diagram

A study flow chart will be generated describing included, non-included, withdrawn and evaluated patients.

Figure 1: Flow Chart

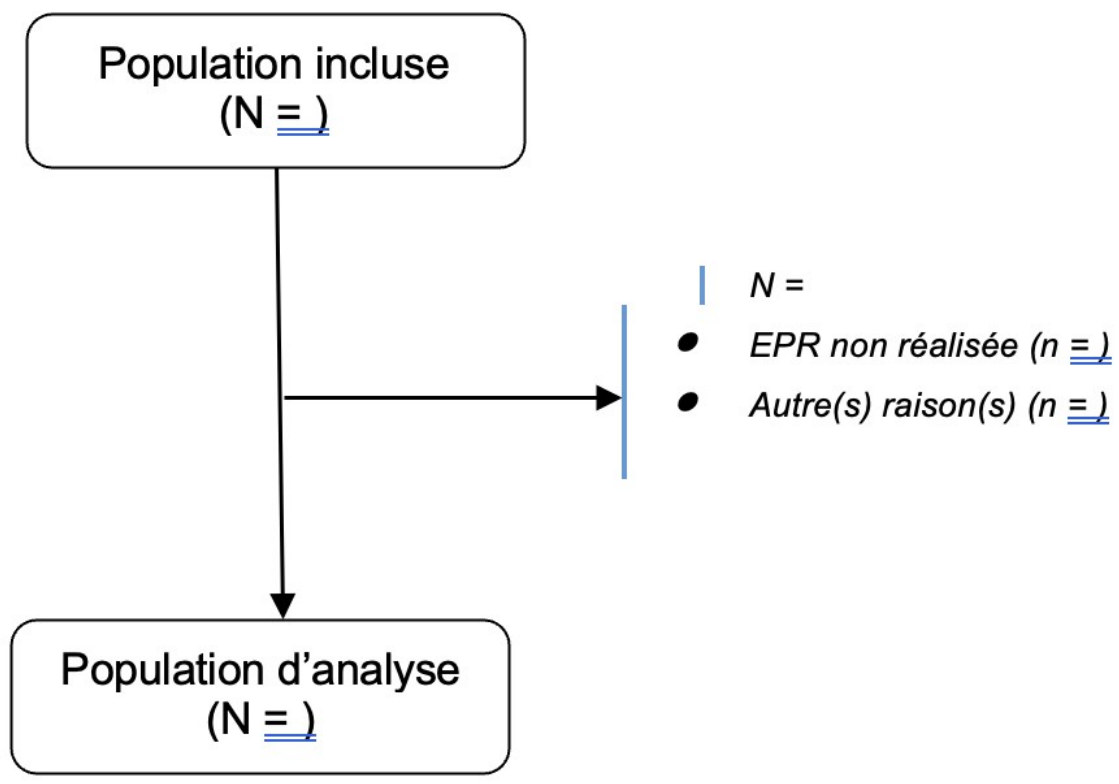

8. Statistical Analysis

8.1 Descriptive Analysis

Table 1. Demographic and Clinical Characteristics at Inclusion

Population (N =)

Age (years)
N
Mean (SD)
Median (IQR)
Min, Max

Sex, n (%)
Female
Male

Weight (kg)
Height (cm)
Body Mass Index (BMI) (kg/m^2)

Systolic Blood Pressure (mmHg)
Diastolic Blood Pressure (mmHg)
Mean Arterial Pressure (mmHg)
Heart Rate (beats/min)

Creatinine (mL/min)

Liver Status, n (%)
Normal
Steatosis
Fibrosis
Cirrhosis
Chemotherapy

Underlying Liver Disease, n (%)
None
Alcohol-related liver disease
HBV
HCV
Hemochromatosis
Other

Surgical Indication, n (%)
Hepatocellular carcinoma (HCC)
Cholangiocarcinoma
Colorectal liver metastases
Other

Table 2. Medical History

Population (N =)

ASA Classification (American Society of Anesthesiologists), n (%)
Class I
Class II
Class III

Previous Surgical History, n (%)
Yes
No

Medical History, n (%)
Yes
No

History of Other Cancer, n (%)
Yes
No

Previous Chemotherapy, n (%)
Yes
No

Number of Chemotherapy Lines, n (%)
1
2
3
4

Treatment: Anticoagulants, n (%)
Yes
No

Treatment: Antiplatelet Therapy, n (%)
Yes
No

Table 3. Pre-rPVE Abdominal CT Scan Findings

Pre-rPVE (N =)

Hepatic Tumor Location, n (%)
Right liver
Left liver
Other

Liver Segment Involvement, n (%)
Segment 1
Segment 2
Segment 3
Segment 4
Segment 5
Segment 6
Segment 7
Segment 8

Number of Liver Segments Involved, n (%)
3
4
5
6
7
8

Number of Hepatic Tumors
N
Mean (SD)
Median (IQR)
Min, Max

Portal Venous Anatomy Assessment, n (%)
Normal
Anatomical variation
Thrombosis
Other

Hepatic Vein Assessment, n (%)
Normal
Anatomical variation
Thrombosis
Other

Hepatic Artery Assessment, n (%)
Normal
Anatomical variation
Other

Table 4. Pre-rPVE Liver Volumetry

Pre-rPVE (N =)

Total Liver Volume (mL)
Tumor Volume (mL)
Tumor Volume Percentage (%)

Estimated Liver Volume to be Embolized (mL)
Percentage of Liver Volume to be Embolized (%)

Estimated Non-Embolized Liver Volume (mL)
Percentage of Non-Embolized Liver Volume (%)

Estimated Tumor Volume in the Non-Embolized Liver (mL)

For all variables:
N
Mean (SD)
Median (IQR)
Min, Max

Table 5. Reversible Portal Vein Embolization

Population (N =)

Procedure Duration (minutes)

Time Between Inclusion and Embolization (days)

Mean Arterial Pressure (mmHg)

Heart Rate (beats/min)

Type of Embolization, n (%)
Right
Left
Other

Planned Procedure Completed, n (%)
Yes
No

Proximal or Distal Embolization, n (%)
Proximal
Distal
Distal and Proximal

For continuous variables:
N
Mean (SD)
Median (IQR)
Min, Max

Table 6. Post-rPVE Abdominal CT Scan Findings

Post-rPVE (N =)

Hepatic Tumor Location, n (%)
Right liver
Left liver
Other

Liver Segment Involvement, n (%)
Segment 1–8

Number of Liver Segments Involved, n (%)
3
4
5
6
7
8

Number of Hepatic Tumors
N
Mean (SD)
Median (IQR)
Min, Max

Recanalization of Embolized Portal Branches, n (%)
None
Proximal
Distal

Table 7. Post-rPVE Liver Volumetry

Post-rPVE (N =)

Total Liver Volume (mL)
Tumor Volume (mL)
Tumor Volume Percentage (%)

Tumor Atrophy Rate (%)

Embolized Liver Volume (mL)
Percentage of Embolized Liver Volume (%)

Liver Atrophy Rate (%)

Non-Embolized Liver Volume (mL)
Percentage of Non-Embolized Liver Volume (%)

Liver Hypertrophy Rate (%)

Tumor Volume in the Non-Embolized Liver (mL)

For all quantitative variables:
N
Mean (SD)
Median (IQR)
Min, Max

Table 8. Peri- and Post-rPVE Laboratory Assessment

Time points: Day 0 (D0), Day 1 (D1), Day 7 (D7)

Red Blood Cells (10^9/L)
Hemoglobin (g/dL)
Hematocrit (%)
Mean Corpuscular Volume (fL)
Mean Corpuscular Hemoglobin Concentration (g/dL)
White Blood Cells (10^6/L)
Reticulocytes (10^6/L)
Unconjugated Bilirubin (µmol/L)
Prothrombin Time (%)

For each parameter:
N
Mean (SD)
Median
IQR
Min, Max

Table 9. Liver Function Blood Tests

Time points: Day 0 (D0), Day 1 (D1), Day 7 (D7)

AST (IU/L)
ALT (IU/L)
GGT (IU/L)
Alkaline Phosphatase (IU/L)
Total Bilirubin (µmol/L)
Conjugated Bilirubin (µmol/L)

For each parameter:
N
Mean (SD)
Median (IQR)
Min, Max

Table 10. Hepatectomy

Population (N =)

Procedure Duration (minutes)

Time Between rPVE and Hepatectomy (days)

Type of Hepatectomy, n (%)
Right Hepatectomy
Other

Surgical Approach, n (%)
Open Surgery (Laparotomy)
Laparoscopy

Blood Loss (mL)

For quantitative variables:
N
Mean (SD)
Median (IQR)
Min, Max

Transfusion, n (%)
Yes
No

Length of Hospital Stay for Hepatectomy (days)

N
Mean (SD)
Median (IQR)
Min, Max

Table 11. Description of Changes in Perioperative and Postoperative Laboratory Parameters Following Hepatectomy

Variables:
Red Blood Cells
Hemoglobin
Hematocrit
MCV
MCHC
White Blood Cells
Reticulocytes
Unconjugated Bilirubin
Prothrombin Time

Assessment time points:
D0, D5, Month 1, Month 3, Month 6, Month 12

Table 12. Description of Liver Function Blood Tests at D0, D5, Month 1, Month 3, Month 6 and Month 12

AST
ALT
GGT
Alkaline Phosphatase
Total Bilirubin

Boxplots will also be provided.

Table 13. Description of Changes in Clinical Examination Findings Following Hepatectomy

Weight
Heart Rate
Mean Arterial Pressure
Current Medications

Assessment time points:
D0, D5, Month 1, Month 3, Month 6 and Month 12

8.2 Analysis of the Primary Endpoint
The analysis of the primary endpoint will be performed in the Analysis Population.

The calculation of the primary endpoint is described in Section 6.1.

Confidence Interval Formula:
$\mu \pm 1.96 \times \sigma / \text{sqrt}(n)$

Table 14. Primary Analysis

Change in Hypertrophy Ratio, Mean (SD), 95% Confidence Interval

8.3 Analysis of Secondary Endpoints
1) Comparison of the hypertrophy ratio after reversible portal vein embolization (rPVE) with the theoretical hypertrophy ratio of conventional irreversible PVE (13%).

The comparison between the observed hypertrophy ratio and the theoretical 13% ratio will be performed using Student's t-test or the non-parametric Wilcoxon test, depending on distributional assumptions.

Table 15. Comparison of Hypertrophy Ratio with the 13% Reference Value

- Hypertrophy Ratio (%), Mean (SD)
- Theoretical Ratio (%)
- p-value
- 95% Confidence Interval

2) Demonstration of the benefits associated with reversible portal vein embolization

Table 16. Description of the Percentage of Completed rPVE Procedures

Table 17. Peri-procedural and Post-procedural Morbidity (Embolization)

- Intra-procedural morbidity
- Post-procedural morbidity
- Post-embolization morbidity
- Post-embolization complications:
  • Portal vein thrombosis extension
  • Pulmonary embolism
  • Other complications

Table 18. Portal Recanalization Rate

- None

- Partial
- Complete

Table 19. Major Hepatectomy Rate

- Yes
- No

3) Demonstration of the benefits associated with hepatectomy

Table 20. Inflammation and Adhesion Scale During Portal Pedicle Dissection

Grade 0 – None
Grade 1 – Filmy adhesions, easily divided by blunt dissection
Grade 2 – Stronger adhesions, easily divided by sharp dissection, beginning vascularization
Grade 3 – Thick vascular adhesions requiring sharp dissection
Grade 4 – Dense adhesions with risk of organ injury

Table 21. Intraoperative and Postoperative Morbidity

- Intraoperative morbidity
- Postoperative liver-related morbidity
- Liver failure
- Hemorrhage
- Biliary fistula
- Ascites
- Cirrhotic decompensation
- Biliary collection
- General postoperative morbidity
- Intraoperative or postoperative mortality

9. Safety Analysis

Descriptive analyses of adverse events (AEs) and serious adverse events (SAEs) will be performed in patients who underwent rPVE. No formal hypothesis testing is planned.

Table 22. Adverse Event Listing

Patient
Nature of Event
Presence Before End of Follow-up
Severity
Frequency

Periods:
• Between rPVE and hepatectomy
• Between hepatectomy and end of follow-up (Month 12)

Table 23. Serious Adverse Event Listing

Patient
Nature of Event
Affected Organ
Severity Grade (Clavien–Dindo Classification)
Relationship to Procedure

Periods:
• Between rPVE and hepatectomy
• Between hepatectomy and end of follow-up (Month 12)

Deaths will be described, including timing and cause of death.

10. Statistical Report

At the completion of the analysis, all results will be summarized and interpreted from a statistical perspective by the statistician in a statistical report, which will be reviewed with the Principal Investigator.

The report will include:
• Name of the statistician who performed the analyses and prepared the report
• Name of the methodologist
• Name of the Principal Investigator
• Report title, location, date and version
• Data files and statistical programs used to generate the analyses
