# Supplementary material for: Efficacy and safety of temporary selective portal vein embolization before major hepatectomy (EMBORES): a phase 2 trial
Source: eClinicalMedicine. 2026 Oct 12;101:104262. doi: 10.1016/j.eclinm.2026.104262 (PMC13681468; doi:10.1016/j.eclinm.2026.104262)
Supplement: Supplementary file 7 [file mmc4.pdf]

Full Study Title: Multicenter Prospective Study Evaluating the Efficacy of Reversible Selective Portal Vein Embolization as a Preoperative Strategy in Patients Undergoing Major Hepatectomy

This study is sponsored by

Assistance Publique – Hôpitaux de Paris (AP-HP)
Department of Clinical Research and Development (DRCD)

1 Avenue Claude Vellefaux
75010 Paris
France

PATIENT INFORMATION SHEET

Dear Madam, Sir,

Dr. ………………………………………………. (full name), practicing at ………………………………………………. Hospital, is inviting you to participate in a research study concerning your medical condition.

It is important that you read this information sheet carefully before making your decision. Please do not hesitate to ask your physician for any additional explanations you may require.

If you decide to participate in this study, you will be asked to provide written informed consent.

1. What is the purpose of this study?

You have a liver disease that requires the removal of three or more liver segments (major liver resection).

Before undergoing surgery, you will undergo portal vein embolization. The purpose of this procedure is to increase the volume of the future liver remnant (the healthy part of the liver) so that it can adequately maintain liver function after surgery.

Portal vein embolization consists of occluding the veins supplying the portion of the liver that will be removed. This results in a reduction in the volume of the diseased portion of the liver while increasing the volume of the healthy liver remnant (see diagram below).

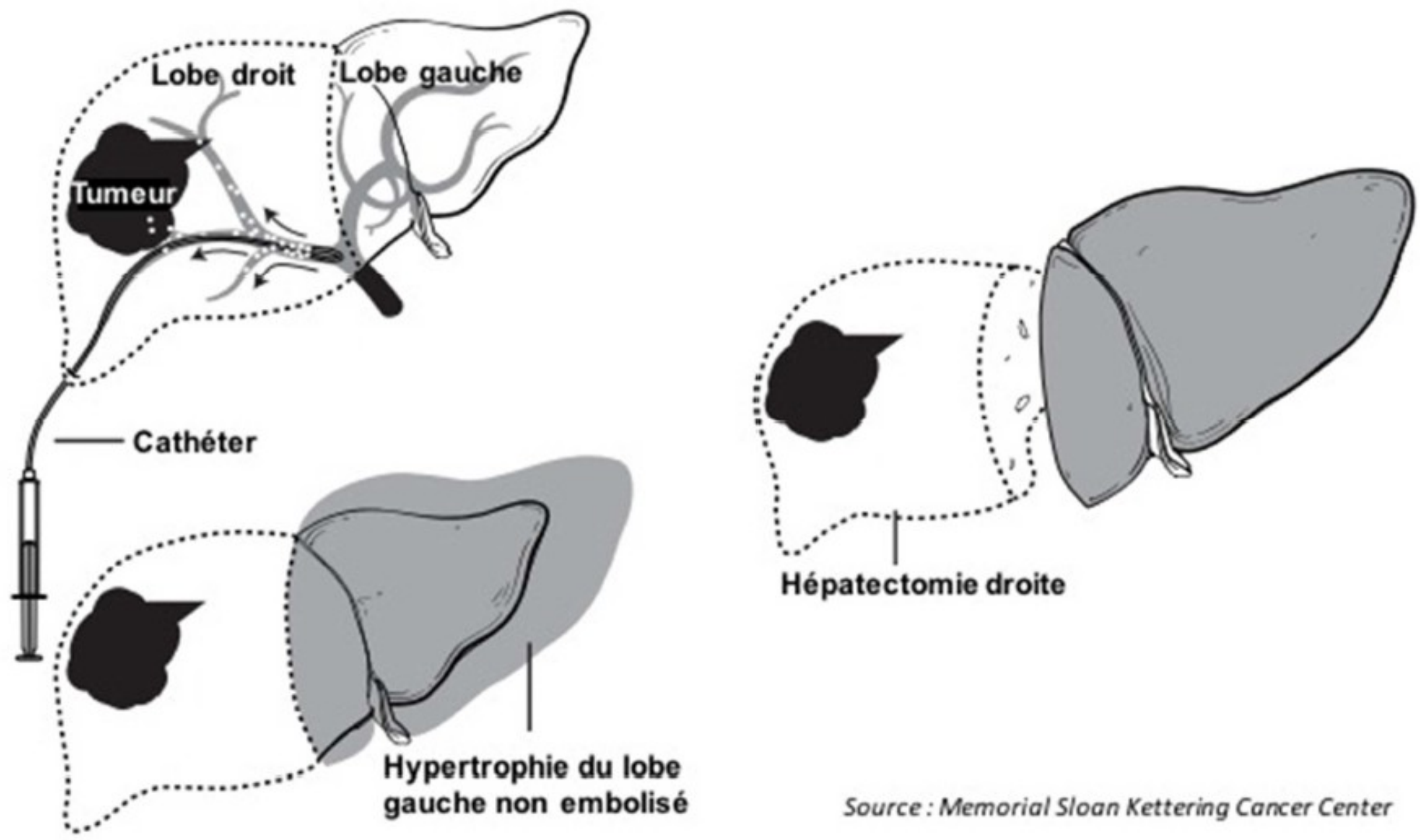

In routine clinical practice, conventional portal vein embolization is performed using non-resorbable embolic agents that are not eliminated by the body. The use of these non-resorbable agents results in permanent occlusion of the portal veins supplying the targeted portion of the liver.

In this study, Curaspon®, a resorbable embolic agent that is naturally eliminated by the body, will be used instead of non-resorbable agents. The aim is to reduce the risk of inadvertent embolization of the healthy portion of the liver while still promoting hypertrophy of the future liver remnant.

The purpose of this study is therefore to demonstrate that portal vein embolization performed with a resorbable embolic agent (reversible embolization) can achieve a similar increase in healthy liver volume compared with conventional portal vein embolization performed with a non-resorbable embolic agent (permanent embolization) in patients requiring major liver resection.

To answer this research question, 50 patients scheduled to undergo portal vein embolization prior to major liver resection will be enrolled in Digestive Surgery Departments across the Paris region (7 participating centers).

2. What does participation involve?

In this study, all patients scheduled to undergo major liver resection will first undergo portal vein embolization in order to increase the volume of the healthy liver remnant.

Your medical management will be identical to the standard care routinely provided to patients with the same condition. You will be hospitalized for one or two days to undergo percutaneous portal vein embolization, meaning that the surgeon or interventional radiologist will access your liver through a needle puncture performed under general anesthesia.

The procedure will begin with a portography, an imaging examination of the portal vein performed after injection of an iodinated contrast agent. This examination requires the introduction of a catheter into the portal vein (the main vein supplying the liver). It allows identification of the portal branches to be embolized using a commonly employed contrast medium (Visipaque®, GE Healthcare).

Subsequently, using a microcatheter inserted through the same access route, Curaspon® will be injected throughout the portal branches selected for embolization.

The embolization technique itself remains unchanged from standard clinical practice. The only difference is that the conventional non-resorbable embolic material, which permanently occludes the selected portal branches, is replaced by a resorbable embolic agent that is naturally eliminated by the body.

3. What is the study schedule?

The overall duration of the study will be 36 months. Your participation will last a maximum of 18 months.

At the screening visit (V0), your physician will determine whether you are eligible to undergo portal vein embolization before major liver resection. During this visit, the study objectives will be explained to you and participation will be proposed.

If you are eligible for portal vein embolization prior to major hepatectomy, you will attend the inclusion visit (V1) on the day of embolization. Your medical management will remain unchanged except for the embolization material used. Portal vein embolization will be performed using the resorbable agent Curaspon® instead of a conventional non-resorbable embolic agent.

A follow-up visit will be performed 4–6 weeks after embolization.

Surgery and postoperative follow-up visits (3 months, 6 months, and 12 months after surgery) will then be carried out according to standard clinical practice for your condition.

4. What are the benefits and constraints associated with participation?

Portal vein embolization performed using a non-resorbable embolic agent may result in thrombus extension (i.e., complete occlusion of the veins supplying blood to the liver), which may subsequently contraindicate liver surgery.

The main expected benefit of reversible portal vein embolization (using a resorbable embolic agent) is a reduction in the complication rate associated with portal vein embolization.

Furthermore, if this technique proves to be as effective and safer than the conventional technique using non-resorbable embolic agents, additional applications may be developed in the future for the treatment of other patients.

In addition, portal vein embolization performed with a resorbable embolic agent does not expose patients to any additional risk compared with the conventional technique using a non-resorbable embolic agent.

Furthermore, in theory, this technique does not expose you to any disadvantage. Indeed, if reversible portal vein embolization proves insufficiently effective, the option of performing a conventional permanent portal vein embolization remains available.

By participating in this study, you will benefit from close medical follow-up at no additional cost to you.

If you agree to participate, you will be required to comply with the following:

• Carry your patient identification card with you at all times. This card indicates that you are participating in this study and includes the name and telephone number of your study physician.

• Attend all scheduled study visits. If you are unable to attend a visit, please contact your physician as soon as possible.

• Inform the study physician of any medication you are taking and of any event occurring during the study (hospitalization, pregnancy, etc.).

• Not participate in any other research study without your physician’s prior approval.

• Be affiliated with a health insurance system or be a beneficiary of such coverage.

5. Which treatments are not permitted during the study?

Anticoagulant medications must be discontinued at least twenty-four hours before embolization (or longer depending on the specific anticoagulant).

The main anticoagulant and antithrombotic medications include:

• Kardegic®
• Lovenox®
• Innohep®
• Arixtra®
• Plavix®
• Coumadin®
• Préviscan®
• Sintrom®
• Pradaxa®
• Xarelto®
• Eliquis®

6. What are the foreseeable risks associated with the study?

The additional risks related to participation in this study are those associated with the use of Curaspon® during portal vein embolization.

Potential complications include:

• Pulmonary embolism, which will be managed according to the standard clinical practice of each participating center.

• Severe allergic reactions to Curaspon® that may require emergency medical treatment. Because Curaspon® contains porcine-derived products, patients with known allergies to porcine products are not eligible to participate in this study.

• Extension of portal vein thrombosis.

7. What are the available medical alternatives?

If you decide not to participate in this study, you will continue to receive appropriate medical care according to the standard practice of your treatment center.

The alternative to portal vein embolization using Curaspon® is conventional portal vein embolization performed with a non-resorbable embolic agent (i.e., an agent that permanently occludes part of the liver vascular supply), as is routinely performed in clinical practice.

The specific embolic material used may vary from one center to another.

8. What medical care will be provided at the end of your participation?

If your participation is discontinued prematurely (for example, because of anesthesia-related complications, inability to access the portal vein, inability to perform portal vein embolization, or significant bleeding), your physician will discuss alternative treatment options that correspond to the current standard of care for your liver disease.

The follow-up initially planned for the study will nevertheless continue.

At the end of your participation, you will continue to receive appropriate medical follow-up according to the standard practice of your treatment center.

10. If you participate, what will happen to the data collected during the study?

As part of the biomedical research study proposed to you by AP-HP, your personal data will be processed in order to analyze the study results in relation to the research objectives that have been explained to you.

For this purpose, medical information concerning you, as well as information relating to your lifestyle and health habits, will be transmitted to the Sponsor of the study. These data will be identified only by a study code number and your initials.

Under conditions ensuring strict confidentiality, these data may also be transmitted to the French health authorities.

If you discontinue participation without withdrawing your consent, the data collected before your withdrawal may continue to be used unless you specifically object.

11. How is this study regulated?

AP-HP has obtained insurance coverage (Policy No. 0100518814033-160051-10998) guaranteeing its civil liability and that of all individuals involved in the study through HDI–GERLING, represented by BIOMEDICINSURE, whose address is:

Parc d'Innovation Bretagne Sud
C.P. 142
56038 Vannes Cedex
France

AP-HP has implemented all measures required by French legislation governing the protection of individuals participating in biomedical research, namely the Huriet Law (No. 88-1138 of December 20, 1988), as amended by the Public Health Law (No. 2004-806 of August 9, 2004).

AP-HP obtained a favorable opinion from the French Research Ethics Committee (Comité de Protection des Personnes, CPP Île-de-France VII) for this study on February 7, 2017, as well as authorization from the French National Agency for Medicines and Health Products Safety (ANSM).

12. What are your rights?

Your participation in this study is entirely voluntary.

Your decision whether or not to participate will not affect the quality of care or treatment that you are entitled to receive.

Throughout the study, you may request additional information about the conduct of the study from the physician responsible for your care.

You may withdraw from the study at any time without providing any justification, without affecting your future treatment, the quality of care you receive, or your relationship with your physician. Following withdrawal, you may continue to be followed by the same medical team.

In accordance with French data protection regulations (CNIL – Data Protection Act), you have the right to access and correct personal information concerning you.

You also have the right to object to the transmission and processing of confidential information covered by professional secrecy that may be used for the purposes of this research. These rights may be exercised through the physician responsible for the study, who is the only person who knows your identity within the research database.

In accordance with Article L1111-7 of the French Public Health Code, you may also access all of your medical information directly or through a physician of your choice.

Your medical records will remain strictly confidential and may only be reviewed under the responsibility of the physician in charge of your treatment, by the health authorities, and by individuals specifically authorized by AP-HP for study monitoring purposes and bound by professional confidentiality obligations.

At the end of the study, once the data have been analyzed, you may obtain information regarding the overall study results through the physician responsible for your participation in this research.

If, after reading all of this information and discussing every aspect of the study with your physician, you agree to participate, you will be asked to sign and date the informed consent form located at the end of this document.

INFORMED CONSENT FORM

I, the undersigned, Mrs / Ms / Mr (delete as appropriate)

Surname and First Name: ____

freely agree to participate in the research study entitled:

“Multicenter Prospective Study Evaluating the Efficacy of Reversible Selective Portal Vein Embolization as a Preoperative Strategy in Patients Undergoing Major Hepatectomy”

organized by Assistance Publique – Hôpitaux de Paris (AP-HP) and proposed to me by:

Dr. ____
(Name, First Name, Telephone Number)

acting as an investigator in this study.

I confirm that:

- I have read the Patient Information Sheet, Version 1.1 dated November 28, 2016, which explained the purpose of this study, how it will be conducted, and what my participation involves.

- I will retain a copy of the Patient Information Sheet and this Informed Consent Form.

- I have received satisfactory answers to all of my questions.

- I have been given sufficient time to make my decision.

- I understand that my participation is voluntary and that I may withdraw from the study at any time without incurring any liability and without affecting the quality of care that I will receive. If I decide to withdraw, I will inform the physician responsible for my care whether or not I wish the data collected up to the time of my withdrawal to continue to be used.

- I understand that my participation may also be discontinued by the investigator if necessary.

- Prior to participating in this study, I underwent a medical examination appropriate for the study, and the results were explained to me.

- I understand that, in order to participate in this study, I must be affiliated with a health insurance system or be a beneficiary of such coverage. I confirm that this is the case.

- I have been informed that my participation in this study will last a maximum of 18 months and that, due to the one-month exclusion period, I should not participate in another research study within 19 months without informing the physician responsible for this study.

- I understand that my consent does not release either the investigator or AP-HP from any of their responsibilities, and that I retain all rights guaranteed to me by law.

Participant

Surname and First Name: ____

Date: ___________________________

Signature: _______________________

Investigator

Surname and First Name: ____

Date: ___________________________

Signature: _______________________

This document must be completed in triplicate. The original copy must be retained by the investigator for the legally required retention period, the second copy must be provided to the participant, and the third copy must be sent to AP-HP in a sealed envelope at the conclusion of the study.
