# Supplementary material for: Efficacy and safety of temporary selective portal vein embolization before major hepatectomy (EMBORES): a phase 2 trial
Source: eClinicalMedicine. 2026 Oct 12;101:104262. doi: 10.1016/j.eclinm.2026.104262 (PMC13681468; doi:10.1016/j.eclinm.2026.104262)
Supplement: Supplementary file 8 [file mmc5.pdf]

# FAVORABLE OPINION OF THE ETHICS COMMITTEE (CPP)

## Research Project No. 16-028

July 18, 2019

Multicenter Prospective Study Evaluating the Efficacy of Reversible Selective Portal Vein Embolization as a Preoperative Strategy in Patients Undergoing Major Hepatectomy (EMBORES – ID■RCB No. 2016■A00878■43).

Substantial Amendment No. 4 reviewed June 12, 2019. Update of investigators list and replacement of Dr Planche by Dr Debry.

The Committee considered that the amendment did not modify either the methodological integrity of the study or the protection of participating subjects.

# FAVORABLE OPINION WITHOUT RESTRICTIONS

For Michel BOTTLAENDER, Chair of the session of June 12, 2019

Comité de Protection des Personnes
Ile-de-France VII

Hôpital de Bicêtre – 78 rue du général Leclerc – 94275 Le Kremlin Bicêtre Cedex
Présidente : Anne-Marie TABURET – Secrétaire : Brigitte PILATE

Projet de Recherche N° : *16-028* Kremlin Bicêtre le 18 juillet 2019

Le Comité a été saisi le 5 juin 2019

D'une modification substantielle par Madame FAVREL FEUILLADE du DRCD – Hôpital Saint Louis – 1 avenue Claude Vellefaux – 75475 PARIS CEDEX 10 pour le compte du promoteur AP-HP – 3 avenue Victoria – 75004 PARIS concernant le projet de recherche qui avait obtenu un avis favorable du C.P.P. le 11 août 2015, le 26 juillet 2016, le 10 mai 2017, le 17 juillet 2018, le 10 octobre 2018 et le 9 janvier 2019 et intitulé :
**«ETUDE PROSPECTIVE MULTICENTRIQUE EVALUANT L'EFFICACITE DE L'EMBOLIE PORTALE SELECTIVE REVERSIBLE EN PREOPERATOIRE CHEZ LES PATIENTS DEVANT BENEFICIER D'UNE HEPATECTOMIE MAJEURE »**
(Référence du promoteur : EMBORES - ID-RCB : n° 2016-A00878-43)

dont l'investigateur principal est le Professeur DAGHER – Service de Chirurgie Digestive – Hôpital Antoine Béclère – 157 rue de la Porte de Trivaux – 92141 CLAMART Cedex

Le comité a examiné la modification substantielle n° 4 datée du 7 mai 2019 à la séance du 12 juin 2019 et portant sur la mise à jour de la liste des investigateurs version du 7/05/2019 avec le remplacement du Docteur PLANCHE par le Docteur DEBRY

**Membres présents lors de la délibération de votre protocole**

Premier Collège :
- Recherche biomédicale : Monsieur M. BOTTLAENDER (T), Monsieur G. P. DE FILIPPO, pédiatre (T), Monsieur R. de BEAUREPAIRE (S) et Madame K. BOURDIC (S)
- Médecin généraliste : Monsieur E. DEFLESSELLE (S)
- Pharmacien : Madame A. M. TABURET (T)

Deuxième collège :
- Personne qualifiée en éthique : Monsieur P. CASAURANG (T)
- Juriste : Madame F. BOISSY (T) et Madame V.A. LAFOY (T)
- Associations agréées : Monsieur C. COTTET (T) et Madame A. LABBE (T)

Le Comité :
- considérant que l'amendement en référence ne modifie ni le respect méthodologique, ni la protection des personnes participant à l'étude

a adopté la délibération suivante :

**AVIS FAVORABLE sans restriction**

AMTaburet

pour Michel BOTTLAENDER
Président de la séance du 12 juin 2019
