# Supplementary material for: Efficacy and safety of temporary selective portal vein embolization before major hepatectomy (EMBORES): a phase 2 trial
Source: eClinicalMedicine. 2026 Oct 12;101:104262. doi: 10.1016/j.eclinm.2026.104262 (PMC13681468; doi:10.1016/j.eclinm.2026.104262)
Supplement: Supplementary file 9 [file mmc6.pdf]

FAVORABLE OPINION OF THE ETHICS COMMITTEE (CPP)

Research Project No. 16-028

January 9, 2019

Multicenter Prospective Study Evaluating the Efficacy of Reversible Selective Portal Vein Embolization as a Preoperative Strategy in Patients Undergoing Major Hepatectomy (EMBORES – ID■RCB No. 2016■A00878■43).

Substantial Amendment No. 3 reviewed December 5, 2018. Update of investigators list and GDPR-compliant patient information and consent form.

The Committee considered that the amendment did not modify either the methodological integrity of the study or the protection of participating subjects.

FAVORABLE OPINION WITHOUT RESTRICTIONS

Anne■Marie TABURET, Chair of the session of December 5, 2018

Comité de Protection des Personnes
Ile-de-France VII

Hôpital de Bicêtre – 78 rue du général Leclerc – 94275 Le Kremlin Bicêtre Cedex
Présidente : Anne-Marie TABURET – Secrétaire : Brigitte PILATE

Projet de Recherche N° : *16-028* Kremlin Bicêtre le 9 janvier 2019

Le Comité a été saisi le 22 novembre 2018

D'une modification substantielle par Madame FAVREL FEUILLADE du DRCD – Hôpital Saint Louis – 1 avenue Claude Vellefaux – 75475 PARIS CEDEX 10 pour le compte du promoteur AP-HP – 3 avenue Victoria – 75004 PARIS concernant le projet de recherche qui avait obtenu un avis favorable du C.P.P. le 11 août 2015, le 26 juillet 2016, le 10 mai 2017, le 17 juillet 2018 et le 10 octobre 2018 et intitulé :

«ETUDE PROSPECTIVE MULTICENTRIQUE EVALUANT L'EFFICACITE DE L'EMBOLIE PORTALE SELECTIVE REVERSIBLE EN PREOPERATOIRE CHEZ LES PATIENTS DEVANT BENEFICIER D'UNE HEPATECTOMIE MAJEURE »
(Référence du promoteur : EMBORES - ID-RCB : n° 2016-A00878-43)

dont l'investigateur principal est le Professeur DAGHER – Service de Chirurgie Digestive – Hôpital Antoine Béclère – 157 rue de la Porte de Trivaux – 92141 CLAMART Cedex

Le comité a examiné la modification substantielle n° 3 datée du 13 novembre 2018 à la séance du 05/12/2018 et portant sur la mise à jour de la liste des investigateurs version du 13/10/et la mise en conformité avec le RGPD avec nouvelle lettre d'information et consentement version N° 2.1 du 14/12/2018 modifiée à la demande du CPP

<u>Membres présents lors de la délibération de votre protocole</u>
Premier Collège :
- Recherche biomédicale : Monsieur P. de BOISSIEU, épidémiologiste (T), Monsieur M. BOTTLAENDER (T) Madame K. BOURDIC (S) et Monsieur de FILIPPO, pédiatre (T)
- Médecin généraliste : Monsieur G. COINDARD (T)
- Pharmacien : Madame A. M. TABURET (T)

Deuxième collège :
- Personne qualifiée en éthique : Monsieur P. CASAURANG (T)
- Juriste : Madame F. BOISSY (T) et Madame V. A. LAFOY (T)
- Associations agréées : Monsieur C. COTTET (T) et Madame A. LABBE (T)

Le Comité :
- considérant que l'amendement en référence ne modifie ni le respect méthodologique, ni la protection des personnes participant à l'étude

a adopté la délibération suivante :

AVIS FAVORABLE sans restriction

Anne-Marie TABURET
Président de la séance du 5 décembre 2018
