# Supplementary material for: Efficacy and safety of temporary selective portal vein embolization before major hepatectomy (EMBORES): a phase 2 trial
Source: eClinicalMedicine. 2026 Oct 12;101:104262. doi: 10.1016/j.eclinm.2026.104262 (PMC13681468; doi:10.1016/j.eclinm.2026.104262)
Supplement: Supplementary file 10 [file mmc7.pdf]

FAVORABLE OPINION OF THE ETHICS COMMITTEE (CPP)

Research Project No. 16-028

October 10, 2018

Multicenter Prospective Study Evaluating the Efficacy of Reversible Selective Portal Vein Embolization as a Preoperative Strategy in Patients Undergoing Major Hepatectomy (EMBORES – ID■RCB No. 2016■A00878■43).

Substantial Amendment No. 2 reviewed October 3, 2018. Update of investigators list, removal of non-inclusion criteria, and conversion of radiology centers into recruiting centers.

The Committee considered that the amendment did not modify either the methodological integrity of the study or the protection of participating subjects.

FAVORABLE OPINION WITHOUT RESTRICTIONS

François HIRSCH, Chair of the session of October 3, 2018

Comité de Protection des Personnes
Ile-de-France VII

Hôpital de Bicêtre – 78 rue du général Leclerc – 94275 Le Kremlin Bicêtre Cedex
Présidente : Anne-Marie TABURET – Secrétaire : Brigitte PILATE

Projet de Recherche N° : *16-028* Kremlin Bicêtre le 10 octobre 2018

Le Comité a été saisi le 4 septembre 2018

D'une modification substantielle par Par Madame FAVREL FEUILLADE du DRCD – Hôpital Saint Louis – 1 avenue Claude Vellefaux – 75475 PARIS CEDEX 10 pour le compte du promoteur AP-HP – 3 avenue Victoria – 75004 PARIS concernant le projet de recherche qui avait obtenu un avis favorable du C.P.P. le 11 août 2015, le 26 juillet 2016, le 10 mai 2017 et le 17 juillet 2018 et intitulé : **«ETUDE PROSPECTIVE MULTICENTRIQUE EVALUANT L'EFFICACITE DE L'EMBOLIE PORTALE SELECTIVE REVERSIBLE EN PREOPERATOIRE CHEZ LES PATIENTS DEVANT BENEFICIER D'UNE HEPATECTOMIE MAJEURE »**
(Référence du promoteur : EMBORES - ID-RCB : n° 2016-A00878-43)

dont l'investigateur principal est le Professeur DAGHER – Service de Chirurgie Digestive – Hôpital Antoine Béclère – 157 rue de la Porte de Trivaux – 92141 CLAMART Cedex

Le comité a examiné la modification substantielle n° 2 datée du 14 août 2018 (protocole version 3.0 du 14/08/2018 et résumé version 3.0 du 14/08/5018) à la séance du 03/10/2018 et portant sur la mise à jour de la liste des investigateurs version du 14/08/2018, la suppression de critères de non inclusion et la modification du statut des centres de radiologie qui deviennent des centres recruteurs

Membres présents lors de la délibération de votre protocole

Premier Collège :
- Recherche biomédicale : Monsieur P. de BOISSIEU, épidémiologiste (T), Monsieur F. HIRSCH (T), Monsieur M. BOTTLAENDER (T) et Monsieur de FILIPPO, pédiatre (T)
- Médecin généraliste : Monsieur G. COINDARD (T)
- Pharmacien : Madame D. BLONDELON (S)

Deuxième collège :
- Personne qualifiée en éthique : Monsieur P. CASAURANG (T)
- Juriste : Madame F. BOISSY (T) et Madame V. A. LAFOY (T)
- Associations agréées : Monsieur C. COTTET (T) et Madame A. LABBE (T)

Le Comité :
- considérant que l'amendement en référence ne modifie ni le respect méthodologique, ni la protection des personnes participant à l'étude

a adopté la délibération suivante :

AVIS FAVORABLE sans restriction

François HIRSCH
Président de la séance du 3 octobre 2018
