# Supplementary material for: Efficacy and safety of temporary selective portal vein embolization before major hepatectomy (EMBORES): a phase 2 trial
Source: eClinicalMedicine. 2026 Oct 12;101:104262. doi: 10.1016/j.eclinm.2026.104262 (PMC13681468; doi:10.1016/j.eclinm.2026.104262)
Supplement: Supplementary file 12 [file mmc9.pdf]

FAVORABLE OPINION OF THE ETHICS COMMITTEE (CPP)

Research Project No. 16-028

Kremlin-Bicêtre, June 8, 2020

The Committee was notified on November 27, 2019 of a substantial amendment submitted on behalf of AP■HP concerning the EMBORES study.

Multicenter Prospective Study Evaluating the Efficacy of Reversible Selective Portal Vein Embolization as a Preoperative Strategy in Patients Undergoing Major Hepatectomy (EMBORES – ID■RCB No. 2016■A00878■43).

Principal Investigator: Professor DAGHER, Department of Digestive Surgery, Antoine Béclère Hospital.

The Committee reviewed Substantial Amendment No. 5. The amendment included an update of the investigators list, a reduction by half of the required sample size for the primary objective, a 12■month extension of the inclusion period, clarification of study procedures when surgery is not performed or when no anesthesiology consultation has occurred, and updates to the patient information sheet and informed consent form for GDPR compliance.

Committee Conclusion

Considering that the amendment does not modify either the methodological integrity of the study or the protection of participating subjects, the Committee adopted the following decision:

FAVORABLE OPINION WITHOUT RESTRICTIONS

For François HIRSCH

Chair of the Session of March 11, 2020

Comité de Protection des Personnes
Ile-de-France VII
Hôpital de Bicêtre – 78 rue du général Leclerc – 94275 Le Kremlin Bicêtre Cedex
Présidente : Anne-Marie TABURET – Secrétaire : Brigitte PILATE

Projet de Recherche N° : ***16-028*** Kremlin Bicêtre le 8 juin 2020

Le Comité a été saisi le 27 novembre 2019
D'une modification substantielle par Madame FAVREL FEUILLADE du DRCD – Hôpital Saint Louis – 1 avenue Claude Vellefaux – 75475 PARIS CEDEX 10 pour le compte du promoteur AP-HP – 3 avenue Victoria – 75004 PARIS concernant le projet de recherche qui avait obtenu un avis favorable du C.P.P. le 11 août 2015, le 26 juillet 2016, le 10 mai 2017, le 17 juillet 2018, le 10 octobre 2018 et le 9 janvier 2019 et le 18 juillet 2019 et intitulé :
«ETUDE PROSPECTIVE MULTICENTRIQUE EVALUANT L'EFFICACITE DE L'EMBOLIE PORTALE SELECTIVE REVERSIBLE EN PREOPERATOIRE CHEZ LES PATIENTS DEVANT BENEFICIER D'UNE HEPATECTOMIE MAJEURE »
(Référence du promoteur : EMBORES - ID-RCB : n° 2016-A00878-43)

dont l'investigateur principal est le Professeur DAGHER – Service de Chirurgie Digestive – Hôpital Antoine Béclère – 157 rue de la Porte de Trivaux – 92141 CLAMART Cedex

Le comité a examiné la modification substantielle n° 5 datée du 27 novembre 2019 (protocole version 4.2 du 04/02/2020 et résumé version 4.2 du 04/02/2020) aux séances du 11 décembre 2019, du 15 janvier 2020 et du 13 mars 2020 et portant sur la mise à jour de la liste des investigateurs (addendum 1 du protocole), la réduction de moitié du nombre de patients requis pour répondre à l'objectif principal, la prolongation de la période d'inclusion de 12 mois supplémentaires, l'ajout de précision sur la procédure de la recherche en cas de chirurgie non faite et absence de consultation par un anesthésiste et la mise à jour de la note d'information et du formulaire de consentement pour mise en conformité avec le RGPD version 3.2 du 04/02/2020 avec attestation d'assurance du 25/11/2019, courrier argumentaire de l'investigateur coordonnateur du 13/11/2019, FAMS du 26/11/2019 et tableau comparatif de la MS5

Membres présents lors de la délibération de votre protocole
Premier Collège :
- Recherche biomédicale : Monsieur P. de BOISSIEU, épidémiologiste (T), Monsieur M. BOTTLAENDER (T), Monsieur F. HIRSCH (T) et Madame C. HILL (S)
- Pharmacien : Madame A. M. TABURET (T)

Deuxième collège :
- Juriste : Madame F. BOISSY (T) et Madame J. GAUTIER (S)
- Associations agréées : Monsieur C. COTTET (T) et Monsieur G. MARDUEL (S)

Le Comité :
- considérant que l'amendement en référence ne modifie ni le respect méthodologique, ni la protection des personnes participant à l'étude

a adopté la délibération suivante :

AVIS FAVORABLE sans restriction

Pour François HIRSCH
Président de la séance du 11 mars 2020
