# Supplementary material for: Efficacy and safety of temporary selective portal vein embolization before major hepatectomy (EMBORES): a phase 2 trial
Source: eClinicalMedicine. 2026 Oct 12;101:104262. doi: 10.1016/j.eclinm.2026.104262 (PMC13681468; doi:10.1016/j.eclinm.2026.104262)
Supplement: Supplementary file 13 [file mmc13.docx]

# Supplementary Table S1

Exploratory analyses of liver hypertrophy according to portal vein recanalization and embolization technique

## A. Hypertrophy according to the degree of portal vein recanalization

| Degree of portal vein recanalization | n | Mean hypertrophy ratio (%) |
| --- | --- | --- |
| Complete recanalization | 17 | 9.5 |
| Partial recanalization | 9 | 7.6 |
| No recanalization | 6 | 12.8 |

## B. Degree of portal vein recanalization according to embolization technique

| Embolization technique | n | Complete recanalization n (%) | Partial recanalization n (%) | No recanalization n (%) |
| --- | --- | --- | --- | --- |
| Distal only | 9 | 6 (66.7) | 0 (0.0) | 3 (33.3) |
| Proximal only | 3 | 3 (100) | 0 (0.0) | 0 (0.0) |
| Combined distal and proximal | 20 | 8 (40.0) | 9 (45.0) | 3 (15.0) |

**Footnote:** Hypertrophy ratios were calculated using standardized FLR volumes. Negative hypertrophy ratios were conservatively analysed as absence of hypertrophy (0%). Owing to the limited sample size, these analyses are exploratory and no formal statistical comparisons were performed.
