# Supplementary material for: Efficacy and safety of temporary selective portal vein embolization before major hepatectomy (EMBORES): a phase 2 trial
Source: eClinicalMedicine. 2026 Oct 12;101:104262. doi: 10.1016/j.eclinm.2026.104262 (PMC13681468; doi:10.1016/j.eclinm.2026.104262)
Supplement: Supplementary file 14 [file mmc14.docx]

**Supplementary Material.**

**Members of the EMBORES Study Group.**

The following investigators participated in the EMBORES study and are listed as collaborators:

**Christian SENGEL**: CHU Grenoble Alpes, La Tronche, France

**Antoine BROUQUET**: Bicêtre Hospital, Le Kremlin-Bicêtre, France

**Brice GAYET**: Institut Mutualiste Montsouris, Paris, France

**Valérie VILGRAIN**: Beaujon Hospital, Clichy, France

**François CAUCHY**: Beaujon Hospital, Clichy, France

**Olivier SOUBRANE**: Beaujon Hospital, Clichy, France

**Leonor BENHAIM**: Gustave Roussy Cancer Center, Villejuif, France

**Dimitri TZANIS**: Institut Curie, Paris, France

**Yves MENU**: Saint Antoine Hospital, Paris, France

**Sébastien GAUJOUX**: Pitié-Salpêtrière Hospital, Paris, France

**Laurence ROCHER**: Antoine Béclère Hospital, Clamart, France

**Georges RAFEK**: Antoine Béclère Hospital, Clamart, France

**Pierre CHAGUE**: Antoine Béclère Hospital, Clamart, France

**Thierry DE BAERE**: Gustave Roussy Cancer Center, Villejuif, France

**Diane GOERE**: Gustave Roussy Cancer Center, Villejuif, France

**Mickaël LESURTEL**: Beaujon Hospital, Clichy, France

**Panagiotis LAINAS**: Antoine Béclère Hospital, Clamart, France

**Martin GAILLARD**: Antoine Béclère Hospital, Clamart, France

**Joseph DERIENNE**: Antoine Béclère Hospital, Clamart, France

**Marco DIOGUARDI BURGIO**: Beaujon Hospital, Clichy, France

**Annie SIBERT**: Beaujon Hospital, Clichy, France

**Alain ABDO**: Bicêtre Hospital, Le Kremlin-Bicêtre, France

**Teodor VASILE**: Bicêtre Hospital, Le Kremlin-Bicêtre, France

**Severine FOUCHER**: Antoine Béclère Hospital, Clamart, France

**Marie CHALOPIN**: Beaujon Hospital, Clichy, France

**Thibault CHOUQUET**: Gustave Roussy Cancer Center, Villejuif, France

**Sandra CAMPS**: Institut Mutualiste Montsouris, Paris, France

**Steve SUZZONI**: Bicêtre Hospital, Le Kremlin-Bicêtre, France

**Pierrick BEDOUCH**: CHU Grenoble Alpes, La Tronche, France

**Marylise ADECHIAN**: Bicêtre Hospital, Le Kremlin-Bicêtre, France

**Hélène AGOSTINI**: Bicêtre Hospital, Le Kremlin-Bicêtre, France

**Lamiae GRIMALDI**: Bicêtre Hospital, Le Kremlin-Bicêtre, France

**Kawther NHERI**: Bicêtre Hospital, Le Kremlin-Bicêtre, France

**Fatah TIDADINI**: CHU Grenoble Alpes, La Tronche, France

**Olivier SOUBRANE**: Bicêtre Hospital, Le Kremlin-Bicêtre, France

**Jean-Baptiste DEBRY**: Bicêtre Hospital, Le Kremlin-Bicêtre, France
